# Flexible Multimodal Neural Probe with Integrated Three-Electrode Aptameric Sensing for *In Vivo* Monitoring of Dopamine Dynamics and Neural Activity

**DOI:** 10.1021/acschemneuro.6c00550

**Published:** 2026-08-06

**Authors:** Szu-Ying Li, Yun-Ting Kuo, Sheng-Huang Lin, Shun-An Kan, Bo-Wei Chen, Ssu-Ju Li, Ching-Wen Chang, Han-Lin Wang, Yu-Chun Lo, You-Yin Chen

**Affiliations:** † Department of Biomedical Engineering, 34914 National Yang Ming Chiao Tung University, No.155, Sec.2, Linong St., Taipei City 112304, Taiwan; ‡ Department of Neurology, 63136 Hualien Tzu Chi Hospital, Buddhist Tzu Chi Medical Foundation, No. 707, Sec. 3, Zhongyang Rd., Hualien City 97002, Taiwan; § Department of Neurology, School of Medicine, Tzu Chi University, No. 701, Sec. 3, Zhongyang Rd., Hualien City 97004, Taiwan; ∥ Department of Education, 46615 Taipei Veterans General Hospital, No.201, Sec. 2, Shipai Rd., Taipei City 11217, Taiwan; ⊥ Ph.D. Program in Medical Neuroscience, College of Medical Science and Technology, Taipei Medical University, 12F., Education & Research Building, Shuang-Ho Campus, No. 301, Yuantong Rd., New Taipei City 23564, Taiwan

**Keywords:** dopamine sensing, electrochemical aptamer sensor, integrated three-electrode neural probe, multimodal neurochemical and electrophysiological recording, flexible microelectrode array, *in vivo* neurochemistry

## Abstract

Neural function emerges from the interplay between electrical activity and neurochemical signaling, yet most implantable neural interfaces primarily record electrophysiological signals and lack molecular specificity for neurotransmitter monitoring. Here, we developed a flexible multimodal neural probe integrating an on-chip three-electrode electrochemical aptamer sensor with electrophysiological recording sites for combined monitoring of extracellular dopamine (DA) dynamics and neuronal activity. Fabrication of the electrochemical interface was systematically optimized by controlling gold nanostructure (AuNS) electrodeposition from 0.50 to 0.70 V. Electrodeposition at 0.65 V provided the best balance between increased electrochemically active surface area (ECSA), surface morphology, fabrication reproducibility, and electrical isolation, yielding an approximately 4.7-fold ECSA enhancement with a probe-to-probe coefficient of variation (CV%) of 2.02%. A methylene blue-labeled DA aptamer was assembled onto the AuNS working electrodes for sequence-specific molecular recognition, while integrated Ag/AgCl reference and nanostructured platinum counter electrodes completed the on-chip sensing system. Electrochemical impedance analysis further demonstrated reproducible interfacial characteristics following stepwise functionalization. The electrophysiological electrodes exhibited a mean impedance of 366.02 ± 18.63 kΩ at 1 kHz with an interprobe CV% of 5.09%. Square-wave voltammetry frequency was experimentally optimized from 10 to 200 Hz, with 100 Hz providing the best balance between analytical response, background current, and reproducibility. Under optimized conditions, the sensor demonstrated sequence-specific DA recognition, selectivity against electroactive interferents, and an experimentally determined detection limit of 10 fM. Two absolute linear response regions were identified at 0.5–10 pM (*R* ^2^ = 0.9945) and 0.5–10 nM (*R* ^2^ = 0.9949), while the broader detectable concentration range extended from 10 fM to 1 μM. Flow-injection and brain-phantom experiments demonstrated reversible DA sensing. In acute rat caudate-putamen experiments, intravenous nomifensine induced DA-associated electrochemical responses accompanied by increased neuronal firing and γ/high-γ local field potential activity, supporting this platform for acute multimodal neurochemical and electrophysiological interrogation *in vivo*.

## Graphical abstract

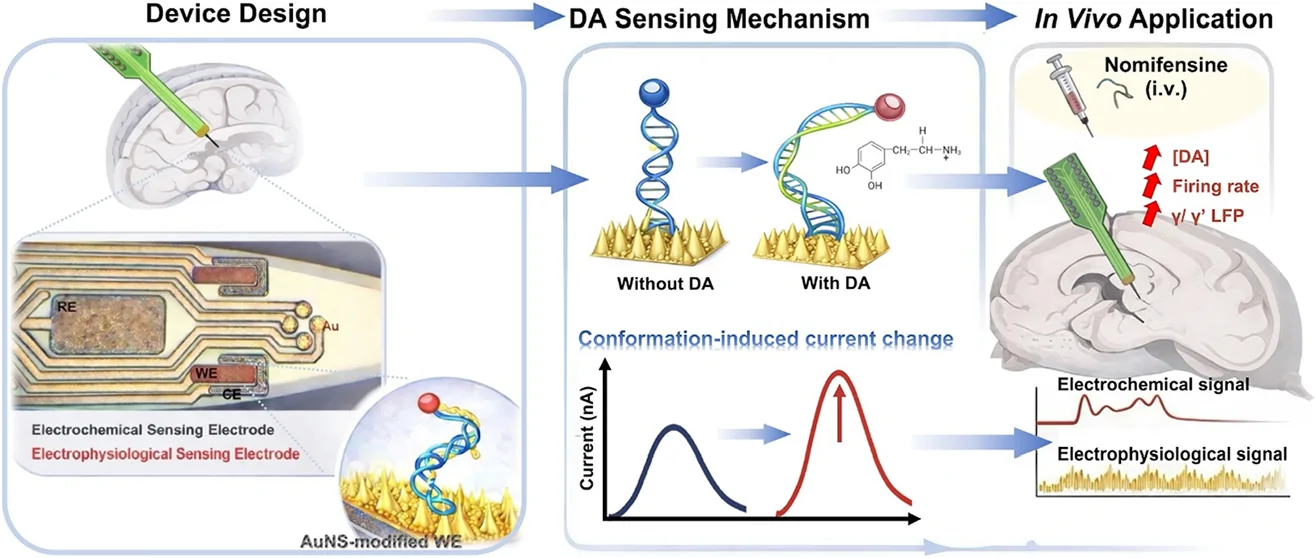

## Introduction

Neural information processing is governed by the coordinated interplay between fast electrical signaling and slower, spatially heterogeneous chemical neurotransmission within local brain microenvironments.^1^ While electrophysiological recordings provide high-temporal resolution access to neuronal spiking activity and local field potentials (LFPs),^2^ chemical signaling mediated by neurotransmitters conveys complementary information related to synaptic efficacy, neuromodulatory tone, and state-dependent circuit modulation.^3^ Disruption of this electrochemical coupling has been implicated in a wide range of neurological and neuropsychiatric disorders,^4^ motivating the development of neural interfaces capable of interrogating both electrical and chemical dimensions of brain activity *in vivo*.

Over the past decade, implantable electrophysiological recording technologies have advanced substantially, with progress enabling high channel counts, dense spatial coverage, and increasingly stable chronic operations. Meanwhile, neural interface research has increasingly shifted toward multifunctional sensing platforms that extend beyond purely electrical readouts to incorporate neurochemical measurements.^5–7^ Among the strategies explored for such integration, electrochemical sensing has emerged as the most widely adopted modality. This trend is driven by its intrinsic compatibility with miniaturized electrodes, scalability through established microfabrication processes, and capacity for real-time operation under physiological conditions.^7,8^ As a result, electrochemical approaches now occupy a central position in the development of integrated neural interfaces that combine electrophysiology with chemically selective detection in a unified and minimally invasive architecture.^6,9^

Electrochemical sensing techniques based on amperometric^10^ and voltammetric^11,12^ transduction generate current signals through oxidation–reduction reactions of electroactive species and are therefore well-suited for *in vivo* neurotransmitter detection. However, within the complex biochemical environment of the brain, chemical specificity remains a persistent challenge.^13^ Constant-potential amperometry offers excellent temporal resolution and straightforward miniaturization, yet chemical selectivity is intrinsically limited because any electroactive species that undergoes oxidation (or reduction) within the imposed potential window contributes to the measured current,^8,14^ yielding a composite signal in complex brain extracellular environments.

In contrast, voltammetric readouts can provide richer electrochemical signatures, but their *in vivo* implementation is often dominated by large nonfaradaic/background currents and time-dependent drift,^15^ such that reliable quantification typically requires background subtraction and careful treatment of baseline evolution during prolonged recordings.^16^ Moreover, even with pulse-based voltammetry designed to enhance faradaic contributions, redox features from coexisting interferents may overlap with the analyte response,^8^ increasing ambiguity in peak assignment and chemical attribution under physiological conditions.

Under these constraints, limitations intrinsic to electrochemical transduction are further amplified by the physical requirements of the implantable neural interfaces. To reduce tissue damage and mitigate chronic inflammatory responses, electrodes are necessarily miniaturized to micrometer dimensions, which drastically restricts both the effective sensing area and the analyte sampling volume. Because the magnitude of the faradaic current scales with electrode surface area, electrochemical signals recorded from microscale electrodes are inherently small.^17^ In addition, electrode miniaturization is widely recognized to increase interfacial impedance, which elevates thermal (Johnson-Nyquist) noise and susceptibility to electromagnetic interference, thereby severely degrading the signal-to-noise ratio.^18^ Beyond these electrical considerations, large concentration disparities are commonly encountered between target neurotransmitters and endogenous electroactive interferents (e.g., ascorbic acid and uric acid),^13,19^ which are often present at substantially higher levels in brain biofluids. Under such conditions, background currents arising from high-abundance interferents can dominate the measured response, further obscuring analyte-specific signals and substantially increasing uncertainty in chemical attribution.^20^ In response to these coupled physical, electrochemical, and biological constraints, molecular recognition-based sensing strategies have attracted increasing attention. In particular, aptamer-based sensing interfaces have emerged as promising approaches for *in vivo* neurochemical detection. Aptamers are synthetically engineered single-stranded nucleic acids that exhibit high target specificity, reversible binding, and robust chemical stability without reliance on enzymatic cofactors.^21^ When immobilized on electrode surfaces, target-induced conformational changes of aptamers can be efficiently transduced into electrochemical signals, enabling selective detection of neurotransmitters and neuropeptides, including species that are weakly electroactive or electrochemically inactive. Under the severe miniaturization constraints characteristic of implantable neural interfaces, the primary advantage of aptamer-modified electrochemical sensors lies in their ability to achieve high chemical selectivity and low detection limits rather than in the elimination of long-term stability challenges inherent to any functionalized interface.

To date, aptamer-based neurochemical sensing platforms have primarily been realized using two transduction strategies: aptamer-functionalized field-effect transistors (FETs)^22–24^ and electrochemical aptamer-based electrodes.^12,25,26^ In FET-based implementations, target binding is converted to modulation of surface charge, enabling label-free and highly sensitive detection. However, in implantable neural interfaces, stable operation is often complicated by reliance on external reference electrodes,^8^ pronounced sensitivity to ionic strength variations,^23,27^ susceptibility to surface charge drift,^28^ and long-term biofouling.^29^ These characteristics are particularly problematic in high-ionic-strength physiological environments and during chronic implantation.^22^

By contrast, electrochemical aptamer-based sensors are intrinsically compatible with conventional electrochemical circuits and have robust operation under physiological ionic conditions. Through optimized aptamer design and electrode surface engineering, femtomolar-level sensitivities have been demonstrated in recent studies. Despite these advances, most electrochemical aptamer sensors have been implemented as standalone sensing elements, with working electrode (WE), reference electrode (RE), and counter electrode (CE) spatially separated or externally referenced.^12,26,27,30^ Such configurations limit spatial precision, increase surgical complexity, and hinder seamless integration with electrophysiological recording architectures,^8,20,31^ thereby constraining their utility in multifunctional neural interfaces.

Taken together, these considerations motivate the development of electrochemical aptamer-based sensing architectures in which a complete three-electrode system is monolithically integrated within the same microscale neural interface used for electrophysiological recordings. In this work, a flexible microelectrode array is developed in which a fully on-chip three-electrode electrochemical system is cointegrated with a wideband electrophysiological recording interface within a single implantable platform. Chemical specificity is introduced through surface functionalization of the electrochemical WE with an aptamer-based molecular recognition layer, while electrophysiological signals are acquired through dedicated recording channels. The aptamer-modified sensing interface is designed to support limits of detection in the femtomolar (fM) range while maintaining selectivity against electroactive and biochemical interferents.

To validate this multimodal sensing strategy under physiologically relevant conditions, we employ acute pharmacological modulation to transiently elevate extracellular neurotransmitter levels *in vivo*. Nomifensine, a well-characterized monoamine reuptake inhibitor, was selected as a perturbation model because extracellular dopamine (DA) can be increased through a defined presynaptic mechanism without introducing electrical stimulation artifacts at the recording interface. When combined with electrophysiological recordings, this approach provides an orthogonal and temporally resolved reference for neural activity, enabling a correlation between neurochemical signals and circuit-level electrical dynamics.

## Results and Discussion

### Electrical and Electrochemical Characterization of the Multifunctional Microelectrode Array of Neural Probe

Prior to the *in vivo* experiments, the electrical properties of the electrophysiological recording microelectrodes were evaluated to verify their suitability for neural signal acquisition. Electrochemical impedance spectroscopy (EIS) measurements were performed using three independently fabricated neural probes, each integrating four electrophysiological recording microelectrodes (Figure 1a, red box). The corresponding impedance spectra and reproducibility analyses are presented in Figure S1 and Table S1 of **Supplementary Note 1**. The recording microelectrodes exhibited a mean impedance magnitude of 366.02 ± 18.63 kΩ (total *N* = 12 microelectrodes from three independently fabricated neural probes, each containing 4 independent microelectrodes) at 1 kHz, with a probe-to-probe coefficient of variation (CV%) of 5.09%, while the within-probe CV% ranged from 1.27 to 8.85%, demonstrating excellent fabrication reproducibility across independently fabricated neural probes as well as between recording microelectrodes integrated within the same probe.

**Figure 1:**
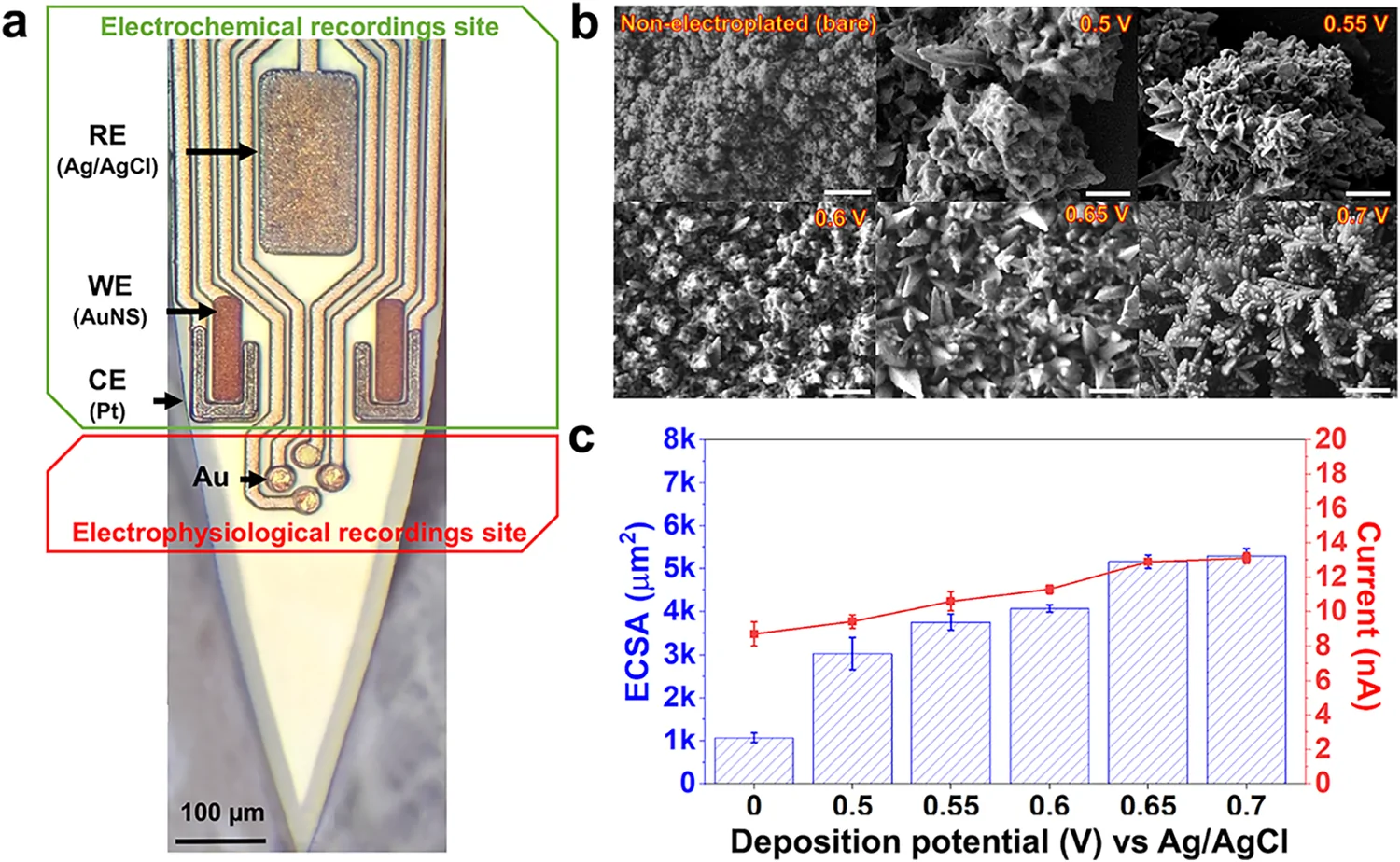
Structural and electrochemical characterization of the multifunctional microelectrode array. (a) Optical micrograph of the flexible neural probe showing spatially separated electrophysiological recording sites and electrochemical sensing sites configured as two on-chip three-electrode systems. (b) SEM images of bare working electrodes and electrodes after chronoamperometric electrodeposition of AuNS at 0.5, 0.55, 0.6, 0.65, and 0.7 V vs Ag/AgCl, revealing potential-dependent variations in nanostructure morphology and surface coverage. (c) ECSA of bare and AuNS-modified electrodes estimated from cyclic voltammograms method in 0.1 M H_2_SO_4_ (blue bars, left axis) and corresponding redox currents of MB-labeled aptamers immobilized on bare and AuNS-modified electrodes, obtained by SWV in the presence of 1 pM DA (red squares, right axis). ECSA increases with deposition potential up to 0.65 V, beyond which further enhancement is limited. Error bars represent mean ± SD (*N* = 6, three probes × two WEs per probe).

Importantly, the measured impedance at 1 kHz falls within the range commonly reported for microelectrodes used for extracellular electrophysiological recordings and is considered suitable for the simultaneous recording of local field potentials (LFPs) and multiunit activity (MUA), where electrode impedances on the order of several hundred kilohms are generally preferred to achieve an appropriate balance between thermal noise, signal amplitude, and spatial selectivity.^32^ These results confirm that the electrophysiological recording electrodes provide stable electrical characteristics suitable for subsequent *in vivo* neural recordings.

Electrochemical sensing sites on the same flexible probe were configured into two independent of-chip three-electrode systems (Figure 1a, green box), enabling localized electrochemical measurements without the need for externally implanted reference or counter electrodes. To enlarge the electrochemically active surface area (ECSA) of the WEs, Au nanostructures (AuNSs) were fabricated by chronoamperometric electrodeposition.^33^ Deposition potentials ranging from 0.50 to 0.70 V (vs Ag/AgCl) were systematically investigated using a fixed deposition time of 3 min. Representative scanning electron microscopy (SEM) images confirmed the successful formation of AuNSs on the WEs (Figure 1b). At lower deposition potentials (0.50–0.55 V), relatively large and loosely packed nanostructures were observed, whereas increasing the deposition potential to 0.60–0.65 V produced smaller and more densely distributed nanostructures with improved surface homogeneity, consistent with enhanced nucleation density and accelerated growth kinetics at higher overpotentials. Dense AuNSs were also obtained at 0.70 V, indicating that increasing the electrodeposition potential effectively promoted Au nanostructure formation over the investigated potential range.

The influence of AuNS electrodeposition on the electrochemical properties of the WEs was evaluated by cyclic voltammetry in 0.1 M H_2_SO_4_. Mean cyclic voltammograms obtained from three independently fabricated neural probes, each containing two independent WEs (total *N* = 6 WEs from three independently fabricated neural probes, each comprising two separate three-electrode systems), are presented in Figure S2 (**Supplementary Note 2** *)*, while the corresponding ECSA values, together with the probe-to-probe reproducibility analyses, are summarized in Table S2 (**Supplementary Note 2** *)*. As shown in Figure 1c, AuNS electrodeposition substantially increased the ECSA from approximately 1.12 × 10^3^ μm^2^ for the nonelectrodeposited WE to 5.25 × 10^3^ μm^2^ at 0.65 V, corresponding to an approximately 4.7-fold enhancement. Increasing the deposition potential to 0.70 V produced only a marginal additional increase in ECSA (5.39 × 10^3^ μm^2^), suggesting that the accessible electrochemical surface area approached saturation under the present electrodeposition conditions. Furthermore, the relatively low probe-to-probe CV% observed across all deposition conditions demonstrates that the chronoamperometric electrodeposition process produced highly reproducible electrochemical enhancement across independently fabricated neural probes.

To determine whether the observed ECSA enhancement originated from genuine structural evolution rather than electrochemical measurement variability, quantitative atomic force microscopy (AFM) characterization was performed on independently fabricated WEs prepared under each electrodeposition condition. Representative AFM topographic images and corresponding surface profiles are presented in Figure S3 (***Supplementary Note 2***), while the arithmetic surface roughness (S_a_) values, together with the corresponding probe-to-probe CV%, are summarized in Table S3 (***Supplementary Note 2***). Consistent with the SEM observations (Figure 1b), the AFM images reveal progressively rougher AuNS morphologies as the electrodeposition potential increased from 0.50 to 0.70 V. This morphological evolution is quantitatively reflected by a systematic increase in the mean S_a_, which increased from 0.176 ± 0.012 μm for the nonelectrodeposited control to 0.524 ± 0.082 μm after electrodeposition at 0.70 V. This enhancement can be attributed to the increased nucleation density and growth of AuNS at higher electrodeposition potentials, which generate a highly roughened and porous surface topology, thereby substantially enlarging the effective electrochemical interface.^34^ Importantly, the relatively low probe-to-probe CV% obtained from six independently fabricated WEs (three independently fabricated neural probes, each containing two independent WEs) demonstrates excellent reproducibility of the electrodeposition process across independently fabricated devices. These quantitative AFM results therefore confirm that the structural evolution of the AuNS layer is both systematic and highly reproducible, providing an independent structural validation of the observed electrochemical enhancement.

To directly correlate the structural and electrochemical characterizations, the relationship between ECSA and AFM-derived S_a_ is presented in Figure S4 (***Supporting Information Note 2***). The progressive increase in ECSA closely followed the increase in surface roughness across the different electrodeposition conditions, demonstrating excellent agreement between the electrochemical measurements and the independently obtained AFM characterization. These complementary results confirm that the increase in ECSA originates from the systematic evolution of the AuNS surface morphology rather than experimental measurement variability, thereby providing quantitative structural evidence supporting the electrochemical characterization.

Although electrodeposition at 0.70 V yielded the largest average ECSA, the improvement over that at 0.65 V was relatively small. More importantly, optical microscopy revealed that electrodeposition at 0.70 V frequently produced excessive lateral growth of the AuNS layer beyond the designed WE boundary (Figure S5, ***Supporting Information Note 2***), thereby increasing the probability of electrical short circuits between adjacent electrodes within the densely integrated microelectrode array. In contrast, electrodeposition at 0.65 V produced well-confined AuNS growth while maintaining an ECSA comparable to that obtained at 0.70 V. Considering the combined electrochemical performance, quantitative structural characterization, probe-to-probe reproducibility, and fabrication robustness, 0.65 V was selected as the optimal electrodeposition potential for the fabrication of the AuNS-modified WEs and was therefore adopted for all subsequent electrochemical sensing experiments.

To further verify the successful fabrication of the integrated on-chip three-electrode system, the Pt-coated CEs and Ag/AgCl RE were additionally characterized by field-emission scanning electron microscopy (FE-SEM) and energy-dispersive X-ray spectroscopy (EDS) (Figures S7 and S8, ***Supporting Information Note 3***). The FE-SEM images confirmed uniform surface modification of the electrodeposited Pt and Ag/AgCl layers, while the corresponding elemental maps demonstrated the expected Pt, Ag, and Cl distributions without detectable cross contamination between adjacent electrodes, thereby confirming successful electrode-specific surface modification of the integrated WE, CE, and RE. Furthermore, the short-term electrochemical stability of the integrated on-chip Ag/AgCl RE was independently validated by open-circuit potential (OCP) measurements under physiologically relevant conditions. The corresponding experimental procedures and detailed analyses are presented in ***Supporting Information Note 4*** (Figures S9 and S10). Therefore, these structural, compositional, and electrochemical characterizations verify the successful fabrication and stable operation of the integrated on-chip three-electrode architecture prior to subsequent aptamer functionalization and electrochemical-sensing experiments.

In this study, a methylene blue (MB)-labeled DA-binding aptamer was subsequently self-assembled onto the optimized AuNS-modified WE through thiol-gold (Au–S) interactions to establish a molecularly selective sensing interface. From a molecular sensing perspective, enlargement of the ECSA increases the available surface for aptamer immobilization and thereby increases the population of electroactive MB reporters participating in electron transfer.^35^ Consistent with this expectation, WEs with progressively larger ECSA exhibited correspondingly higher MB oxidation currents (Figure 1c, red trace), reflecting both increased aptamer loading and enhanced interfacial electron-transfer efficiency. Therefore, these results demonstrate that AuNS modification not only enlarges the electrochemically active interface with the high conductivity and nanoscale curvature but also promotes faster interfacial electron-transfer kinetics and improved signal-to-noise ratio.^36^ Such properties are particularly advantageous for microscale electrodes intended for neurochemical detection at femtomolar concentrations, where efficient molecular capture and high-fidelity electrochemical transduction are essential for a reliable signal readout. However, electrodeposition at 0.70 V did not produce a further increase in the MB current, consistent with the relatively small additional increase in ECSA together with the onset of excessive lateral AuNS overgrowth (Figure S5, ***Supporting Information Note 2***). These results further confirm that electrodeposition at 0.65 V provides the most favorable sensing interface by maximizing the functional electrochemical response while maintaining a robust and spatially confined electrode fabrication.

Because the electrochemical response of MB-based electrochemical aptamer sensors depends not only on the sensing interface but also on the electrochemical interrogation conditions, the SWV operating parameters used for subsequent electrochemical measurements were evaluated next using the optimized 0.65 V AuNS-modified sensing interface. The SWV interrogation parameters employed in this study were initially selected according to operating conditions widely adopted for MB-based electrochemical aptamer sensors, in which interrogation frequencies ranging from several tens to a few hundred hertz, together with an amplitude of approximately 25 mV, have been extensively reported for electrochemical readout.^37–39^ Previous studies have demonstrated that the apparent optimal SWV interrogation frequency is governed primarily by the electron-transfer kinetics of the MB redox reporter rather than by a universal instrumental parameter.^40^ These electron-transfer kinetics are strongly influenced by the physicochemical characteristics of the sensing interface, including the electrode nanostructure, ECSA, aptamer packing density, and the mixed self-assembled monolayer formed following 6-mercapto-1-hexanol (6-MCH) backfilling.^38–40^

Accordingly, although the SWV amplitude (25 mV) and step potential (5 mV) were adopted from operating conditions widely reported for MB-based electrochemical aptamer sensors,^38,40,41^ the SWV interrogation frequency was experimentally evaluated over the range of 10–200 Hz for the present sensing interface. The complete frequency-validation results are provided in ***Supplementary Note 5*** (Figures S11 and S12). Increasing the interrogation frequency progressively enhanced both the absolute faradaic current and the corresponding background current, resulting in frequency-dependent changes in analytical performance. When the analytical response was normalized to the corresponding background current, the largest normalized signal enhancement was obtained at 100 Hz (Supplementary Figure S12), indicating the most favorable balance among signal generation, background contribution, and measurement reproducibility. This behavior is consistent with the electrochemical characteristics of MB-based electrochemical aptamer sensors, in which increasing the interrogation frequency enhances both faradaic and nonfaradaic current components, whereas excessively high interrogation frequencies progressively increase the background contribution and reduce the relative analytical response.^39,40^ Consequently, the experimentally identified optimum reflects the electron-transfer characteristics of the present sensing interface rather than a universal instrumental setting.^37,38,42^ Based on these results, 100 Hz was selected as the SWV interrogation frequency for all subsequent electrochemical measurements, including sequence-specificity validation, calibration, selectivity evaluation, and biological experiments.

To further verify that the electrochemical response originated from sequence-specific DA recognition rather than reversible nonspecific adsorption at the sensing interface, a composition-matched scrambled aptamer was introduced as a negative control. The scrambled probe retained the same sequence length, nucleotide composition, GC content, thiol anchor, internal MB labeling position, immobilization chemistry, and the 6-MCH backfilling procedure as the functional DA aptamer, while the DA-recognition sequence was deliberately disrupted (Table S4, ***Supporting Information Note 6***). Both sensing interfaces were interrogated by using the experimentally validated SWV conditions described above. As shown in Figure S13, exposure to 1 nM DA produced a pronounced increase in the MB oxidation current for the functional DA aptamer, whereas only a negligible current variation was observed for the scrambled aptamer under otherwise identical experimental conditions. Quantitative analyses further demonstrated excellent reproducibility for both sensing interfaces, with the corresponding within-probe and probe-to-probe reproducibility summarized in Tables S5 and S6 (***Supporting Information Note 6***). Therefore, these results demonstrate that the observed electrochemical response is predominantly governed by sequence-specific aptamer-DA recognition rather than reversible nonspecific DA adsorption or other interfacial artifacts.^43^

### Surface Chemical and Electrochemical Characterization of Aptamer Immobilization on AuNS-Modified WE

Following the identification of 0.65 V as the optimal electrodeposition potential for generating AuNS-modified WEs with large and reproducible ECSAs and enhanced MB redox responses, subsequent characterization focused on verifying DA aptamer immobilization and interfacial assembly on these optimized electrodes. Surface-sensitive X-ray photoelectron spectroscopy (XPS) and EIS were employed as complementary techniques to probe elemental composition, chemical bonding, and interfacial charge transfer properties associated with aptamer functionalization.

Survey XPS spectra revealed a decrease in the relative Au atomic percentage after aptamer modification compared with the AuNS-modified WE prior to functionalization (Figures S14a and S14b in ***Supplementary Note*** **7**), consistent with the formation of an organic overlayer covering the gold surface. Similar attenuation of Au signals has been widely reported for thiolated DNA or aptamer self-assembled monolayers on gold electrodes and is commonly attributed to partial masking of the underlying metal by the nucleic-acid film.^44–46^ High-resolution *Au* 4*f* spectra further showed the emergence of an additional component centered at ∼87.4 eV following aptamer immobilization (Figures S14c and S14d in ***Supplementary Note 7***), which is characteristic of Au–S bonding and indicates covalent attachment of thiolated aptamers to the gold surface via sulfur–gold interactions.^45,47^

Additional evidence for aptamer immobilization was obtained from analysis of the *C* 1*s* region. Compared with the AuNS-modified WE before functionalization (Figure S15a), the aptamer-modified WE exhibited an increased contribution from C–O bonding at ∼285.34 eV (Figure S15b), consistent with the presence of nucleic-acid backbones and associated oxygen-containing functional groups on the electrode surface.^48,49^ The immobilization of thiolated DA aptamers onto AuNS-modified WEs was further validated by XPS analysis, as evidenced by the appearance of Au–S covalent bonding features in the *Au* 4*f* spectra together with the enhanced contribution of carbon–oxygen bonding in the *C* 1*s* region, confirming the formation of a stable sulfur–gold linkage.^8,48^

The stepwise assembly of the aptasensor was characterized by EIS, with the corresponding Nyquist plots and the fitted equivalent circuit model shown in Figure 2. EIS provided independent electrochemical evidence of each interfacial modification step. Impedance spectra were recorded in 5 mM [Fe (CN)_6_]^3–/4–^ solution after AuNS electrodeposition, after aptamer immobilization, and after subsequent backfilling with 6-MCH, as shown in Figure 2a. The impedance spectra were well described by a modified Randles-type equivalent circuit, *R*_*S*_ – (*CPE*∥(*R*_*ct*_ + *Z*_*w*_)), as shown in Figure 2b, consisting of the solution resistance (*R*_*s*_), constant phase element (*CPE*), charge-transfer resistance (*R*_*ct*_), and Warburg impedance element (*Z*_*w*_). This equivalent circuit accurately captured both the nonideal capacitive behavior of the nanostructured interface and the diffusion-controlled transport of the redox probe.^50^ The impedance spectra presented in Figure 2b and the corresponding fitted parameters summarized in Table 1 were extracted from the same data set comprising three independently fabricated neural probes (Probe #1–#3), each integrating two independent electrochemical sensing units (total *N* = 6). To further strengthen the quantitative assessment of fabrication reproducibility, the corresponding CV% for *R*_*ct*_, *Q*, and *n* have been incorporated into Table 1. These additional statistics provide a complementary evaluation of the reproducibility of the stepwise surface functionalization process, including the bare AuNS surface, aptamer immobilization, and subsequent 6-MCH backfilling across independently fabricated neural probes. In addition, the within-probe reproducibility of the fitted electrochemical parameters is summarized in Table S6 (***Supplementary Note 8***), further demonstrating that the two independently fabricated sensing units integrated within each neural probe exhibit highly consistent interfacial electrochemical characteristics following surface functionalization.

**Figure 2:**
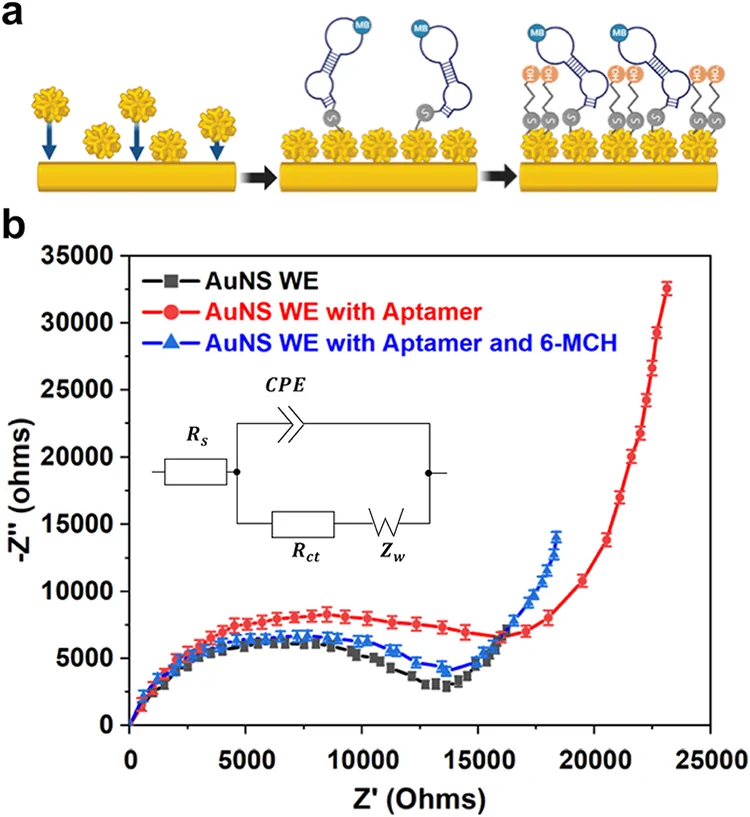
Stepwise interfacial assembly and impedance characterization of the electrochemical aptamer-based sensing WE. (a) Schematic illustration of the sequential surface functionalization of the AuNS-modified WE. First, gold nanostructures were electrodeposited onto the WE via chronoamperometry to generate a high-surface-area conductive nanoelectrode. Subsequently, the thiolated MB-labeled DA aptamer was immobilized onto the AuNS surface through spontaneous Au–S self-assembly, forming a surface-confined molecular recognition layer. Finally, 6-MCH was introduced as a short-chain alkanethiol spacer to passivate unoccupied gold sites and induce structural reorganization of the aptamer layer into a more ordered and upright configuration. (b) Nyquist plots recorded in 5 mM [Fe­(CN)_6_]^3–/4–^ containing 0.1 M KCl after AuNS electrodeposition (AuNS WE), after aptamer immobilization (AuNS WE with aptamer), and after subsequent 6-MCH backfilling (AuNS WE with aptamer and 6-MCH). The impedance spectra were fitted using a modified Randles equivalent circuit model *R*_*S*_ – *(CPE* ∥ (*R*_*ct*_ + *Zw*)). Measurements were performed using independently fabricated working electrodes (*N* = 6; three probes × two WEs per probe), and the extracted electrochemical parameters are presented as mean ± SD. The increase in the high-frequency semicircle following aptamer immobilization indicates an increase in *R*_*ct*_ due to the formation of the surface-confined nucleic acid layer. Subsequent 6-MCH treatment reduced the semicircle diameter, suggesting reorganization of the interfacial molecular architecture into a more ordered mixed monolayer while maintaining electrochemical accessibility to the redox probe. Inset: equivalent circuit model used for impedance fitting.

**Table 1:** Extracted EIS Fitting Parameters (*R*_*ct*_ *Q*, and *n*) for Electrodes at Different Modification Stages Based on the Equivalent Circuit Model *R*_*S*_ – *(CPE* ∥ (*R*_*ct*_ + *Z*_*w*_))^a^,^b^

	fitting parameter					
modification step	*R*_*ct*_(Ω)	interprobe CV%	*Q* (S·s^n^)	interprobe CV%	*n* (nonideality)	interprobe CV%
Bare AuNS surface	3255 ± 241	7.40	(4.8 ± 0.2) × 10^–7^	4.17	0.88 ± 0.03	3.41
Aptamer	8179 ± 319	3.90	(3.1 ± 0.1) × 10^–7^	3.23	0.82 ± 0.02	2.44
Aptamer +6-MCH	4531 ± 318	7.02	(2.4 ± 0.2) × 10^–7^	8.33	0.85 ± 0.02	2.35

a The fitted parameters were obtained for the bare AuNS surface, the aptamer-functionalized AuNS surface, and the AuNS surface after subsequent 6-MCH backfilling. Data are reported as mean ± SD together with the corresponding interprobe CV%, calculated from six independently fabricated WEs (three independently fabricated neural probes, each comprising two independent electrochemical sensing units; total *N* = 6). The interprobe CV% values quantitatively evaluate the reproducibility of the stepwise interfacial modification process across independently fabricated sensing WEs.

b Note: Data represent mean ± SD (total *N* = 6 WEs from three independently fabricated neural probes, each comprising two separate three-electrode systems [two WEs per probe]).

In the Nyquist representation (Figure 2b), the diameter of the high-frequency semicircle corresponds to *R*_*ct*_, which reflects the interfacial electron-transfer kinetics between the [Fe (CN)_6_]^3–/4–^ redox couple and the electrode surface. The AuNS-modified WE exhibits a relatively low *R*_*ct*_ (∼3,255 Ω), indicating efficient electron-transfer pathways enabled by the enlarged ECSA and high conductivity of the nanostructured gold layer. Following aptamer immobilization, *R*_*ct*_ increases markedly to ∼8,179 Ω, as evidenced by the pronounced enlargement of the semicircle. This increase originates from the formation of a surface-confined nucleic acid layer, which alters the interfacial electron-transfer mechanism. The negatively charged phosphate backbone introduces electrostatic repulsion toward the [Fe (CN)_6_]^3–/4–^ probe, while the molecular film increases the tunneling distance and imposes steric hindrance. As a result, the electron-transfer process transitions from a surface-controlled regime to a barrier-limited transport process, leading to a substantial increase in *R*_*ct*_.^51,52^

Upon subsequent backfilling with 6-MCH, *R*_*ct*_ decreases to ∼4,531 Ω, accompanied by a notable change in the Nyquist arc profile. This reduction does not indicate a loss of surface coverage or an ineffective passivation layer, but instead reflects a systematic reorganization of the interfacial molecular architecture. Short-chain alkanethiols displace loosely bound, nonspecifically adsorbed nucleic acid segments and occupy vacant gold sites, thereby promoting a transition from a highly disordered, entangled molecular state to a structurally optimized and spatially regulated mixed monolayer. This structural rearrangement reduces intermolecular entanglement, alleviates lateral electrostatic crowding, and eliminates nonproductive steric constraints, consequently creating more defined ion-transport pathways across the interface.^44,53^ As a result, the effective electron-transfer pathway becomes shorter and more homogeneous, yielding a lower apparent *R*_*ct*_ while strictly preserving and structurally stabilizing the density of functional recognition elements.^54^

In addition to the resistive component, the capacitive behavior of the interface was analyzed using a constant phase element (*CPE*), as incorporated in the equivalent circuit model *R*_*S*_ – (*CPE* ∥ (*R*_*ct*_ + *Z*_*w*_)). The use of a *CPE* rather than an ideal capacitor is physically justified by the structural and compositional heterogeneity of the interface, arising from the nanoscale roughness of the AuNS layer and the nonuniform molecular organization introduced during aptamer immobilization and 6-MCH passivation.^55^ The extracted *CPE* parameters (*Q* and *n*) are summarized in Table 1. The *CPE* magnitude (*Q*), which reflects the effective interfacial capacitance, decreases progressively from ∼4.8 × 10^–7^ S*·*s^*n*^ for the bare AuNS surface to ∼3.1 × 10^–7^ S*·*s^*n*^ after aptamer immobilization, and further to ∼2.4 × 10^–7^ S*·*s^*n*^ following 6-MCH treatment. This monotonic decrease is consistent with the formation of insulating molecular layers that increase the effective dielectric thickness and reduce charge storage capacity at the interface.

The evolution of the nonideality factor (*n*) further supports this interpretation. The decrease in *n* from ∼0.88 (bare AuNS) to ∼0.82 after aptamer immobilization indicates increased interfacial heterogeneity and distributed relaxation behavior associated with the flexible, nonuniformly distributed biomolecular layer. After 6-MCH passivation, *n* partially recovers to ∼0.85, suggesting the formation of a more compact, structurally uniform, and better-organized mixed monolayer. Such behavior is consistent with established electrochemical interpretations of *CPE* behavior, where deviations from ideal capacitive response arise from surface roughness, interfacial heterogeneity, and distributed relaxation processes.^56^

The evolution of these EIS parameters suggests that the electrochemical response following 6-MCH treatment cannot be interpreted solely within the conventional insulating-layer model of self-assembled monolayers (SAMs). In an ideal, homogeneous SAM composed of simple alkanethiols, the incorporation of an additional molecular layer is universally expected to increase the electron-transfer barrier and, consequently, increase the *R*_*ct*_. However, DNA- and aptamer-modified electrodes constitute structurally heterogeneous mixed monolayers, in which electron-transfer kinetics are governed not only by macroscopic molecular coverage but also by a complex interplay of probe density, spatial organization, interfacial heterogeneity, and local electrostatic environments. Therefore, the electrochemical response following 6-MCH backfilling reflects the combined effects of surface passivation and nanoscale molecular reorganization rather than a primitive increase in insulating thickness.^57–59^

Previous studies have consistently demonstrated that thiolated nucleic acid probes immobilized on gold surfaces rarely form an ideal, uniformly organized monolayer immediately upon incubation. Instead, nucleobases frequently engage in nonspecific adduction with the gold substrate, leading to highly heterogeneous probe distributions, partially collapsed conformations, and localized probe overcrowding.^57^ During the backfilling process, 6-MCH actively occupies uncoordinated Au sites while competitively displacing these loosely adsorbed segments through stronger, preferential Au–S coordination. This process eliminates nonspecific surface interactions, optimizes intermolecular spacing, and promotes the formation of a more homogeneous aptamer/6-MCH mixed monolayer.^59^ Recent single-molecule AFM studies and advanced surface characterizations were summarized by Gu et al. (2023),^58^ confirming that nucleic acid monolayers are intrinsically heterogeneous at the nanoscale and that small-molecule spacer thiols play an indispensable role in regulating intermolecular organization, mitigating structural clustering, and establishing uniform electrochemical DNA interfaces.

The EIS fitting parameters obtained in the present study provide independent, quantitative experimental evidence supporting this interfacial reorganization mechanism. Following aptamer immobilization, the marked increase in *R*_*ct*_ to 8179 ± 319 Ω confirms the initial formation of a blocking biomolecular film, while the concurrent decrease in the *CPE* exponent *n* from 0.88 to 0.82 mathematically captures the enhanced interfacial heterogeneity and broader distribution of relaxation times characteristic of nonideal electrochemical interfaces.^60^ Upon subsequent 6-MCH treatment, the *CPE* magnitude *Q* further decreases from (3.1 ± 0.1) × 10^–7^ to (2.4 ± 0.2) × 10^–7^ S*·*s^*n*^. Because *Q* is inversely related to the effective dielectric thickness of the interface,^61^ its progressive decline confirms the successful incorporation of the alkanethiol molecules and enhanced surface passivation.^62^ Crucially, however, *n* simultaneously undergoes a distinct recovery from 0.82 to 0.85. This unequivocal reduction in interfacial heterogeneity denotes a transition toward a highly ordered, structurally uniform mixed monolayer. The divergent trajectories of *Q* (reflecting passivation thickness) and *n* (reflecting structural homogeneity) demonstrate that 6-MCH simultaneously fulfills two distinct physicochemical roles: enhancing surface passivation while optimizing interfacial molecular organization.

Under these conditions, the substantial decrease in *R*_*ct*_ must be interpreted through the lens of this architectural optimization rather than as a contradiction to passivation. Although the incorporation of 6-MCH increases the physical thickness of the interface, the accompanying improvement in molecular organization reduces localized probe overcrowding, minimizes nonproductive steric constraints, and alleviates local electrostatic repulsion between the highly negative phosphate backbone and the [Fe (CN)_6_]^3–/4–^ redox probe. These structural modifications facilitate the efficient diffusion of the redox probe through a matrix of uniformly distributed, electrochemically accessible regions, effectively outbalancing the increase in dielectric thickness introduced by the alkanethiol layer. This unique electrochemical behavior is deeply supported by historical precedents in DNA/MCH mixed-monolayer systems, where interfacial organization and charge density, rather than molecular thickness alone, dominate the observed electron-transfer characteristics.^57–59,63^

Importantly, the evolution of both resistive (*R*_*ct*_) and capacitive (*Q*, *n*) parameters follows a consistent and complementary trend across modification steps.^64^ While *R*_*ct*_ captures dynamic changes in electron-transfer kinetics, the concurrent decrease in *Q* provides independent evidence of increased dielectric thickness and molecular coverage.^61^ Together, these parameters establish a coherent physicochemical picture of interfacial evolution during stepwise surface functionalization, as summarized in Table 1. The consistency between these resistive and capacitive parameters further reduces the likelihood that the observed impedance changes arise from fitting artifacts or ambiguities in equivalent-circuit interpretation.^61,65^

This interpretation is further supported by the frequency-dependent behavior of the impedance spectra. At high frequencies, *Z*_*w*_ is negligible, and the response is dominated by the parallel *CPE*–*R*_*ct*_ branch, reflecting interfacial kinetics. At lower frequencies, the Nyquist plots exhibit a characteristic ∼45° diffusion tail, indicating that mass transport of the redox probe contributes to the overall impedance. The persistence of this diffusion-controlled region after each modification step confirms that the molecular layers remain permeable to small redox species and that the interface remains electrochemically accessible rather than fully blocking.^52^ The smoother, more distinct transition into the diffusion regime after 6-MCH treatment suggests a more uniform and well-defined interfacial structure, consistent with the formation of an electrochemically accessible and structurally optimized mixed monolayer. In this study, the combined evolution of *R*_*ct*_, *CPE* parameters, and Nyquist profiles reveals a clear transition from a nanostructure-enhanced conductive interface (AuNS WE), to a barrier-dominated molecular interface (aptamer-modified WE), and finally to an ordered and transport-efficient sensing architecture following 6-MCH backfilling. These results collectively confirm successful stepwise surface functionalization and the establishment of a structurally optimized and electrochemically accessible interface for subsequent DA detection.

### *In Vitro* Electrochemical Sensing Performance

The *in vitro* electrochemical sensing performance of the aptamer-modified neural probe was systematically evaluated by comparing a conventional three-electrode configuration to the fully integrated on-chip three-electrode system implemented on the neural probe. In the conventional configuration, the WE on the probe was operated together with an externally positioned commercial Ag/AgCl RE and platinum CE, as illustrated in the inset of Figure 3a. In contrast, the integrated architecture monolithically incorporates the WE, RE, and CE on the same probe substrate, thereby forming a localized on-chip three-electrode electrochemical system (the inset of Figure 3b).

**Figure 3:**
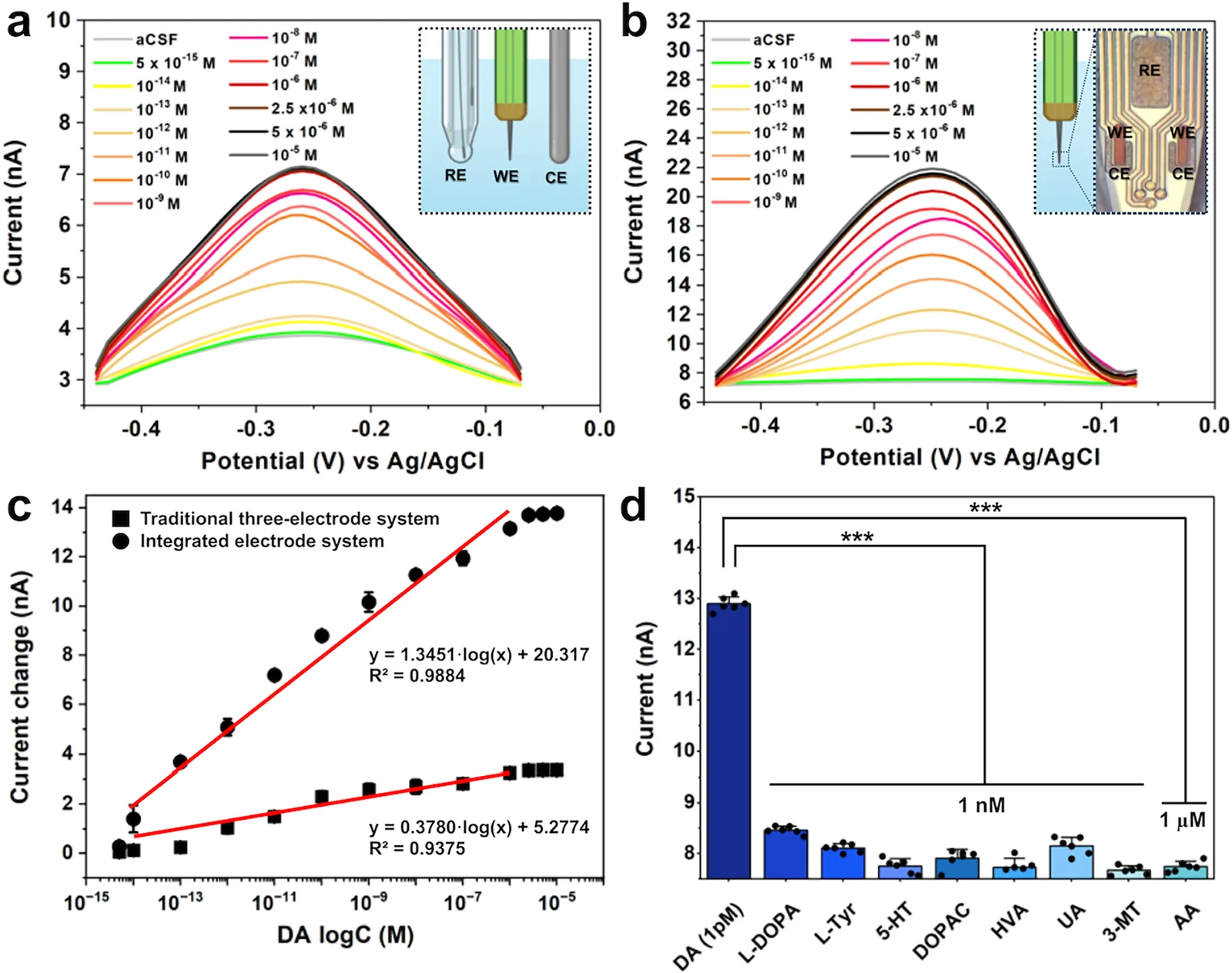
***In vitro*** electrochemical performance of the aptamer-functionalized microelectrode array for DA sensing. (a) Mean square-wave voltammetry (SWV) curves recorded in aCSF for DA concentrations from 5 fM to 10 μM using a conventional three-electrode configuration with physically separated WE, RE, and CE (inset). (b) Mean SWV curves obtained using the integrated on-chip three-electrode system of the neural probe, in which the WE, RE, and CE are monolithically integrated on the same probe tip (inset). Measurements were obtained from three independently fabricated neural probes, each containing two independent three-electrode sensing systems (total *N* = 6 sensing units [three probes × two WEs per probe]). For each sensing unit, electrochemical measurements at each concentration were repeated 10 times. (c) Calibration plots derived from the SWV peak current change (Δ*I*) as a function of logarithmic DA concentration for the conventional and integrated three-electrode systems. Measurements were obtained from three independently fabricated neural probes, each containing two independent three-electrode sensing systems (total *N* = 6 sensing units). For each sensing unit, electrochemical measurements at each concentration were repeated 10 times. Error bars represent mean ± SD. The experimentally validated LOD of the integrated on-chip system was determined to be 10 fM using the 3σ criterion. (d) Selectivity evaluation of the electrochemical aptamer sensor. Responses to 1 pM DA were compared with responses to 1 nM concentrations of L-DOPA, l-Tyr, 5-HT, DOPAC, HVA, UA, 3-MT, and AA. Data are presented as mean ± SD from three independently fabricated neural probes, each containing two independent three-electrode sensing systems (total *N* = 6, sensing units). ***p < 0.001.

SWV measurements were performed in aCSF across DA concentrations ranging from 5 fM to 10 μM. For both electrochemical configurations, well-defined MB redox peaks were observed, and the peak currents increased progressively with increasing DA concentration (Figure 3a,b). Near the experimentally determined detection limit, the integrated on-chip three-electrode system exhibited greater signal distinguishability from the blank distribution than the conventional three-electrode configuration. The blank signal distribution was determined from repeated DA-free aCSF measurements obtained from *N* = 6 independent sensing units, yielding a standard deviation (SD, σ_*blank*_) of 0.2460 nA (Table S8, ***Supplementary Note 9***). Based on the conventional criterion of three times the blank-signal SD (3×σ_*blank*_),^66^ the detection threshold was defined as 0.738 nA above the blank response. Under these conditions, the response obtained at 5 fM remained within the variation of the blank distribution, whereas the mean response at 10 fM exceeded the experimentally determined 3 × σ_*blank*_ threshold and was statistically distinguishable from the blank signal. Accordingly, 10 fM was determined to be the lowest experimentally distinguishable DA concentration under the present measurement conditions and therefore represents the experimentally determined limit of detection of the integrated on-chip three-electrode system. Although increased signal variability was observed at this concentration, as expected for measurements performed near the detection limit, the response satisfied the analytical criterion for experimental detectability. Quantitative measurement precision at concentrations approaching the detection limit is discussed separately from the determination of the limit of detection (LOD).

To further validate the sensing mechanism and assess the response behavior at higher DA concentrations, the measurements were performed at elevated DA concentrations (2.5 μM, 5 μM, and 10 μM). As shown in Figure 3a,b, the signal increase became progressively smaller at higher DA concentrations, approaching a plateau-like response behavior. This saturation tendency was particularly evident above the micromolar regime, where the incremental signal enhancement between 5 μM and 10 μM DA became minimal. Such saturation behavior is characteristic of a finite binding-limited sensing process and is consistent with specific aptamer-DA interactions. In contrast, nonspecific adsorption would typically produce continuously increasing signals without a defined plateau. These results therefore support that the observed electrochemical responses originate predominantly from specific aptamer-mediated DA recognition rather than nonspecific surface accumulation.

Quantitative calibration analysis based on the change in oxidation peak current (Δ*I*) is summarized in Figure 3c. Both electrochemical configurations exhibited concentration-dependent responses over the investigated DA concentration range from 10 fM to 1 μM that were well described by a semilogarithmic calibration model. Within this broader detectable concentration range, the integrated on-chip three-electrode system exhibited improved calibration fitting (*R* ^2^ = 0.9884) compared with the conventional three-electrode configuration (*R* ^2^ = 0.9375), together with a substantially steeper calibration slope. The integrated configuration exhibited a sensitivity of 1.3451 nA dec^–1^, which was approximately 3.6-fold higher than that of the conventional configuration (0.3780 nA dec^–1^). These results indicate that the integrated on-chip architecture provides superior analytical sensitivity while extending the detectable concentration range toward lower DA concentrations. This enhanced analytical performance is attributed to the colocalized WE, RE, and CE architecture, which minimizes interelectrode spacing, reduces uncompensated solution resistance, and improves local electrochemical potential stability. These effects are particularly important in miniaturized electrochemical systems, where signal attenuation and electrical noise can significantly limit sensing performance.^67,68^

Because the integrated on-chip three-electrode system demonstrated superior analytical performance and a broader detectable concentration range than the conventional configuration, its quantitative operating characteristics were further examined using direct linear regression of the nontransformed concentration data (Figure S20, ***Supporting Information Note 9***). This analysis identified two local absolute linear response regions spanning 0.5 pM to 10 pM (*R* ^2^ = 0.9945) and 0.5 nM to 10 nM (*R* ^2^ = 0.9949), respectively. These concentration intervals represent the ranges over which the electrochemical response varies linearly with the absolute DA concentration and, therefore, define the quantitative linear operating regions of the integrated sensing platform.

Between these two linear regions (10 pM to 0.5 nM), the calibration response remained monotonic but no longer satisfied the statistical criteria required for direct linear regression using absolute DA concentration. Instead, the response gradually transitioned between the two quantitative linear regimes while remaining well described by the overall semilogarithmic calibration relationship shown in Figure 3c. Such behavior is consistent with the equilibrium-controlled conformational switching mechanism characteristic of electrochemical aptamer-based sensors,^69^ in which target binding progressively shifts the conformational equilibrium of surface-confined aptamers, resulting in a nonlinear calibration response over a broad concentration range rather than a single absolute linear dependence on analyte concentration.^70,71^ Accordingly, this intermediate concentration interval should be interpreted as a nonlinear transition region rather than an additional absolute linear analytical range.

The broader detectable concentration range from 10 fM to 1 μM was selected because it encompasses the physiologically relevant extracellular DA concentrations targeted in this study. Extracellular DA concentrations in the brain are typically reported within the nanomolar to submicromolar concentration range.^72^ Within this biologically relevant concentration window, the sensing interface operates in the dynamic presaturation region of the overall calibration profile, enabling sensitive quantification of DA fluctuations without substantial signal compression. Consistent with the additional high-concentration measurements, signal saturation became evident above approximately 5 μM, confirming the finite binding capacity of the aptamer-based sensing interface. Therefore, the absence of saturation within the physiological concentration range should not be interpreted as evidence of nonspecific binding.^73^

To comprehensively evaluate the reproducibility of the proposed sensing platform, both probe-to-probe reproducibility and within-probe reproducibility were analyzed using the calibration data sets summarized in ***Supplementary Note 9*** (Table S8). Probe-to-probe reproducibility was first evaluated by comparing the calibration responses obtained from three independently fabricated neural probes (Table S8). Across the entire DA concentration range, the integrated on-chip three-electrode system consistently exhibited lower probe-to-probe variability than the conventional three-electrode configuration. As expected, relatively large CV% values were observed at concentrations near the experimentally determined detection limit because the measured faradaic currents approached the background noise level. For the integrated configuration, the CV% decreased from 39.28% at 10 fM to 5.18% at 100 fM, and further decreased to below 7% throughout the picomolar concentration range, remaining below 4% from 100 pM to 10 μM. In contrast, the conventional three-electrode configuration exhibited substantially higher variability over the same low-concentration range, with CV% values of 112.50% at 10 fM, 60.19% at 100 fM, and 22.10% at 1 pM, demonstrating markedly poorer reproducibility near the detection limit. These results indicate that the integrated on-chip architecture provides substantially improved analytical reproducibility, particularly for low-concentration DA detection.

To further examine the reproducibility of the two independently fabricated sensing units integrated within each neural probe, the individual calibration results of every sensing unit are summarized in Supplementary Note 10. For the integrated on-chip three-electrode system as shown in Table S9, the two sensing units on the same probe exhibited highly consistent concentration-dependent responses throughout the investigated concentration range. Apart from the expected increase in variability near the detection limit, the within-probe CV% values remained below 5% for nearly all concentrations above 1 pM, and further decreased to approximately 1% or lower throughout the nanomolar and micromolar concentration ranges. Similar concentration-dependent reductions in variability were observed consistently across all three independently fabricated probes, indicating excellent fabrication uniformity between the two sensing units integrated on the same neural probe. In contrast, the conventional three-electrode configuration exhibited considerably larger within-probe variability at low DA concentrations (Table S10). For example, the within-probe CV% at 10 fM ranged from 91.70% to 183.89%, substantially exceeding that of the integrated on-chip configuration (14.57–26.54% across the corresponding sensing units). Although the variability of both configurations decreased as the DA concentration increased, the integrated on-chip system consistently maintained lower within-probe variation across the entire concentration range, demonstrating improved reproducibility not only between independently fabricated probes but also between sensing units fabricated on the same neural probe.

As expected, the relative variability increased at DA concentrations approaching the experimentally determined detection limit, where the measured faradaic currents became increasingly comparable to the instrumental noise floor. Consequently, larger CV% values were observed at femtomolar DA concentrations, particularly for the conventional three-electrode configuration, because of its lower signal-to-noise ratio. This increase in variability reflects the expected reduction in measurement precision near the detection limit rather than a loss of signal detectability. As the DA concentration increased, the variability decreased substantially, and the integrated on-chip three-electrode system consistently maintained lower probe-to-probe and within-probe variation than the conventional configuration.

The improved reproducibility and enhanced analytical performance observed for the integrated on-chip three-electrode system can be attributed to its integrated electrode architecture. Specifically, integrating the WE, RE, and CE onto a single neural probe minimizes the interelectrode distance, thereby amplifying the electrochemical response to DA while simultaneously lowering the effective noise floor. The superior performance of the integrated on-chip three-electrode system is further attributed to improved electric-field uniformity,^74,75^ enhanced mass transport at the sensing interface, and stabilization of the reference potential provided by the on-chip RE.^20,75,76^ Collectively, these factors promote more consistent redox kinetics and reduce measurement variability, particularly at concentrations approaching the experimentally determined detection limit, where the analytical performance is most susceptible to electrical noise. Consequently, compared with the conventional three-electrode configuration, the integrated on-chip architecture achieved improved calibration linearity, together with a substantially lower experimentally determined limit of detection. On the basis of these analytical advantages, the integrated on-chip three-electrode system was selected for all subsequent experiments, including selectivity evaluation, flow injection analysis, *ex vivo* brain-phantom studies, and *in vivo* animal experiments, to ensure consistent and optimized sensing performance across all experimental platforms.

The molecular selectivity of the electrochemical aptamer sensor integrated within the on-chip three-electrode neural probe was further evaluated using a broadened panel of physiologically relevant interfering species (Figure 3d). The response generated by 1 pM DA was compared against common electroactive molecules, DA-related metabolites, and structurally related compounds, including ascorbic acid (AA), uric acid (UA), l-tyrosine (l-Tyr), L-3,4-dihydroxyphenylalanine (L-DOPA), serotonin (5-HT), 3,4-dihydroxyphenylacetic acid (DOPAC), homovanillic acid (HVA), and 3-methoxytyramine (3-MT). Among these interferents, DOPAC, HVA, and 3-MT are major DA metabolites in the brain extracellular environment, whereas AA and UA are highly abundant electroactive species known to interfere with conventional electrochemical DA detection. To impose stringent challenge conditions, L-DOPA, l-Tyr, 5-HT, DOPAC, HVA, UA, and 3-MT were tested at concentrations 10^3^-fold higher than DA (1 nM), while AA was evaluated at 1 μM, corresponding to a 10^6^-fold excess relative to DA, reflecting the extreme concentration disparity reported in brain extracellular fluid. Despite these highly exaggerated interference conditions, none of the tested species produced a significant signal response compared with DA, and the DA-induced current response remained distinctly higher than all interferents (*p < 0.001), demonstrating excellent molecular selectivity of the sensing platform.

To further verify that the observed molecular selectivity was reproducible rather than attributable to a single sensing unit, the selectivity evaluation was independently performed using all six electrochemical sensing units integrated within three independently fabricated neural probes. The detailed results are summarized in ***Supporting Information Note 10*** (Table S11), which separately presents the within-probe reproducibility between the two independent sensing units integrated within each neural probe and the corresponding probe-to-probe reproducibility across the three independently fabricated neural probes.

The within-probe reproducibility analysis demonstrated highly consistent electrochemical responses between the two sensing units integrated on the same neural probe. For dopamine, the within-probe CV% ranged from 0.11 to 1.71%, while the responses to all tested interfering species remained similarly reproducible, with CV% values generally below 3% across all probes. Similar reproducibility was observed at the probe level, where the pooled responses from three independently fabricated neural probes yielded a probe-to-probe CV% of 1.16% for DA and 0.90–2.36% for all interfering species. These consistently low CV% values indicate that the selectivity characteristics of the integrated sensing platform are well preserved across independently fabricated sensing units and neural probes. Collectively, these reproducibility analyses demonstrate that the selectivity observed in Figure 3d reflects the intrinsic sensing characteristics of the integrated platform rather than variability arising from device fabrication or individual sensing units.

The excellent reproducibility observed across independently fabricated sensing units further suggests that the high selectivity of the integrated sensing platform is governed primarily by its molecular recognition mechanism rather than variations in the electrode fabrication or electrochemical response. The selectivity mechanism of the present platform fundamentally differs from that of conventional oxidation-based electrochemical DA sensors. Traditional electrochemical sensors primarily rely on direct oxidation currents and are therefore highly susceptible to electroactive interferents such as AA and UA.^77^ In contrast, the present system employs an aptamer-functionalized sensing interface, in which signal generation originates from target-specific binding-induced conformational switching rather than direct oxidation of DA.^78^ Upon DA binding, the aptamer undergoes a structural rearrangement that modulates the distance between the MB redox reporter and the electrode surface, thereby producing a measurable electrochemical signal.^27^ Because this mechanism is governed by molecular recognition and aptamer affinity, rather than nonspecific electrochemical oxidation, electroactive interferents that do not specifically bind to the aptamer fail to induce comparable conformational responses even at substantially higher concentrations.^79^ This molecular recognition mechanism is consistent with the highly reproducible selectivity responses observed across independently fabricated probes and sensing units, indicating that the measured electrochemical responses are primarily determined by specific aptamer-target interactions. These results demonstrate that the integrated aptamer-based neural probe maintains high selectivity under physiologically challenging interference conditions and supports its applicability for selective *in vivo* DA sensing.

To further evaluate the analytical performance of the proposed sensing platform against previously reported electrochemical aptamer-based DA sensors, representative studies^80^ are summarized in Table S12 (***Supporting Information Note 11***). To facilitate comparison on a consistent analytical basis, the reported sensing performance is presented according to the experimentally determined absolute linear response range whenever available, while the broader detectable concentration range is listed separately, because these two parameters represent different analytical characteristics. The absolute linear response range defines the concentration interval suitable for quantitative analysis, whereas the detectable concentration range describes the concentration interval over which concentration-dependent electrochemical responses can be resolved.

Within this analytical framework, our integrated on-chip three-electrode system was found to exhibit two experimentally identified absolute linear response regions spanning 0.5–10 pM and 0.5–10 nM, together with experimentally distinguishable electrochemical responses at 10 fM. Accordingly, the experimentally determined limit of detection and the absolute linear response range are presented as complementary analytical metrics rather than interchangeable measures of sensing performance. Although several aptamer-FET platforms have reported femtomolar-level detection, these systems were not included in Table S12 because their charge-based transduction mechanisms^23,81^ differ fundamentally from electrochemical current-based sensing and therefore do not permit direct comparison.

### Intermittent Electrochemical Monitoring and Brain-Phantom Validation

The intermittent electrochemical monitoring capability and operational stability of the integrated electrochemical aptamer sensor were first evaluated using a flow injection analysis (FIA) system designed to mimic dynamic DA fluctuations in brain extracellular fluid or cerebrospinal fluid (CSF) (Figure 4a). For each reported electrochemical data point, the MB redox current represents the average of three consecutive SWV scans, which was adopted to improve the measurement robustness while minimizing random electrochemical fluctuations. The corresponding raw scan-to-scan variability, quantified as the CV% calculated from these three consecutive SWV scans, is presented separately in Figure S21 (***Supporting Information Note 12***).

**Figure 4:**
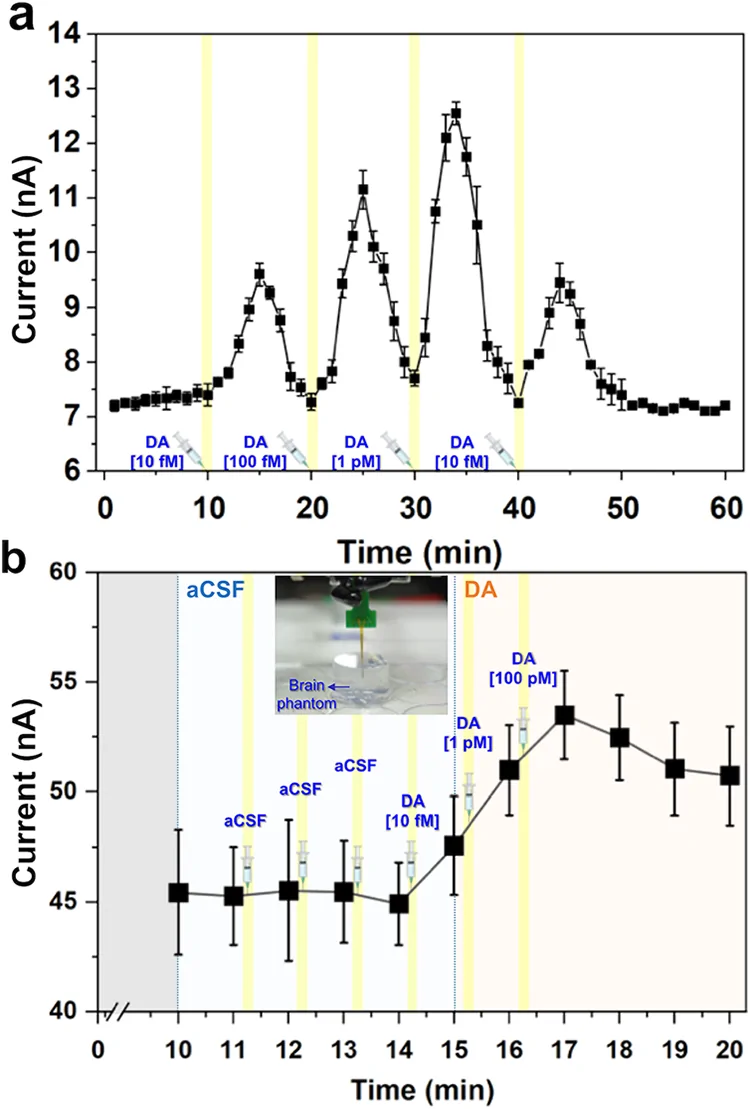
Time-resolved DA sensing using the integrated aptamer-based microelectrode array probe. (a) Dynamic electrochemical response of the on-chip neural probe under FIA. DA was sequentially introduced (10 fM, 100 fM, 1 pM, then back to 10 fM). Yellow regions indicate DA injection. Measurements were performed using SWV at 1 min intervals. Each data point represents the average of three consecutive scans per probe (one on-chip three-electrode system per probe), with *N* = 3 independent probes. Error bars denote SD (b) Electrochemical response in an *ex vivo* brain phantom (0.6% agarose in aCSF). After baseline stabilization with aCSF, DA (10 fM, 1 pM, 100 pM) was sequentially introduced near the implanted probe. Yellow shaded regions indicate DA addition periods. Measurements were performed under identical SWV conditions as in (a) at 1 min intervals. Each data point represents averaged responses from *N* = 3 independent probes. Error bars denote SD.

Sequential injections of DA (10 fM, 100 fM, and 1 pM) produced well-defined concentration-dependent increases in the average MB redox current. Upon decreasing the DA concentration from 1 pM back to 10 fM, the electrochemical signal returned to a level comparable to that obtained during the initial 10 fM injection, indicating reversible aptamer binding with minimal hysteresis and a negligible baseline drift. Analysis of the raw scan-to-scan measurements (Figure S21) further showed that transient increases in CV% occurred immediately following DA injection, reflecting dynamic concentration changes during solution exchange. As the DA concentration approached equilibrium, the scan-to-scan variability rapidly decreased and stabilized. Importantly, the scan-to-scan CV % remained below 10% throughout the experiment, demonstrating excellent short-term measurement reproducibility of the sensing unit. Therefore, these results confirm that the aptamer-functionalized AuNS-modified working electrode integrated with the on-chip three-electrode architecture provides stable, reversible, and reproducible time-resolved electrochemical monitoring of dynamic DA concentration changes under flow conditions.

To further approximate the microenvironment encountered during intracerebral implantation, a brain phantom composed of agarose gel infused with aCSF was prepared (Figure 4b, inset). The neural probe was inserted into the brain phantom to a depth of approximately 4.5 mm below the surface, matching the implantation depth used in the subsequent *in vivo* experiments. SWV measurements were performed using a frequency of 100 Hz, a step potential of 5 mV, an amplitude of 25 mV, and a potential window from 0 to −0.5 V. After probe insertion, the system was allowed to equilibrate for 10 min before electrochemical measurements. Thereafter, SWV acquisitions were performed at 1 min intervals, with each reported electrochemical data point representing the arithmetic mean of three consecutive SWV scans. The corresponding raw scan-to-scan variability, quantified as the CV% calculated from the three consecutive SWV scans, is presented separately in Figure S22 of ***Supplementary Note 12***.

During the initial phase, 1 μL aCSF was repeatedly injected adjacent to the sensing region to verify baseline stability. Subsequently, DA solutions (10 fM, 1 pM, and 100 pM) were sequentially introduced into the brain phantom, with each injection followed by a 1 min diffusion period before the next SWV acquisition. As shown in Figure 4b, the averaged MB redox current exhibited stepwise increases following each DA injection, and the signal magnitude increased with DA concentration over the tested range. Following cessation of DA injection, the electrochemical signal gradually decreased during subsequent measurements, consistent with DA diffusion away from the sensing interface.

Analysis of the raw scan-to-scan measurements (Figure S22) further demonstrated that the scan-to-scan CV % remained consistently low during repeated aCSF injections and exhibited only transient increases immediately after DA administration, reflecting the dynamic concentration changes occurring during DA diffusion within the brain phantom. As the local DA concentration approached equilibrium, the scan-to-scan variability progressively decreased and stabilized. Importantly, the scan-to-scan CV % remained below 10% throughout the entire experiment, demonstrating excellent short-term measurement reproducibility of the sensing unit under tissue-mimicking conditions. Therefore, these results demonstrate that the aptamer-functionalized AuNS-modified microelectrode maintains stable electrochemical performance within a tissue-mimicking matrix while providing reproducible, time-resolved monitoring of femtomolar to picomolar DA fluctuations. Building upon the validated performance demonstrated in both the FIA system and the brain phantom, the same sensing configuration was subsequently applied to evaluate the DA dynamics in the living brain.

### *In Vivo* Multimodal Characterization of Nomifensine-Induced Neurochemical and Electrophysiological Responses

Figure 5 presents *in vivo* multimodal recordings obtained from rats following intravenous administration of nomifensine using the integrated multimodal neural probe, which enables electrochemical and electrophysiological measurements in the caudate-putamen (CPu) complex of the rat brain. This platform allows concurrent monitoring of extracellular DA dynamics and local neuronal electrical activity, thereby providing a comprehensive assessment of drug-induced neuromodulatory effects in an intact brain.

**Figure 5:**
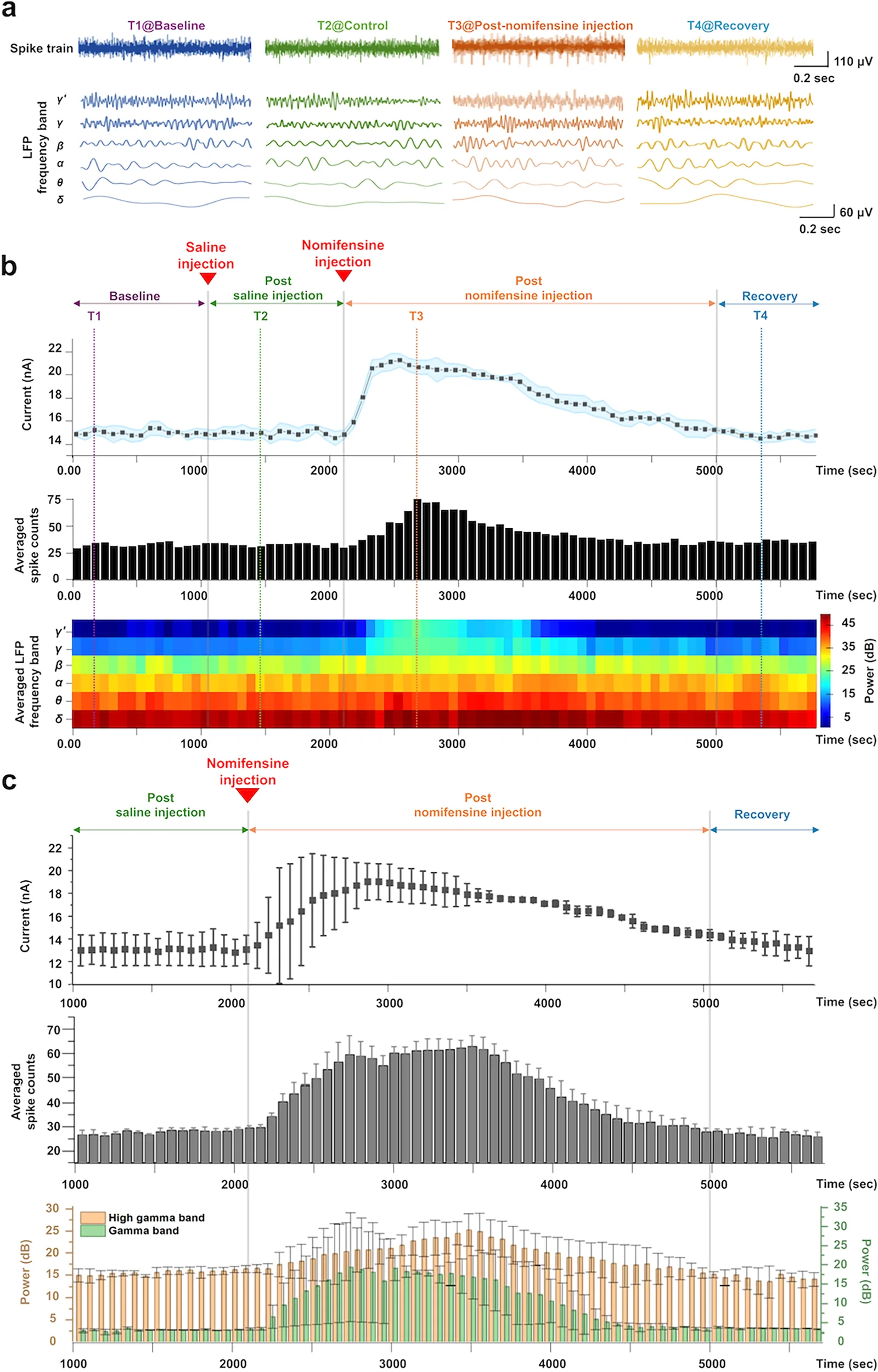
***In vivo*** electrochemical and electrophysiological characterization of nomifensine-induced neuromodulation using a multimodal neural probe. (a) Representative wideband neural recordings of MUA and decomposed LFPs (δ, θ, α, β, γ, and γ’) are shown at four time segments (T1–T4) selected to correspond to key experimental events. T1 (210–211 s) and T2 (1470–1471 s) were recorded before and after saline injection at 1,050 s, respectively, during which comparable MUA and LFP patterns were observed. T3 (2590–2591 s) was recorded following intravenous nomifensine administration at 2,100 s and captured a marked increase in MUA firing and γ/ γ’ LFP oscillatory activity. T4 (5250–5251 s) was acquired approximately 53 min later, when neural activity partially recovered toward baseline levels. (b) Temporal quantification of SWV current, spike counts, and band-specific LFP power across a representative recording session (*N* = 1 animal; error bars represent the SD of three consecutive SWV scans from the single probe). Each data point represents a 70 s measurement cycle, consisting of a 30 s electrochemical sensing phase, a 10 s idle interval, and a 30 s electrophysiological recording phase. Therefore, the consecutive SWV current data points were separated by 70 s. Spike counts of MUAs and LFP band power were also displayed over the full 70 s cycle duration, providing a continuous time-axis representation of the sequential multimodal measurements. The intravenous administration of nomifensine resulted in a pronounced increase in SWV peak current (from ∼14 to ∼22 μA), spike counts (from ∼25 to ∼100), and selectively enhances γ and γ’ band power, whereas saline injection produces no significant changes. The temporally correlated neurochemical and electrophysiological responses indicate tight coupling between extracellular DA elevation and neuronal excitability. (c) Reproducible *in vivo* neurochemical and electrophysiological responses to nomifensine-induced DA modulation measured across animals using the multimodal neural probe (*N* = 3 rats). Top panel: averaged SWV current responses measured by the electrochemical sensing system. Middle panel: averaged spike counts extracted from electrophysiological recordings. Bottom panel: averaged γ and γ’ LFP band power during the recording session. Saline injection produced minimal changes in both electrochemical and electrophysiological signals, whereas intravenous nomifensine administration induced gradual increases in DA current, spike activity, and γ/ γ’ LFP oscillatory power, followed by progressive recovery toward baseline levels. Despite moderate interanimal variability in peak amplitude and temporal kinetics, the overall response profiles remained consistent across animals. Gray vertical lines indicate the timing of saline injection, nomifensine injection, and the recovery phase. Error bars represent SD across animals.

Figure 5a shows representative wideband electrophysiological recordings at four distinct temporal epochs (T1–T4). The broadband signals were decomposed into multiunit activity (MUA) and local field potentials (LFPs), encompassing canonical frequency bands including δ, θ, α, β, γ, and high-γ (γ′). During the baseline period prior to saline injection (T1, 210–211 s) and after saline injection at 1050 s (T2), MUA firing patterns and LFP amplitudes exhibit highly comparable temporal structures and spectral characteristics, indicating that the injection procedure itself does not measurably perturb local neuronal activity.

In contrast, following intravenous nomifensine administration at 2,100 s (T3, 2590–2591 s), pronounced alterations in neural activity are observed. MUA displays a robust increase in firing density, accompanied by marked changes in LFP amplitude and temporal organization. Notably, LFP γ and γ’ band oscillations exhibit prominent and sustained enhancement, suggesting elevated neuronal synchrony and network excitability. At approximately 53 min postnomifensine (T4, 5250–5251 s), both MUA and LFP activity gradually return toward patterns resembling those observed during T1, indicating that the nomifensine-evoked electrophysiological effects are largely reversible.

To visualize the temporal coupling between neurochemical and electrophysiological signals, spike counts and LFP band power were calculated from each 30 s electrophysiological recording interval and displayed across the full 70 s measurement cycle duration (30 s electrochemical sensing, 10 s idle period, and 30 s neural recording). Although spike counts and power spectral density (PSD)-derived LFP band power were computed exclusively from the 30 s neural recording phase, each corresponding data bin was extended to span the entire 70 s cycle to maintain temporal continuity and facilitate alignment with sequential SWV measurements.

Figure 5b provides the quantitative time-course of the electrochemical and electrophysiological readouts. During baseline, SWV signals were stable at approximately 14 μA, accompanied by ∼25 spike events and low, steady band-specific LFP power, supporting the stability of the integrated probe for continuous *in vivo* recordings. After saline injection at 1,050 s, no appreciable changes were observed in SWV peak current, spike firing rate, or the LFP spectral power distribution, reinforcing that subsequent changes were not attributable to injection-related artifacts, systemic physiological fluctuations, or stress effects. After nomifensine injection at 2100 s, SWV peak current exhibited a rapid and substantial response, rising from ∼14 μA to a peak of ∼22 μA within ∼600 s and subsequently decaying toward baseline over ∼35–50 min. Given that nomifensine acts as a DA reuptake inhibitor,^82^ this SWV elevation is consistent with an increase in extracellular DA detected by the aptamer-functionalized microelectrode integrated on the probe.

Meanwhile, neuronal firing activity increased markedly, with spike counts rising from ∼25 to ∼100 events. The temporal evolution of firing activity broadly followed the rise-and-decay profile observed in the SWV response, indicating a qualitative correspondence between DA availability and local neuronal excitability.^31,83^ Time-frequency analysis further revealed a selective and significant elevation in LFP γ and γ’ band power following nomifensine administration,^31,84^ whereas lower-frequency bands exhibited comparatively modest changes. Such enhancement of high-frequency oscillations is widely associated with increased neuronal synchrony and DA-dependent modulation of local circuit dynamics. Therefore, the present results are intended to demonstrate the system-level capability of the platform to capture pharmacologically induced, multimodal neurophysiological changes *in vivo*, rather than to resolve fine-scale temporal relationships between DA fluctuations and neural activity. Within this framework, nomifensine administration acts as a well-established causal input that elevates extracellular DA and leads to measurable downstream modulation of neural activity, including changes in spike firing rates and LFP oscillatory patterns. Accordingly, the observed responses reflect coordinated neurochemical and electrophysiological changes at the network level, consistent with a pharmacologically driven modulation of striatal circuit activity.^85^

To further evaluate the robustness of *in vivo* neurochemical sensing, additional multianimal experiments were performed, and the averaged DA time-course responses from three independent animals (*N* = 3 rats) are summarized in Figure 5c. Across animals, intravenous nomifensine administration consistently induced gradual increases in the averaged DA-associated SWV current, spike activity, and γ and γ’ band power, followed by progressive recovery toward baseline levels. The corresponding individual DA time-course profiles are provided in Figure S23a of ***Supplementary Note 13***, in which each electrochemical data point represents the arithmetic mean of three consecutive SWV scans acquired at each measurement time point. Although all animals exhibited a consistent pharmacologically induced increase in extracellular DA following intravenous nomifensine administration, noticeable differences in the temporal response profiles were observed. In particular, one animal reached its peak DA response earlier than the other two animals, indicating intrinsic interanimal pharmacokinetic variability.

To distinguish the averaged electrochemical responses from the intrinsic measurement variability, the corresponding raw scan-to-scan reproducibility is presented in Figure S23b of ***Supporting Information Note 13***, where the CV% was calculated from the same three consecutive SWV scans underlying each averaged data point shown in Figure S23a. Importantly, all implanted neural probes maintained scan-to-scan CV% values below 10% throughout the entire recording period, demonstrating excellent intrameasurement reproducibility during dynamic *in vivo* neurochemical monitoring. Likewise, the scan-to-scan CV% at the peak response time point also remained below 10% for all animals (Table S13, ***Supporting Information Note 13***), indicating robust electrochemical measurement stability throughout the *in vivo* experiments.^86^

Quantitative analysis of the pharmacokinetic parameters (Table S14, ***Supporting Information Note 13***) further demonstrated that the interanimal CV% of the peak current was only 7.68%, indicating excellent reproducibility of the electrochemical sensing performance according to commonly accepted criteria. In contrast, the time-to-peak current exhibited a substantially larger interanimal CV % of 33.25%. Because the time-to-peak primarily reflects the systemic pharmacokinetic response to nomifensine rather than the intrinsic sensing capability of the neural probe in this study (see also Figure S23), this larger temporal variability is biologically expected. Individual differences in systemic drug absorption, blood–brain barrier transport, DA transporter inhibition kinetics, extracellular DA clearance, and endogenous DA release dynamics all contribute to the temporal evolution of localized DA signals,^82,87,88^ together with minor variations in neural probe implantation location and local electrode-tissue interactions, collectively influencing the temporal evolution of extracellular DA signals. Similar interanimal variability in DA response kinetics has been widely reported in previous *in vivo* studies following the pharmacological inhibition of DA reuptake. Despite differences in the exact timing of the peak response, all animals exhibited a highly consistent overall pharmacological pattern, characterized by a gradual rise in extracellular DA following nomifensine administration and a subsequent slow, prolonged decline. This temporal profile is fully consistent with established in vivo microdialysis literature,^82,87^ supporting that the observed temporal variation primarily reflects normal biological heterogeneity among subjects rather than instability of the electrochemical sensing platform. Therefore, these results validate the capability of the multimodal neural probe to provide stable, reproducible neurochemical sensing *in vivo* while faithfully capturing physiologically relevant subject-specific DA dynamics.

The results demonstrate that our proposed multimodal neural probe enables *in vivo* electrochemical and electrophysiological measurements, providing stable neurochemical monitoring alongside neural activity recording. In particular, the electrochemical sensing function exhibited excellent scan-to-scan reproducibility throughout the recording period, confirming the robustness and reliability of the sensing interface under in vivo conditions. Although the absolute electrochemical current measured after implantation was lower than that obtained during in vitro calibration, this reduction in the signal amplitude should not be interpreted as evidence of sensor instability or degraded analytical performance. Rather, it reflects the fundamentally different electrochemical environment encountered *in vivo*, where factors such as tissue encapsulation, molecular diffusion constraints, and biofouling can influence the measured current response.

The reduced electrochemical current amplitude observed *in vivo* does not imply compromised molecular recognition by the DA aptamer,^89^ but rather reflects fundamental disparities in the electrode microenvironment between *in vivo* tissue and *in vitro* electrolyte solutions.^21,90^ In the brain, the electrode surface is rapidly coupled to a heterogeneous electrode-tissue interface comprising cellular membranes, extracellular matrix constituents, and adsorbed biomolecules, which collectively increase effective interfacial impedance.^68^ This manifests as an elevated effective series resistance dominated by electrode-tissue and extracellular matrix contributions,^90^ increased *R*_*ct*_, and reduced effective *CPE*, all of which attenuate faradaic electron-transfer efficiency and suppress measurable redox currents;^91^ accordingly, the reduced electrochemical signal amplitude observed *in vivo* compared with *in vitro* conditions is primarily attributable to increased electrode-tissue impedance and diffusion-limited mass transport rather than diminished aptamer-DA binding, while the excellent scan-to-scan reproducibility confirms that the implanted neural probe can stably and reliably track pharmacologically evoked extracellular DA dynamics *in vivo*.

In addition, molecular transport in the brain extracellular space is diffusion-limited due to tortuosity and local confinement.^92^ Such restricted mass transport limits DA flux to the electrode surface and reduces the number of redox-active events that can be transduced into measurable current.^93^ Together, impedance elevation and mass-transport constraints explain why *in vivo* signals show lower absolute amplitude while still faithfully reporting relative DA dynamics and pharmacologically driven changes.^94^

## Limitations and Future Perspectives

While the present study successfully demonstrates the resilience of the aptameric interface under acute enzymatic challenges under acute enzymatic challenges that simulate the extracellular nucleic acid-rich (cfDNA/NETs) inflammatory microenvironment characteristic of early stage implantation injury,^95^ the comprehensive transition to chronic *in vivo* application presents distinct degradation pathways.^71,96^ Beyond the immediate interfacial impedance and acute nuclease effects investigated here, the long-term operational stability of aptamer-based electrochemical sensors in the brain remains a fundamental challenge for chronic implants. DNA aptamers, including both single- and double-stranded regions, are inherently susceptible to gradual degradation and structural disruption by endogenous DNA-processing enzymes present in biological media and living tissue over extended periods, such as nucleases (e.g., DNase I)^97–99^ and other DNA-processing enzymatic activities.^8,100^

As the tissue response progresses from the acute inflammatory phase to chronic foreign-body reactions, additional dominant failure mechanisms emerge. In addition to prolonged nuclease-mediated degradation, chronic inflammatory and oxidative processes associated with sustained tissue responses, including microglial activation and astrocytic gliosis,^68^ further destabilize the electrode-aptamer interface.^101^ Over months of deployment, phenomena such as severe biofouling, nonspecific protein corona formation, redox tag instability, and the gradual loss of surface-bound aptamers through thiol-gold bond desorption^68,71,99,102^ can severely accelerate signal deterioration.^103^

Beyond interfacial impedance effects, the long-term stability of aptamer-based electrochemical sensing systems also introduces additional concerns regarding the stability and biocompatibility of the integrated on-chip Ag/AgCl RE of the neural probe in this study. Although Ag/AgCl electrodes provide stable electrochemical performance during short-term measurements, prolonged implantation may result in gradual Ag^+^ ion release, inflammatory responses, gliosis, and degradation of interfacial stability under physiological conditions.^104^ Previous studies have shown that degradation of chronically implanted Ag/AgCl electrodes can cause severe local tissue damage, neuronal loss, and persistent glial responses in the brain.^105^ Furthermore, released silver ions have been reported to induce oxidative stress, deplete intracellular antioxidant defenses, activate microglia and astrocytes, and exert cytotoxic effects on neural cells, thereby exacerbating chronic neuroinflammation and foreign-body reactions at the implant-tissue interface.^106^ These effects could compromise both neural tissue compatibility and long-term electrochemical reliability, particularly in chronic neural-interfacing applications. The long-term chemical instability and potential chronic cytotoxicity of electrodeposited Ag/AgCl reference elements resulting from irreversible material degradation and silver ion diffusion over extended time scales were beyond the intended scope of the present acute-phase proof-of-concept investigation and remain an important technological challenge for future chronic *in vivo* translation.

To mitigate these interfacial limitations, several material- and device-level surface-modification strategies have been proposed. To reduce biofouling and nuclease-mediated degradation, previous studies have employed zwitterionic polymer coatings, such as poly­(sulfobetaine methacrylate) (PSB), or related hydrated polymer layers as protective interfacial barriers.^97,107^ These highly hydrated surfaces suppress nonspecific protein adsorption and partially hinder enzymatic access to the aptamer layer, thereby prolonging sensor functionality while preserving electrochemical sensitivity.^107,108^ Similar protective coatings, including Nafion, polyurethane, polyethylene glycol (PEG)-based layers, and other ion-permeable biocompatible polymers, may also reduce Ag^+^ leakage and improve interfacial stability by limiting biomolecular adsorption and inflammatory interactions at the electrode surface.^104^ However, such protective layers may simultaneously alter electron-transfer kinetics, increase diffusion distance, and affect response dynamics, requiring careful optimization to balance long-term stability with sensing sensitivity and temporal resolution.^99^

Transitioning to alternative reference electrode materials offers another crucial pathway toward overcoming the vulnerabilities of the conventional silver-based elements. Materials including iridium oxide (IrOx)^109^ and boron-doped diamond (BDD)^110^ have also been explored due to their improved chemical stability, reduced ion release, and enhanced biocompatibility under physiological conditions.^111^ Meanwhile, modification of the electrode configuration may further reduce local tissue exposure to Ag-based materials. For example, remote or spatially separated REs positioned in subcutaneous tissue or external electrolyte reservoirs have been investigated to minimize direct neural exposure.^112^ Although such approaches may improve biocompatibility, increased electrode separation can introduce higher solution resistance and reduced potential stability, necessitating careful optimization of the system architecture.

At the system level, implementing architectural simplification and advanced operational paradigms can further extend the functional lifetimes of implantable sensors. Simplified two-electrode configurations have been proposed as an alternative to conventional three-electrode systems to eliminate issues associated with the reference electrode degradation. However, these configurations generally provide less accurate potential control and lower electrochemical stability compared with standard three-electrode architectures.^113^ Operational strategies, including intermittent sensing paradigms, replaceable probe designs, and guide-cannula-assisted implantation approaches,^81^ may provide additional pathways toward extending functional lifetime by reducing cumulative exposure of the sensing interface to the biological environment. Such strategies may be particularly advantageous for aptamer-based electrochemical sensors, where biofouling and molecular degradation remain fundamental limitations to chronic applications.

Therefore, while the present study focuses on proof-of-concept acute *in vivo* measurements, future integration of antifouling surface engineering, alternative reference electrode materials, optimized electrochemical configurations, and modular implantation strategies may facilitate the development of more robust and biocompatible chronic neurochemical sensing platforms.

## Conclusions

In this study, we developed a flexible multimodal neural probe that integrates electrochemical DA sensing with electrophysiological recording on a single-microelectrode-array platform. The device incorporates an on-chip three-electrode electrochemical system consisting of an electrodeposited Ag/AgCl reference electrode, a nanostructured platinum counter electrode, and a nanostructured gold working electrode functionalized with a dopamine-specific aptamer. This design provided a stable electrochemical performance with high selectivity, a broad dynamic sensing range, and an experimentally validated detection limit of 10 fM.

Acute *in vivo* experiments further demonstrated that the platform could detect pharmacologically induced changes in extracellular DA together with the corresponding alterations in neuronal electrical activity. Electrochemical sensing and electrophysiological recording were performed sequentially to avoid measurement interference while preserving the multimodal capability within the same implanted neural interface. Because molecular recognition is determined by the aptamer sequence, the sensing strategy can be readily extended to other neurochemical targets through aptamer replacement, providing a versatile platform for investigating neurochemical regulation and neural circuit activity in neurological and psychiatric disorders.

The present work establishes the feasibility of integrating electrochemical sensing and electrophysiological recording within a flexible neural interface under acute *in vivo* conditions. The findings should therefore be interpreted within the context of acute proof-of-concept validation rather than long-term implant performance. Biological responses and material degradation associated with chronic implantation, including biofouling, glial encapsulation, and long-term electrode stability, involve distinct challenges that require dedicated investigation. Future studies incorporating antifouling surface engineering, more chemically stable reference electrode materials, and optimized device architectures will be important for extending this platform toward chronic neurochemical monitoring and long-term neural interfacing.

## Materials and Methods

### Fabrication of the Flexible Multimodal Neural Probe

The flexible multimodal neural probe was fabricated on a glass carrier substrate using a multilayer polyimide (PI)-based microfabrication process. The overall layer configuration is schematically illustrated in Figure 6a, and the detailed probe architecture is summarized in Table S15 (***Supplementary Note 14***). The fabrication process began with sequential spin coating of 15 PI layers (2.5 μm per layer) onto the glass wafer, resulting in a mechanically compliant yet robust base structure. Each layer was thermally cured at 250 °C for 60 min to ensure complete imidization and to establish structural integrity suitable for chronic neural implantation. The cumulative multilayer stacking strategy was adopted to balance flexibility and mechanical stability while minimizing residual stress accumulation.

**Figure 6:**
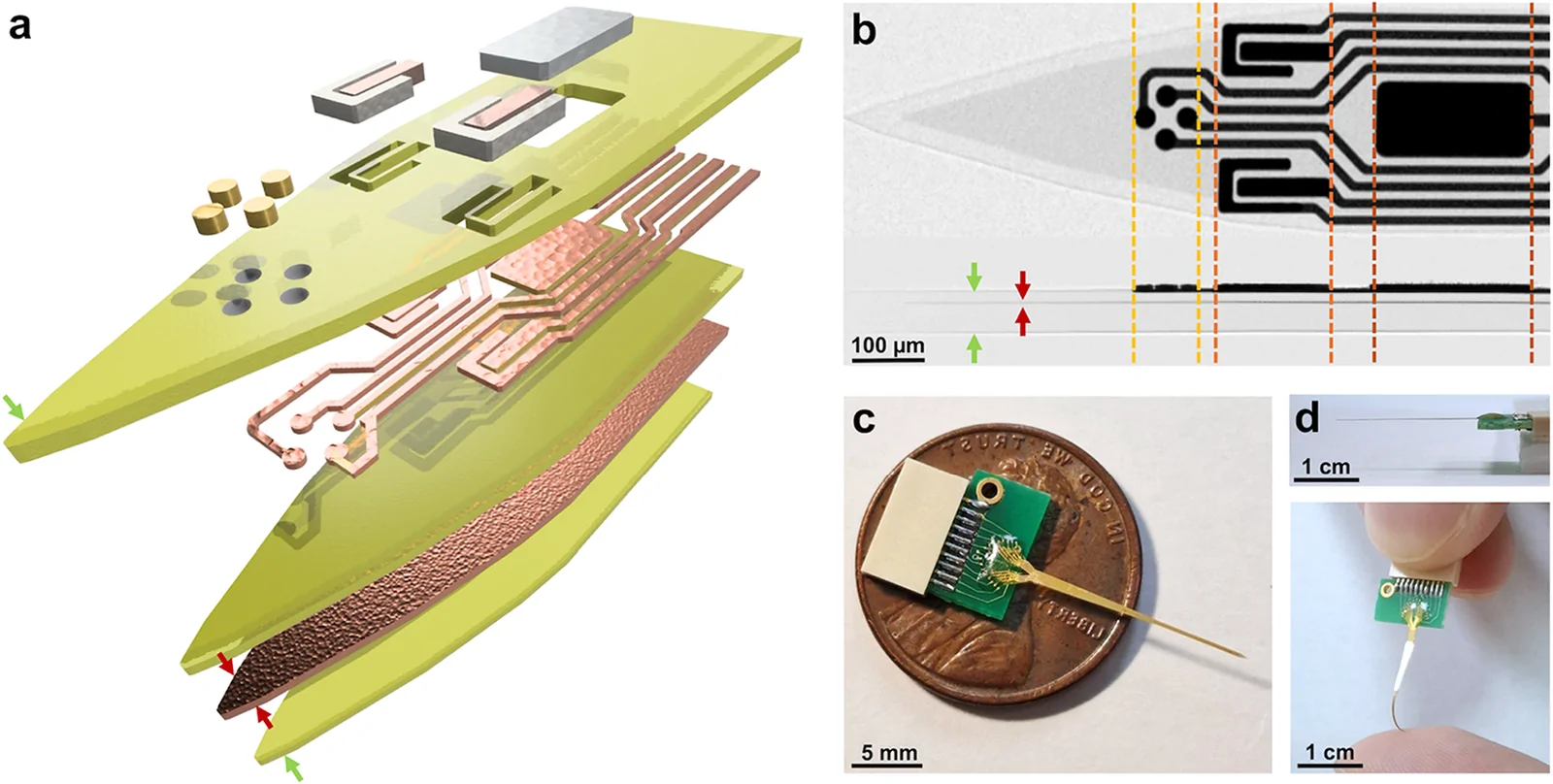
Detailed design, fabrication, and structural characterization of the multimodal neural probe. (a) Exploded layer-by-layer schematic illustrating the architecture of the flexible neural probe, including metal interconnect layers, insulating layers, and the multimodal sensing regions. (b) X-ray computed tomography (CT) penetration scan revealing the structural characteristics of the microelectrode array probe in both horizontal cross section and vertical longitudinal section. In the horizontal cross-sectional view, the region enclosed by the two yellow dashed lines corresponds to four electrophysiological recording electrodes. The region enclosed by the two orange dashed lines indicates the electrochemical recording site, which contains two rectangular WEs and two CEs surrounding the WEs. The region enclosed by the two red dashed lines denotes one RE for the electrochemical sensing system. The vertical longitudinal section shows a magnified view extracted from the squared region marked by an orange dashed box in the full probe image. This close-up highlights the fine structural features, including an overall probe thickness of approximately 60 μm (indicated by green arrows) and the embedded chromium impact layer (Cr impact layer), indicated by red arrows. (c) Photograph of the assembled neural probe bonded onto a nickel-free printed circuit board (PCB) and connected to an Omnetics miniature connector. The enlarged view shows the long probe shaft positioned horizontally, maintaining its straightness and rigidity without noticeable downward slant. (d) Photographs of the neural probe held between fingers, demonstrating its flexibility and delicate nature. The thin and precisely defined probe tip is clearly visible, indicating suitability for implantation while preserving mechanical robustness.

To enhance the structural rigidity without compromising flexibility, a chromium reinforcement layer was deposited onto the cured PI stack. The Cr layer was patterned via standard photolithography and wet-etched by using a KI/I_2_ solution to define the probe backbone geometry. Subsequently, eight additional PI layers were deposited and cured under identical thermal conditions to encapsulate the reinforcement layer and complete the multilayer substrate architecture, yielding a symmetrically stress-balanced configuration.

Electrical routing was formed by sputter deposition of chromium and copper thin films on the PI surface. These metal layers were photolithographically patterned to define the interconnect traces, bonding pads, and electrode sites. A top insulating PI layer was then deposited to electrically isolate the metal networks. Selective openings for electrode sites and contact pads were created by using patterned O_2_ plasma etching to expose the underlying conductive regions.

Gold electrodeposition was subsequently performed on the exposed electrode areas to a thickness of approximately 5 μm, forming biocompatible, low-impedance microelectrodes suitable for electrochemical and electrophysiological measurements. The final probe outline was precisely defined using inductively coupled plasma (ICP) etching to achieve sharp geometric features and a well-defined implantation tip. The completed probe was released from the glass carrier via a KrF 248 nm excimer laser lift-off process, enabling clean separation without mechanical damage.

X-ray computed tomography (CT) imaging (Scientific Gear Service Co., Ltd., Miaoli County, Taiwan) was performed as a nondestructive validation method to examine the internal multilayer architecture, reinforcement integration, and electrode alignment, as shown in Figure 6b. Three-dimensional reconstructions were analyzed to verify the structural continuity, layer stacking fidelity, and dimensional consistency relative to the designed specifications. Quantitative measurements confirmed uniform substrate thickness and accurate spatial positioning of the electrode components, demonstrating fabrication reproducibility and structural integrity.

Following excimer laser lift-off, the probes were mounted onto a custom nickel-free printed circuit board (PCB) for electrical interfacing (Figure 6c). Gold wire bonding connected probe pads to PCB traces under controlled ultrasonic parameters, and the bonding region was epoxy encapsulated to enhance mechanical stability and strain relief. Mechanical compliance was verified through manual bending tests (Figure 6d), confirming the structural integrity of the multilayer stack and bonded interconnects.

The completed multimodal probe integrates four microelectrodes dedicated to electrophysiological recording and two on-chip three-electrode electrochemical sensing systems sharing a common RE, as shown in Figure 7a. After assembly, EIS (CHI760E, CH Instrument Co., Ltd., Austin, TX, USA) was first performed to evaluate the *in vitro* impedance of the electrophysiological recording electrodes. At 1 kHz, the impedance magnitude was 351 kΩ ± 6 Ω, which falls within the suitable range for MUA and LFP recordings. The corresponding impedance spectra are provided in Figure S1 (***Supplementary Note 1***). Subsequent procedures focused on the preparation and functionalization of the electrochemical sensing electrodes with DA-specific aptamers, as described in the following section.

**Figure 7:**
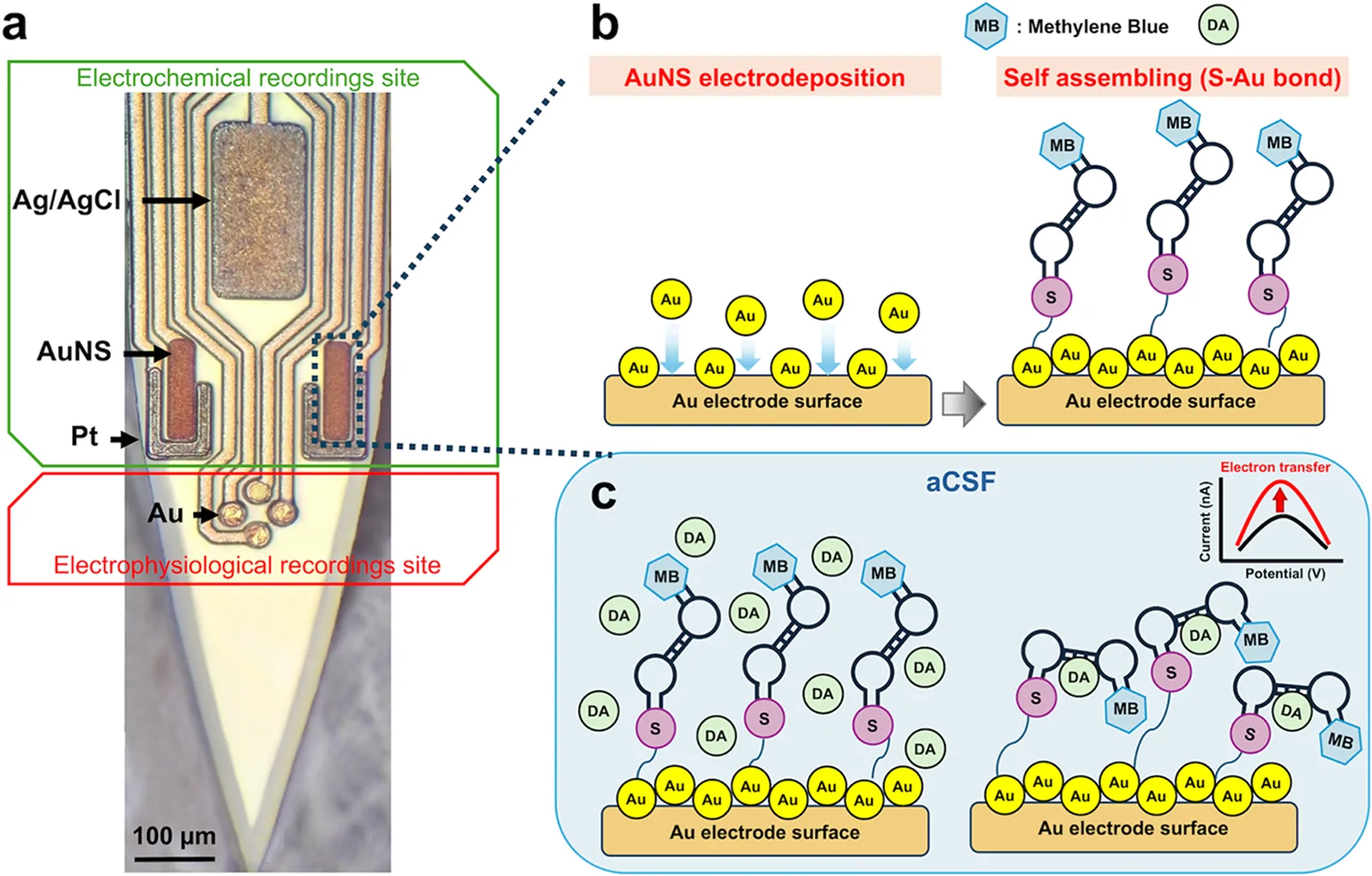
Detailed schematic of the multimodal sensing regions, showing the modification process and the sensing principle. (a) Optical microscope image of the microelectrode array probe tip. The green line indicates the electrochemical recording region, which contains the RE (Ag/AgCl), the WE modified with AuNS, and the counter electrode formed by Pt electroplating. (b) Schematic illustration of the modification process of the WE in the electrochemical recording region. After AuNS were electrodeposited onto the Au electrode surface, the aptamer bearing a sulfhydryl group self-assembled onto the Au surface via Au–S bonding, enabling stable immobilization of the aptamer for DA sensing. (c) Schematic illustration of the sensing principle on the WE. Upon binding to DA molecules, the aptamer undergoes a conformational change, altering the distance between the MB label and the Au electrode surface, which results in a measurable change in the current signal.

### Surface Modification of the On-Chip Three-Electrode Electrochemical System of the Neural Probe

To optimize the ECSA of the WE within the integrated on-chip three-electrode electrochemical sensing system, AuNSs were electrodeposited onto the WE by chronoamperometry using a Squidstat Plus potentiostat (Admiral Instruments, Tempe, AZ, USA) in a 10 mM tetrachloroauric (III) acid (HAuCl_4_, ≥ 99.9%, Merck KGaA, Darmstadt, Germany) solution. Constant deposition potentials of 0.50, 0.55, 0.60, 0.65, and 0.70 V vs Ag/AgCl were individually applied for 3 min (Figure 7b, left panel) to evaluate the influence of electrodeposition potential on the electrochemical and structural properties of the AuNS-modified WEs.

A total of 18 independently fabricated neural probes were equally divided into six fabrication groups consisting of nonelectrodeposited WEs (control) and WEs subjected to AuNS electrodeposition at 0.50, 0.55, 0.60, 0.65, and 0.70 V. Each fabrication group contained three independently fabricated neural probes, with two independent WEs integrated on each probe, resulting in a total of *N* = 6 independent WEs for each electrodeposition condition.

The electrochemical properties of the fabricated WEs were first characterized by cyclic voltammetry in 0.1 M H_2_SO_4_ to determine the ECSA according to standard electrochemical procedures (Supporting Information Note 2). Mean cyclic voltammograms obtained from the six independently fabricated WEs were used to calculate the corresponding ECSA values, while probe-to-probe reproducibility was evaluated using the CV %. The normalized ECSA values were subsequently used to compare the effectiveness of the different electrodeposition conditions for increasing the electrochemically active surface available for subsequent aptamer immobilization.

To quantitatively characterize the surface morphology of the AuNS-modified WEs, atomic force microscopy (AFM, VK-X3000, Keyence Co., Itasca, IL, USA) was performed on all independently fabricated WEs (*N* = 6 for each electrodeposition condition). Three-dimensional surface topography and corresponding surface profiles were acquired, and the arithmetic surface roughness (S_a_) was quantified using instrument analysis software. Probe-to-probe reproducibility of the surface roughness was evaluated using the CV %.

To examine the relationship between electrochemical and structural characteristics, the mean ECSA values obtained from cyclic voltammetry were correlated to the corresponding AFM-derived S_a_ values for each electrodeposition condition. In addition, optical microscopy was performed to examine the geometric integrity of the electrodeposited AuNS layer and to evaluate the potential AuNS overflow beyond the designed WE boundary under different electrodeposition conditions. The electrochemical, structural, and fabrication characterizations were collectively used to compare the different electrodeposition conditions and determine the optimal AuNS electrodeposition parameters.

Surface morphology and elemental composition of the AuNS-modified WEs were also characterized using ultrahigh-resolution field-emission scanning electron microscopy (FE-SEM, JSM-7800F, JEOL Ltd., Akishima, Japan) equipped with energy-dispersive X-ray spectroscopy (EDS, X-MAXn 80, Oxford Instruments plc, High Wycombe, UK). FE-SEM was used to examine the surface morphology of the electrodeposited Au nanostructures, while EDS elemental mapping and spectra were employed to verify the elemental composition of the modified WE (Figure S6, ***Supporting Information Note 3***).

To complete the integrated three-electrode configuration, the two CEs were modified with platinum by cyclic voltammetric electrodeposition in 12.5 mM hexachloroplatinate (H_2_PtCl_6_, ≥ 99.9%, Merck KGaA, Darmstadt, Germany) containing 0.1 M sodium nitrate (NaNO_3_, PanReac AppliChem, Darmstadt, Germany) over the potential range of −0.2 to 1.5 V at a scan rate of 50 mV s^–1^ for five cycles. The RE was fabricated by electrodepositing silver from 12.5 mM silver nitrate (AgNO_3_, 99.85%, Acros Organics, Belgium) in 0.1 M NaNO_3_, followed by electrochemical chloridization in 1 M KCl using sequential potential steps of 0.2, 0.5, 0.75, and 1.0 V (40 s each) to form an Ag/AgCl layer.

Following electrode fabrication, the Pt-coated CEs and Ag/AgCl RE were further characterized by FE-SEM and EDS to evaluate their surface morphology and elemental distribution (Figures S7 and S8, ***Supporting Information Note 3***). Together with the characterization of the AuNS-modified WE, these analyses verified the successful electrode-specific surface modification and elemental composition of all three-electrode components comprising the integrated on-chip three-electrode system prior to subsequent electrochemical sensing experiments.

To evaluate the electrochemical stability of the integrated on-chip Ag/AgCl RE, we performed OCP measurements under physiologically relevant conditions. The experimental configuration is illustrated in Figure S9 of ***Supporting Information Note 4***. Measurements were conducted using a Squidstat Plus potentiostat (Admiral Instruments, Tempe, AZ, USA), with the integrated Ag/AgCl electrode serving as the WE and a commercial saturated calomel electrode (SCE; Aurora Borealis Technology Co., Ltd., Taipei City, Taiwan) serving as the reference electrode. Both electrodes were immersed in artificial cerebrospinal fluid (aCSF) without applying an external current or potential bias. After a 10 min equilibration period, the open-circuit potential was continuously recorded for 3,600 s with a sampling interval of 0.5 s. The OCP measurement was designed to evaluate the intrinsic short-term electrochemical stability of the integrated Ag/AgCl RE over the time scale relevant to the acute electrochemical sensing experiments performed in this study. Detailed analyses of the OCP behavior and corresponding stability evaluation are provided in ***Supporting Information Note 4*** (Figures S9 and S10).

### Optimization and Functionalization of the On-Chip Electrochemical Sensing WE

Building upon the AuNS-modified WEs fabricated under different electrodeposition potentials as described in the previous section, subsequent experiments focused on aptamer functionalization and electrochemical performance optimization of the sensing interface. The DA aptamer sequence (5′-3′) used in this study was adopted from previously reported electrochemical DA aptasensors and DA aptamer literature:^114,115^ HO–C6–S-S-C6-CGA CGC CAG TTT GAA GGT T-MB-CG TTC GCA GGT GTG GAG TGA CGT CG. The core 44-mer DA-recognition sequence has been widely employed in electrochemical and FET-based DA biosensors.^115^ To verify that the electrochemical response originated from sequence-specific DA recognition rather than nonspecific adsorption, a composition-matched scrambled aptamer was designed as a negative control. The scrambled aptamer retained the same 5′ disulfide linker, overall sequence length (44-mer), nucleotide composition, GC content, internal MB labeling position, and surface immobilization chemistry as the functional DA aptamer, while the nucleotide order within the recognition region was rearranged to disrupt dopamine-binding capability. Consequently, both probes were expected to exhibit comparable surface coverage and electron-transfer characteristics, allowing differences in electrochemical response to be attributed primarily to sequence-specific target recognition rather than variations in probe immobilization or interfacial properties. The sequences of both probes are summarized in Table S4 of ***Supplementary Note 6***. A 5′ disulfide linker (HO–C6–S-S-C6-) was introduced to enable Au–S immobilization onto the AuNS-modified WE, while MB was inserted between the 19th and 20th nucleotides as an electrochemical redox reporter. This internal MB-labeling strategy has previously been shown to enhance coupling between aptamer conformational changes and electron-transfer efficiency in electrochemical aptamer-based sensors.^114,116^

Prior to immobilization, both the functional DA aptamer and the scrambled aptamer were thermally preconditioned at 95 °C for 5 min and cooled to room temperature for 15 min to promote proper folding. The terminal disulfide bond was subsequently reduced using 10 mM tris (2-carboxyethyl) phosphine (TCEP, ≥ 98%, Sigma-Aldrich, St. Louis, MO, USA) for 1 h to generate free thiol groups for surface attachment. The activated functional and scrambled aptamers were independently immobilized on AuNS-modified WEs for 16 h at room temperature in the dark, allowing spontaneous Au–S bond formation, as shown in Figure 7b (right panel). Subsequently, the electrode surface was treated with 2 mM 6-mercapto-1-hexanol (6-MCH, 97%, Sigma-Aldrich, St. Louis, MO, USA) for 30 min to passivate unoccupied gold sites and minimize nonspecific adsorption.

The electrochemical response of the aptamer-functionalized WEs prepared using different AuNS deposition potentials was evaluated by SWV using a commercial platinum CE (part number 002233, ALS Co., Ltd., Tokyo, Japan) and a commercial Ag/AgCl RE (RE-1S, ALS Co., Ltd., Tokyo, Japan). The sensing mechanism is based on the conformational switching of the MB-labeled aptamer upon DA binding, which modulates the electron-transfer distance between the redox reporter and the Au/AuNS surface, thereby producing measurable changes in the faradaic current (Figure 7c). SWV measurements were performed over a potential window from 0 to −0.5 V with a step potential of 5 mV, an amplitude of 25 mV, and an interrogation frequency of 100 Hz. The SWV amplitude and step potential were selected according to operating conditions widely adopted for MB-based electrochemical aptamer sensors reported in the literature.^38,40,41^ The interrogation frequency was experimentally validated for the present AuNS-modified sensing interface of the neural probe through a frequency-dependent evaluation (10–200 Hz), and 100 Hz was selected based on the results of a frequency-dependent experimental evaluation performed for the present sensing interface. The corresponding validation data and rationale are provided in the Supplementary Note 5. SWV measurements were acquired in repeated measurement cycles, each consisting of a 3 s scan followed by a 7 s idle interval. The raw current signals were processed using a zero-phase fourth-order Butterworth low-pass filter with a cutoff frequency of 2 Hz, followed by baseline correction using the current measured at −0.3 V as the reference to obtain the corrected SWV peak current. The corrected peak-current responses obtained from WEs fabricated under different AuNS deposition potentials were compared to identify the optimal AuNS deposition condition for subsequent sensing experiments. For sequence-specificity validation, identical SWV operating parameters were subsequently applied to both the functional and scrambled aptamer-functionalized electrodes.

EIS measurements were performed after each surface-modification step, including AuNS deposition, aptamer immobilization, and 6-MCH backfilling. Impedance spectra were acquired in 5 mM [Fe (CN)_6_]^3–/4–^ containing 0.1 M KCl over a frequency range from 0.1 Hz to 100 kHz by applying a small sinusoidal perturbation of 5–10 mV at the open-circuit potential. The obtained Nyquist plots were fitted using ZView software (Scribner Associates Inc., Southern Pines, NC, USA) through nonlinear least-squares regression based on a modified Randles equivalent circuit with an *R*_*S*_ – (*CPE* ∥ (*R*ct + *Z*_*w*_)) configuration, where *R*_*S*_ represents the solution resistance, *CPE* denotes a constant phase element, *R*_*ct*_ corresponds to the charge-transfer resistance, and *Z*_*w*_ accounts for the diffusion-related impedance. All fitting parameters were extracted from the complex impedance data using the ZView software’s built-in complex nonlinear least-squares (CNLS) algorithm. The goodness of fit was evaluated by the chi-square value and by inspection of the residual distribution between the experimental and simulated spectra.

In addition, XPS (Quantes, ULVAC-PHI, Inc., Chigasaki, Japan) analysis was performed on the AuNS-modified WEs before and after aptamer functionalization (Supporting Information Note 7). High-resolution spectra of the *Au 4f*, *C* 1*s*, and S 2p regions were used to identify spectral features consistent with thiol-mediated Au–S bond formation and the presence of organic functional groups, confirming stable immobilization of the DA-binding aptamer on the electrode surface.

### *In Vitro* and *Ex Vivo* Electrochemical Experiments

To evaluate the influence of electrode configuration on electrochemical sensing performance, the aptamer-modified neural probe was characterized using both a conventional three-electrode configuration and an integrated on-chip three-electrode configuration. In the conventional configuration, the AuNS-modified WE on the neural probe was operated together with an externally positioned platinum CE and a commercial Ag/AgCl RE. In the integrated on-chip configuration, AuNS-modified WE, CE, and RE were monolithically integrated on the same neural probe substrate, forming a localized on-chip three-electrode electrochemical system. All electrochemical measurements were performed in the aCSF.

SWV measurements were conducted over a DA concentration range from 5 fM to 10 μM to characterize the concentration-dependent electrochemical response of the sensing platform. The selected concentration range encompassed DA concentrations near the experimentally determined detection limit as well as concentrations approaching signal saturation, providing data sets for subsequent evaluation of the calibration characteristics, detectable concentration range, absolute linear response range, and saturation behavior. The electrochemical response was quantified as the change in SWV peak current (Δ*I*) relative to the DA-free aCSF condition.

Measurements were obtained from three independently fabricated neural probes, each containing two independent of-chip three-electrode electrochemical sensing units, resulting in a total of *N* = 6 independent sensing units. For each sensing unit, electrochemical measurements at every DA concentration were repeated 10 times. Mean values, SDs, and CV % values were calculated for each concentration condition.

The blank signal distribution was determined from repeated DA-free aCSF measurements obtained from the six independent sensing units, and the experimentally validated LOD was determined using the conventional signal-to-noise criterion based on three times the standard deviation of the blank signal (3 × σ_blank_). Quantitative calibration analysis was performed using concentration-dependent SWV peak-current responses. Detailed calibration fitting procedures, statistical analyses, blank-signal characterization, and LOD determination methods are provided in Supplementary Note 9.

Based on the calibration analysis shown in Figure 3c and Figure S20 (***Supporting Information Note 9***), the integrated on-chip three-electrode configuration exhibited improved sensitivity, enhanced fitting linearity, lower baseline variability, and a lower experimentally validated LOD compared with the conventional three-electrode configuration. Therefore, the integrated on-chip three-electrode system was used in all subsequent experiments, including selectivity analysis, FIA, and *ex vivo* brain-phantom experiments.

Selectivity tests were conducted in aCSF (pH 7.1) using DA at a concentration of 1 pM. Potential interferents included AA, l-Tyr, L-DOPA, 5-HT, DOPAC, HVA, UA, and 3-MT. To provide a stringent and physiologically relevant assessment of selectivity, l-Tyr, L-DOPA, 5-HT, DOPAC, HVA, UA, and 3-MT were tested at concentrations of 1 nM (10^3^-fold excess relative to DA), whereas AA was evaluated at 1 μM (10^6^-fold excess relative to DA) due to its substantially higher extracellular concentration in brain tissue. Each condition was measured in triplicate using identical SWV acquisition and signal-processing procedures. The electrochemical response was quantified from the MB redox current change (Δ*I*), and statistical significance between DA and each interferent was evaluated using a two-sample *t*-test. Because the sensing mechanism is based on aptamer-binding-induced conformational switching rather than direct DA oxidation, electroactive interferents such as AA and UA are not expected to produce substantial signal responses in the absence of specific aptamer recognition.^27,117^

FIA was performed to simulate the dynamic processes of DA release, diffusion, and clearance and to evaluate the real-time response and repeatability of the aptamer-based electrochemical sensor under continuous-flow conditions. The FIA setup is illustrated in Figure 8 and consists of a syringe pump, a multiport injection valve, and a flow cell containing the neural probe connected to the potentiostat. The neural probe with an integrated on-chip three-electrode system was immersed in a reservoir filled with aCSF (pH 7.1), while the carrier solution was continuously delivered through the flow loop at a constant rate.

**Figure 8:**
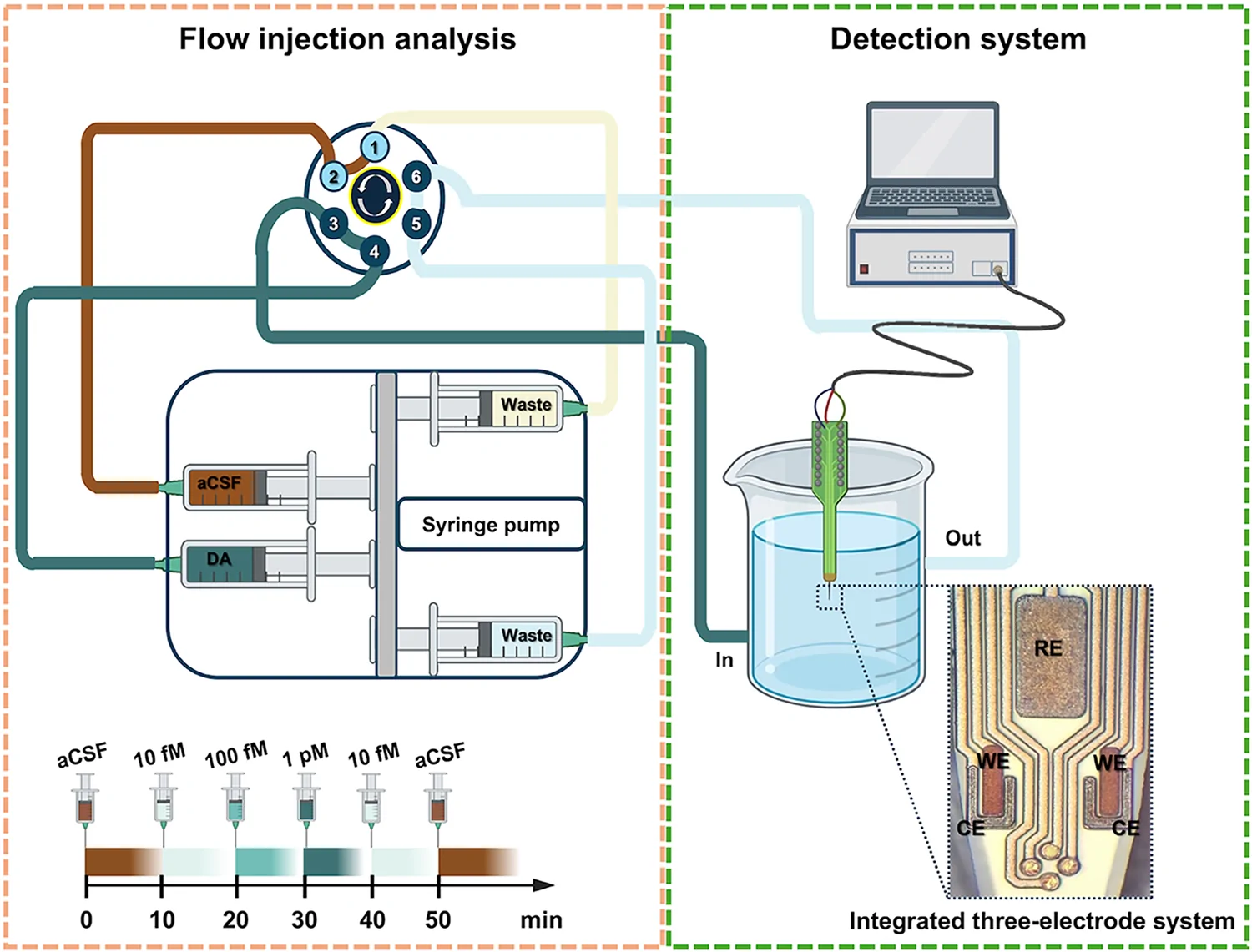
Scheme of the *in vitro* intermittent electrochemical DA monitoring system. It is divided into two parts, which were FIA (the orange blocking region) combined in our detection system (the green blocking region) for *in vitro* DA sensing by using the integrated microelectrode array probe. Every 10 min, aCSF, 10 fM DA, 100 fM DA, 1 pM DA, 10 fM DA, and aCSF were sequentially injected into the detection system to monitor time-dependent signal changes through repeated SWV measurements.

All FIA experiments were conducted using the on-chip three-electrode neural probe. The flow rate was maintained at 0.1 mL min^–1^ with an injection volume of 1 mL. Prior to DA injection, a 10 min baseline measurement was performed in aCSF to ensure system stability. DA solutions were sequentially introduced at concentrations of 10 fM, 100 fM, and 1 pM. Each concentration was monitored for 10 min, including both wash-in and wash-out phases, before switching to the next concentration. After the highest concentration step, the DA solution was returned to the initial low concentration (10 fM) to evaluate signal reversibility, hysteresis, and baseline stability.

Electrochemical measurements were performed using SWV with a step potential of 5 mV, frequency of 100 Hz, amplitude of 25 mV, and a potential window from 0 to −0.5 V. Each measurement cycle consisted of a 3 s SWV scan followed by a 7 s idle interval. Measurements were conducted once per minute. For each time point, three consecutive SWV scans were collected and averaged to obtain a single current value for each WE of the neural probe.

For FIA experiments, measurements were performed using three independently fabricated neural probes (*N* = 3), with one on-chip three-electrode electrochemical system (one WE) selected from each probe. The reported signal at each time point represents the mean response across these three independent WEs of the neural probe, and the corresponding error bars represent the SD across the devices.

*Ex vivo* brain-phantom experiments were performed to simulate probe implantation conditions in the brain tissue. The neural probe was inserted to a depth of 4.5 mm into a 0.6% agarose gel prepared in aCSF, corresponding to the target depth of the CPu region in the animal experiments. After insertion, the system was allowed to equilibrate for 10 min to stabilize the electrode–electrolyte interface. Electrochemical measurements were carried out using the same SWV parameters and acquisition protocol as those described for the FIA experiments.

During the initial phase, 1 μL of aCSF was injected near the probe after each measurement to confirm baseline stability. Subsequently, DA solutions at concentrations of 10 fM, 1 pM, and 100 pM were sequentially introduced. After each injection, a diffusion period of 1 min was allowed prior to the next measurement. Each data point represents the averaged current response obtained from three consecutive SWV scans per electrode, followed by averaging across three independently fabricated neural probes (*N* = 3), ensuring consistency with the FIA measurement protocol.

### *In Vivo* Animal Experiments

Three male Sprague–Dawley rats (8 weeks old, 200–250 g; National Laboratory Animal Center, Taiwan) were housed under standard conditions (12:12 h light/dark cycle, 20 ± 3 °C) with free access to food and water. All animal procedures were approved by the Institutional Animal Care and Use Committee of Taipei Medical University (Approval No. LAC-2019–0370) and were conducted in accordance with the approved institutional guidelines for the care and use of laboratory animals.

Under 2% isoflurane anesthesia, rats underwent right femoral vein catheterization through a 1.5 cm incision above the inguinal region. The femoral vein was carefully isolated, ligated proximally and distally, and cannulated with a catheter secured using silk sutures. Proper catheter placement was confirmed by saline injection. For intracranial recordings, animals were mounted in a stereotaxic frame, and body temperature was maintained at 37 °C using a feedback-controlled heating pad. A craniotomy was performed above the CPu (AP + 1.08 mm, ML ± 2.4 mm), and the multifunctional neural probe was implanted to a depth of 4.5 mm below the cortical surface. A stainless steel screw placed over the cerebellum served as the electrophysiological reference electrode. Following implantation, the probe was allowed to stabilize in situ for 30 min to minimize acute insertion-induced disturbances and to ensure stable electrochemical and electrophysiological recordings.

Electrochemical measurements were performed using a Squidstat Plus potentiostat (Admiral Instruments, Tempe, AZ, USA), while electrophysiological signals were acquired with the Open Ephys recording system (OEPS, Lisbon, Portugal), as shown in Figure 9a. Because SWV involves rapidly varying electrode potentials that may introduce capacitive and stimulation-related artifacts into neural recordings,^118,119^ a time-division multiplexed acquisition strategy was implemented.^8,120^ Electrochemical sensing and electrophysiological recording were performed in nonoverlapping temporal windows within each measurement cycle to suppress cross-modal interference.^119^ Each measurement cycle had a total duration of 70 s and consisted of three sequential phases: (1) electrochemical sensing, (2) an electrical stabilization interval, and (3) electrophysiological recording. This cycle was repeated continuously throughout the experiment. During the electrochemical sensing phase (30 s), DA levels were measured using an aptamer-based electrochemical sensor operated in SWV mode. SWV measurements were performed with a step potential of 5 mV, frequency of 100 Hz, amplitude of 25 mV, and a potential window from 0 to −0.5 V relative to the embedded RE of the neural probe. The sequential measurement protocol was automatically controlled by a custom synchronization script interfacing with the potentiostat application programming interface (API)^121^ and the electrophysiological acquisition software.^122^ The control script sequentially triggered SWV scans and electrophysiological recording windows according to the predefined timing schedule, and all events were logged with synchronized timestamps to ensure accurate temporal alignment during offline/post hoc analysis.

**Figure 9:**
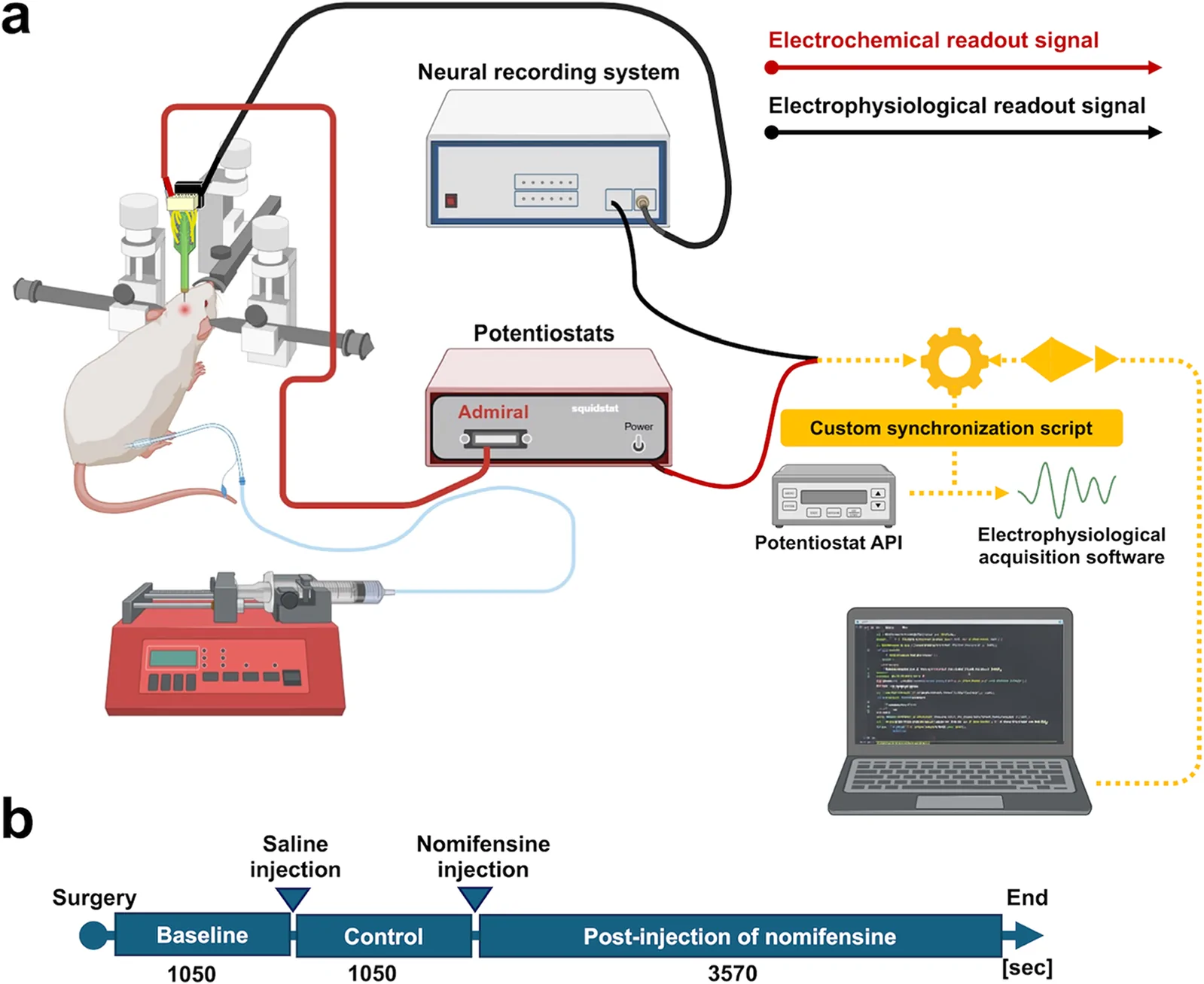
Scheme of the *in vivo* experimental setup. (a) Integrated *in vivo* experimental setup enabling synchronized electrochemical and electrophysiological recordings. The multifunctional neural probe was implanted into the target brain region and connected simultaneously to a potentiostat for SWV measurements and to a neural recording system for broadband electrophysiological acquisition. A custom synchronization script interfaced with the potentiostat API and the electrophysiological acquisition software to automatically coordinate sequential multimodal measurements. Electrochemical and neural signals were acquired in repeated 70 s cycles consisting of a 30 s electrochemical sensing phase, a 10 s idle interval, and a 30 s electrophysiological recording phase. Saline and nomifensine were administered via intravenous injection. (b) Experimental timeline following probe implantation. After a 1,050 s baseline stabilization period, saline was injected, and signals were recorded for an additional 1,050 s. Subsequently, nomifensine was administered intravenously, followed by 3,570 s of repeated intermittent electrochemical measurements performed at 1 min intervals. Multimodal measurements were performed sequentially every 70 s to capture dynamic changes in DA-dependent electrochemical signals and associated electrophysiological activity.

Within each electrochemical phase, three SWV scans were conducted; each scan consisted of a 3 s SWV sweep followed by a 7 s rest period, yielding a total duration of 10 s per scan. The three scans were averaged to obtain a single DA current reading for each measurement cycle. Following electrochemical sensing, a 10 s idle interval was introduced to allow dissipation of residual capacitive transients and electrode polarization effects. Electrophysiological signals were then recorded for 30 s using a multichannel neural recording system to capture wideband neural activity, including LFPs and spiking activity. The 70 s measurement cycle was repeated continuously for a total recording duration of 5670 s. The full recording session was divided into three experimental stages (Figure 9b). The first stage corresponded to a 1050 s baseline stabilization period. This was followed by a second stage of 1050 s after intravenous saline injection, serving as a vehicle control. The final stage consisted of 3570 s of monitoring after nomifensine administration to evaluate DA-related neurochemical and electrophysiological responses.

Wideband electrophysiological signals were acquired using an Open Ephys Acquisition Board operating at a sampling rate of 30 kHz. The broadband neural recordings were digitally filtered to extract MUA and LFP components. To isolate the MUA band, a high-pass filter at 300 Hz was applied. High-frequency MUA signals were thresholded at negative four times the SD (−4σ) of the waveform noise level, and threshold-crossing events were defined as spikes. Spike counts were calculated within each 30 s recording interval, and the spike counts from the four recording channels of the neural probe were averaged to obtain a representative MUA spike count per interval.

To extract LFP signals, the broadband data were band-pass-filtered between 0.1 and 300 Hz. The LFP signals were subsequently downsampled to 1 kHz for spectral analysis. For each 30 s neural recording interval, the PSD was computed using Welch’s method (MATLAB pwelch function). The LFP band power was defined as the integral of the PSD within specific frequency ranges. The resulting power values were then converted to logarithmic power (dB). The LFP signals were further divided into six canonical frequency bands: δ­(1–3 Hz), θ­(4–7 Hz), α (8–15 Hz), β (16–32 Hz), γ (33–90 Hz), and γ′ (91–300 Hz). For each band, the mean power within the defined frequency range was calculated to represent the LFP band power during each 30 s recording interval.

## Supplementary Material



## Notes

Additional experimental details, electrochemical characterization, electrodeposition optimization, surface morphology/compositional analyses, reference electrode stability evaluation, SWV frequency optimization, aptamer functionalization, analytical performance and reproducibility analyses, LOD validation, and neural probe specifications. Supplementary figures include: FS1, electrophysiological electrode characterization; FS2–FS5, AuNS electrodeposition optimization; FS6–FS9, electrode characterization; FS10–FS11, SWV frequency optimization; FS12–FS16, aptamer functionalization and scrambled-sequence control; and FS17–FS20, calibration, reproducibility, and sensing performance. Supplementary tables include: TS1, electrophysiological electrode reproducibility; TS2–TS5, electrochemical and surface characterization; TS6, DA sensing statistics and LOD validation; TS7, comparison with reported DA aptasensors; and TS8, neural probe specifications (PDF)
