# Supplementary material for: Flexible Multimodal Neural Probe with Integrated Three-Electrode Aptameric Sensing for In Vivo Monitoring of Dopamine Dynamics and Neural Activity
Source: ACS Chem Neurosci. 2026 Aug 6;17(16):3122–49. doi: 10.1021/acschemneuro.6c00550 (PMC13495676; doi:10.1021/acschemneuro.6c00550)
Supplement: Supplementary file 1 [file cn6c00550_si_001.pdf]

Supplementary Materials

A Flexible Multimodal Neural Probe with Integrated Three-Electrode Aptameric Sensing for *In Vivo* Monitoring of Dopamine Dynamics and Neural Activity

Szu-Ying Li[1], Yun-Ting Kuo[1], Sheng-Huang Lin[2,3], Shun-An Kan[4], Bo-Wei Chen[1], Ssu-Ju Li[1], Ching-Wen Chang[1], Han-Lin Wang[1], Yu-Chun Lo[5], and You-Yin Chen[1,5,*]

[1] Department of Biomedical Engineering, National Yang Ming Chiao Tung University, No.155, Sec.2, Linong St., Taipei City 112304, Taiwan
[2] Department of Neurology, Hualien Tzu Chi Hospital, Buddhist Tzu Chi Medical Foundation, No. 707, Sec. 3, Zhongyang Rd., Hualien City 97002, Taiwan
[3] Department of Neurology, School of Medicine, Tzu Chi University, No. 701, Sec. 3, Zhongyang Rd., Hualien City 97004, Taiwan
[4] Department of Education, Taipei Veterans General Hospital, No.201, Sec. 2, Shipai Rd., Taipei City 11217, Taiwan,
[5] Ph.D. Program in Medical Neuroscience, College of Medical Science and Technology, Taipei Medical University. 12F., Education & Research Building, Shuang-Ho Campus, No. 301, Yuantong Rd., New Taipei City 23564, Taiwan

[*] Correspondence should be addressed to the following:
You-Yin Chen, Ph.D.
Department of Biomedical Engineering, National Yang Ming Chiao Tung University, No.155, Sec.2, Linong St., Taipei 112304, Taiwan
E-mail: irradiance@so-net.net.tw/youyin.chen@nycu.edu.tw

Short title: Multimodal neural probe for dopamine and neural activity monitoring

Note 1. Impedance characterization of electrophysiological recording microelectrodes

EIS measurements were performed in physiological saline (0.9% NaCl) maintained at 37 °C using a standard three-electrode configuration, with the recording microelectrode serving as the working electrode, a commercial Ag/AgCl electrode as the reference electrode, and a platinum counter electrode. A 10 mV sinusoidal perturbation was applied over the frequency range from 0.1 Hz to 100 kHz. Measurements were obtained from three independently fabricated neural probes, each containing four electrophysiological recording microelectrodes (total N = 12 recording electrodes). The resulting impedance spectra (**Figure S1**) are presented as mean ± SD, with the shaded region indicating the SD across all recording electrodes.

Because the impedance at 1 kHz is widely accepted as the standard metric for evaluating extracellular recording electrodes, we further quantified the impedance magnitude at this frequency as shown in **Table S1**. The recording microelectrodes exhibited a mean impedance of 366.02 ± 18.63 kΩ at 1 kHz, corresponding to an inter-probe CV% of 5.09%. Within individual neural probes, the mean impedances were 375.85 ± 4.79 kΩ, 359.25 ± 31.78 kΩ, and 361.10 ± 21.27 kΩ, corresponding to within-probe CV% values of 1.27%, 8.85%, and 5.89%, respectively. These results demonstrate excellent reproducibility both among recording electrodes integrated within the same neural probe and across independently fabricated neural probes.

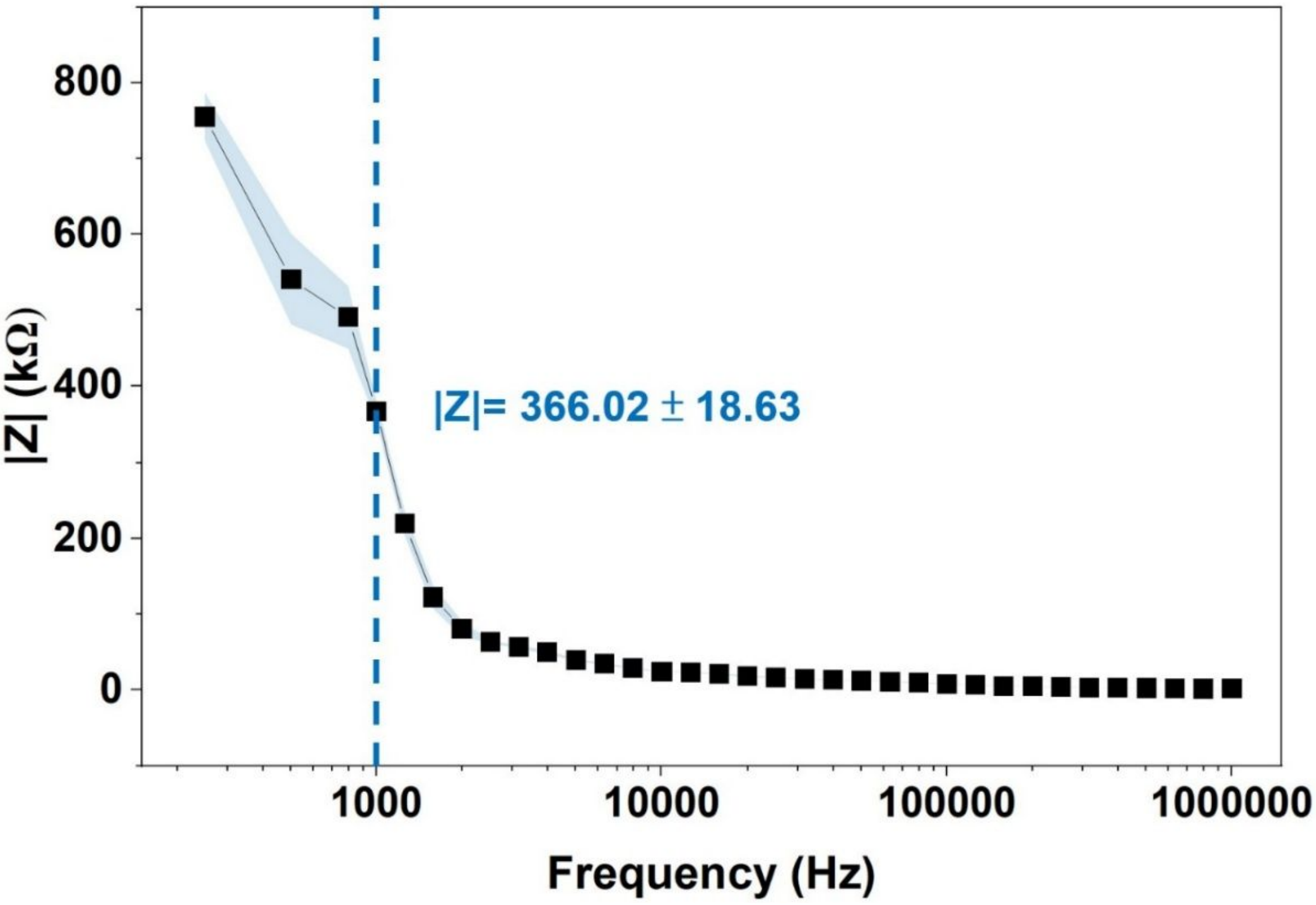

Figure S1. **Impedance characterization of the electrophysiological recording microelectrodes**. EIS of the electrophysiological recording microelectrodes measured in physiological saline. Measurements were obtained from three independently fabricated neural probes, each containing four recording microelectrodes (total N = 12 from three independently fabricated neural probes [Probe #1-#3]). The average impedance at 1 kHz is 366.02 ± 18.63 kΩ, falling within the commonly accepted range for stable neural spike and LFP recordings. The solid line represents the mean impedance magnitude, and the shaded region indicates ± SD. The dashed line denotes the impedance magnitude at 1 kHz.

Table S1. **Within-probe and probe-to-probe reproducibility of the electrophysiological recording microelectrodes**. Impedance magnitude at 1 kHz measured from the electrophysiological recording microelectrodes integrated on three independently fabricated neural probes. Within-probe reproducibility was evaluated using the four recording microelectrodes on each neural probe (N =4), whereas probe-to-probe reproducibility represents the pooled results from the three neural probes (N =12). Data are presented as mean ± SD with the corresponding CV%.

Neural probe ID	Within-probe		Inter-probe	
	Impedance (kΩ)	CV%	Impedance (kΩ)	CV%
**Neural probe #1**	375.85 ± 4.79	1.27	366.02 ± 18.63	5.09
**Neural probe #2**	359.25 ± 31.78	8.85		
**Neural probe #3**	361.10 ± 21.27	5.89		

Note 2. Electrochemical deposition on* WE of the neural probe *and corresponding ECSA measurement and morphological evolution

The ECSAs of gold WEs of microelectrode array probes were determined using a cyclic voltammograms method in which the AuNS-modified WEs were submerged in 0.1 M H_2SO_4 solution and the potential was cycled between 0 and 1.4 V at the potential sweep rate of 100 mV/sec. ECSA (Λ) was calculated using the following equation[1]:

$$\Lambda = \frac{A_{\mathrm{RED}}}{s \times Q_{\sigma d}}$$

where A_{RED} is the integrated area of the reduction peak corresponding to gold oxide on the investigated AuNS-modified WE from the acquired cyclic voltammetry curve, s is the potential sweep rate of 100 mV/sec, and $Q_{\sigma d}$ is the charge density associated with the electrochemical reduction of a monolayer of chemisorbed oxygen on polycrystalline gold using Woods' value of 386 μC/cm^2 [2].

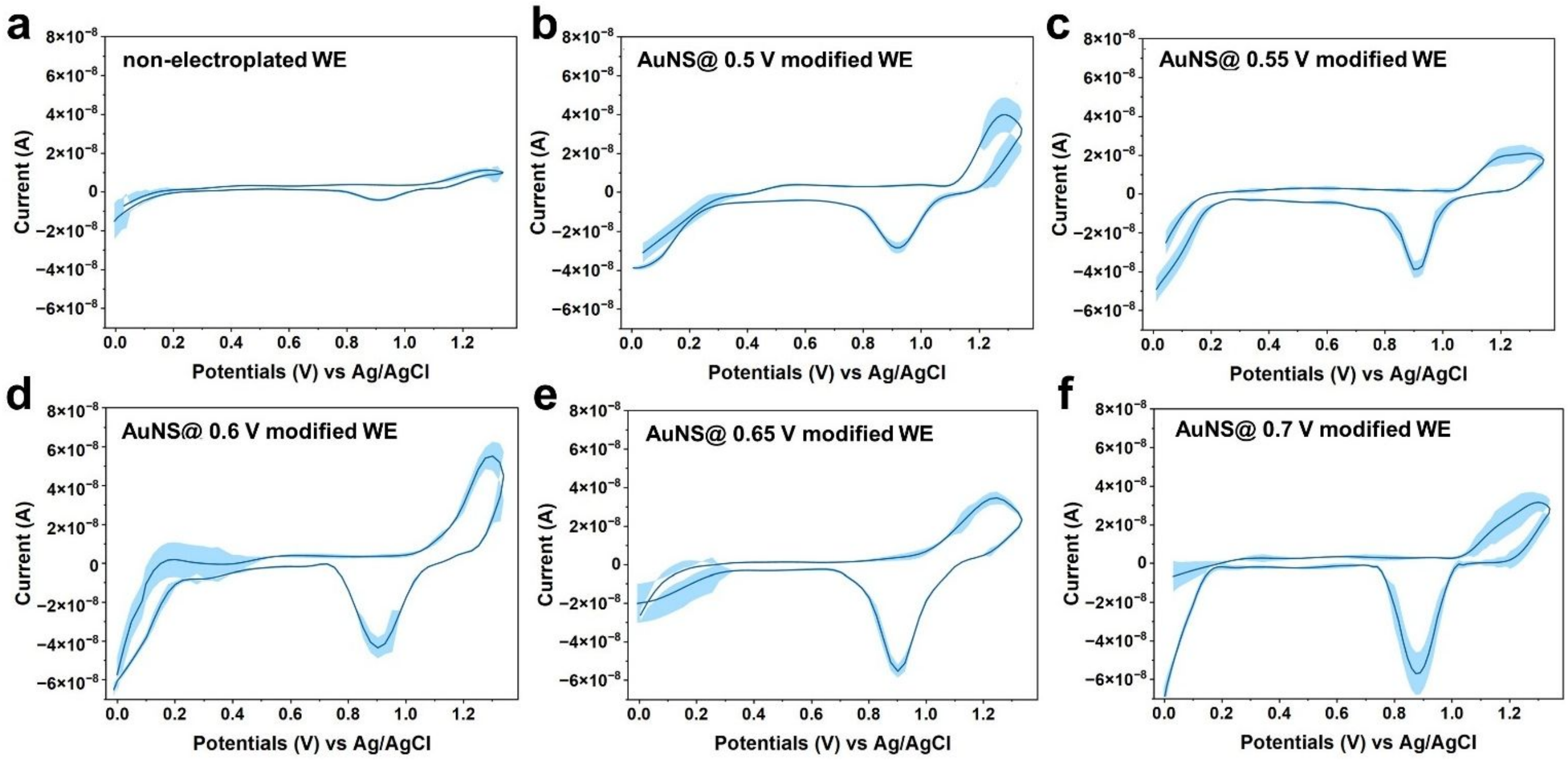

Figure S2. **Mean cyclic voltammograms of the WEs fabricated under different AuNS electrodeposition conditions**. Cyclic voltammograms recorded from (a) the non-electrodeposited bare gold WE (control) and WEs after chronoamperometric AuNS electrodeposition at (b) 0.50 V, (c) 0.55 V, (d) 0.60 V, (e) 0.65 V, and (f) 0.70 V *vs* Ag/AgCl. For each electrodeposition condition, measurements were performed using three independently fabricated neural probes, each containing two independent WEs (total *N* = 6 WEs). Data are presented as mean ± SD.

Eighteen neural probes (#S22~#S39) equally were divided into six fabrication groups consisting of non-electrodeposited WEs and WEs subjected to chronoamperometric AuNS electrodeposition at 0.50, 0.55, 0.60, 0.65, and 0.70 V *vs* Ag/AgCl for a fixed deposition time of 3 min. Each condition included three independently fabricated neural probes, with two independent WEs integrated on each probe, resulting in a total of *N* = 6 independent WEs per deposition condition. Each electrode was characterized independently by cyclic voltammetry, AFM, and optical microscopy.

Figure S2 presents the mean cyclic voltammograms (mean ± SD) obtained from all six WEs for each deposition condition. The corresponding ECSA values are summarized in **Table S2**. As the deposition potential increased from 0.50 to 0.70 V, the ECSA increased progressively from 2.93 × 10^3 ± 332 μm^2 to 5.39 × 10^3 ± 218 μm^2, corresponding to approximately 2.62- to 4.81-fold enhancement relative to the non-electrodeposited WE. Importantly, the electrodeposited electrodes exhibited relatively small device-to-device variability, with CV% values ranging from 2.02% to 11.32%, while the lowest ECSA variability was observed at 0.65 V (CV% = 2.02%), demonstrating excellent reproducibility of the electrodeposition process.

Table S2. ECSAs of WEs after chronoamperometric AuNS electrodeposition at different deposition potentials (0.50-0.70 V *vs* Ag/AgCl). The table summarizes the ECSA values (mean ± SD) together with the corresponding probe-to-probe CV%, calculated from six independently fabricated WEs (three independently

fabricated neural probes, each containing two independent WEs; total $N = 6$ WEs) for each electrodeposition condition. The normalized ECSA ratio ($\frac{ECSA_{E-coat}}{ECSA_{non-E-coat}}$) indicates fold enhancement relative to non-electroplated WEs.

Deposition voltage (V vs Ag/AgCl)	***ECSA (μm²)***	**CV%**	$\frac{ECSA_{E-coat}}{ECSA_{non-E-coat}}$
$WE_{non-E-coat}$	$1.12 \times 10^3 \pm 101$	8.95	1.00
**0.50**	$2.93 \times 10^3 \pm 332$	11.32	2.62
**0.55**	$3.28 \times 10^3 \pm 205$	6.26	2.93
**0.60**	$4.14 \times 10^3 \pm 121$	2.92	3.70
**0.65**	$5.25 \times 10^3 \pm 106$	2.02	4.69
**0.70**	$5.39 \times 10^3 \pm 218$	4.05	4.81

Note: Data represent mean ± SD (total $N = 6$ WEs from three independently fabricated neural probes, each comprising two separate three-electrode systems [two WEs per probe])

To directly correlate the electrochemical results with structural evolution, we have additionally incorporated atomic force microscopy (AFM, VK-X3000, Keyence Co., Itasca, IL, USA) characterization of the working electrodes before AuNS electrodeposition and after AuNS electrodeposition at 0.50, 0.55, 0.60, 0.65, and 0.70 V *vs* Ag/AgCl. Representative AFM topographic images and corresponding surface profiles are presented in **Figure S3**, providing qualitative visualization of the progressive evolution of the AuNS morphology with increasing electrodeposition potential. As the deposition potential increased, the WE exhibited progressively rougher and thicker surface features, whereas the Pt-coated CE maintained a relatively constant profile.

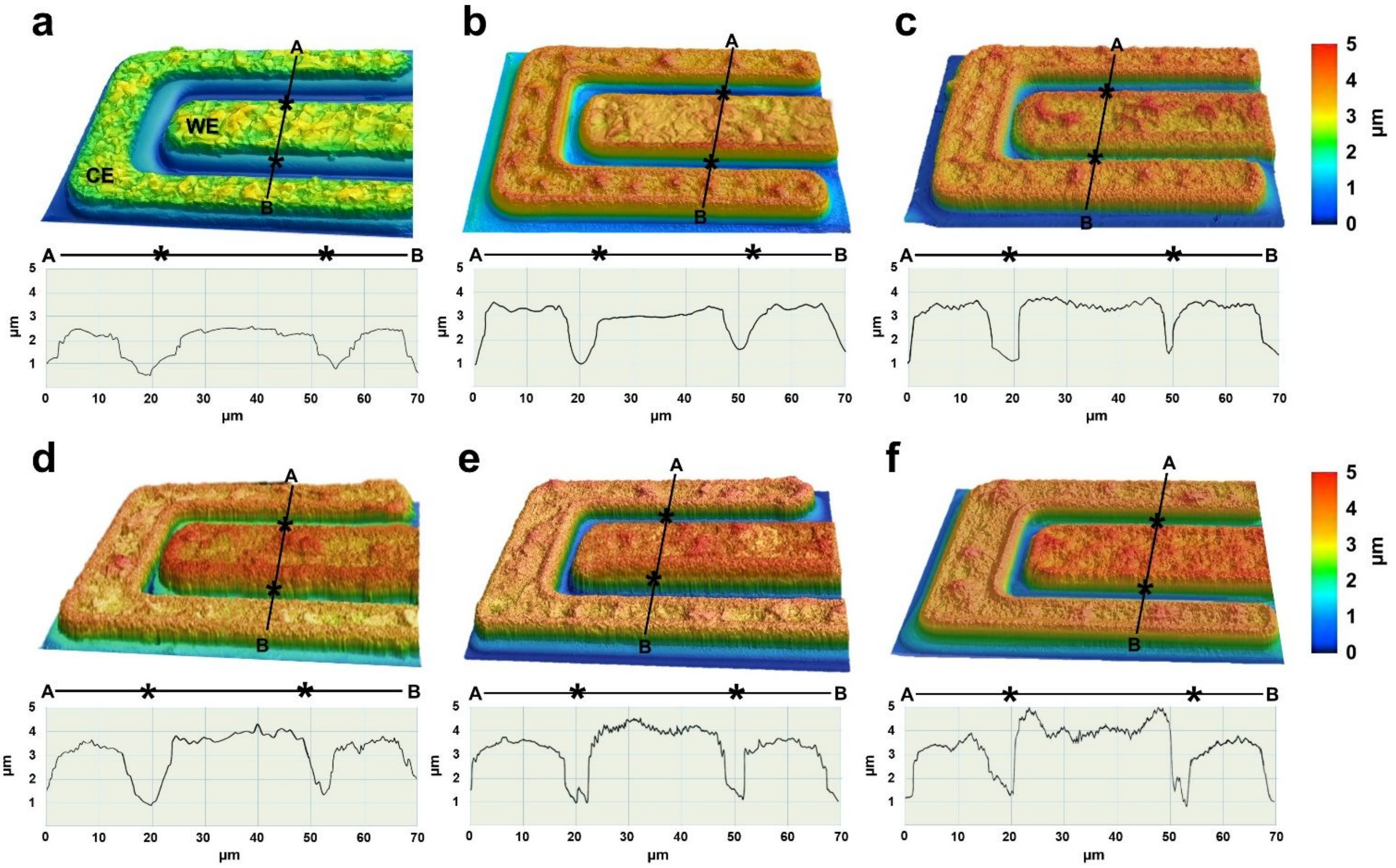

Figure S3. AFM characterization of the neural probe surface before and after AuNS electrodeposition. Representative three-dimensional AFM topographic images and corresponding surface profiles acquired along the line from A to B for (a) the non-electrodeposited bare gold control and after chronoamperometric AuNS electrodeposition at (b) 0.50 V, (c) 0.55 V, (d) 0.60 V, (e) 0.65 V, and (f) 0.70 V *vs* Ag/AgCl. For each electrodeposition condition, one representative WE was randomly selected from the two independent sensing units integrated within a neural probe for AFM visualization. Specifically, the left WE is shown in panels (a), (d), and (e), whereas the right WE is shown in panels (b), (c), and (f). In the control probe (a), neither the WE nor the CE was electrodeposited. In panels (b) through (f), Au nanostructures were electrodeposited onto the WE, whereas platinum was electrodeposited onto the CE. The surface profiles extend across both the CE and WE, and the region between the two asterisks (*) corresponds to the WE used for surface roughness analysis.

To quantitatively evaluate these structural changes, the arithmetic surface roughness (S_a) was determined for each electrodeposition condition, and the corresponding probe-to-probe reproducibility was evaluated using the CV%. The results are summarized in **Table S3**. The mean S_a increased from 0.176 ± 0.012 μm for the non-electrodeposited control to 0.524 ± 0.082 μm after electrodeposition at 0.70 V, while the corresponding CV% values remained within an acceptable range across independently fabricated neural probes. These results demonstrate that the observed morphological evolution was reproducible and not attributable to measurement variability. To further establish the relationship between structural evolution and electrochemical performance, **Figure S4** correlates the ECSA with the AFM-derived S_a. As the electrodeposition potential increased, ECSA increased concurrently with S_a, demonstrating a clear correspondence between electrochemical enhancement and surface roughening.

Table S3. **AFM characterization of surface roughness parameters and probe-to-probe reproducibility of WEs before and after AuNS electrodeposition.** S_a values are reported as mean ± SD together with the corresponding probe-to-probe CV% for the non-electrodeposited control and working electrodes after AuNS electrodeposition at 0.50, 0.55, 0.60, 0.65, and 0.70 V *vs* Ag/AgCl (N = 6 WEs for each condition).

Electrodeposition Condition	S_a (μm)	CV%
Control (Non-electrodeposited)	0.176 ± 0.012	6.82
0.50 V	0.301 ± 0.044	14.72
0.55 V	0.414 ± 0.035	8.50
0.60 V	0.448 ± 0.033	7.37
0.65 V	0.516 ± 0.021	4.09
0.70 V	0.524 ± 0.082	15.56

Note: Data represent mean ± SD (total *N* = 6 WEs from three independently fabricated neural probes, each comprising two separate three-electrode systems [two WEs per probe])

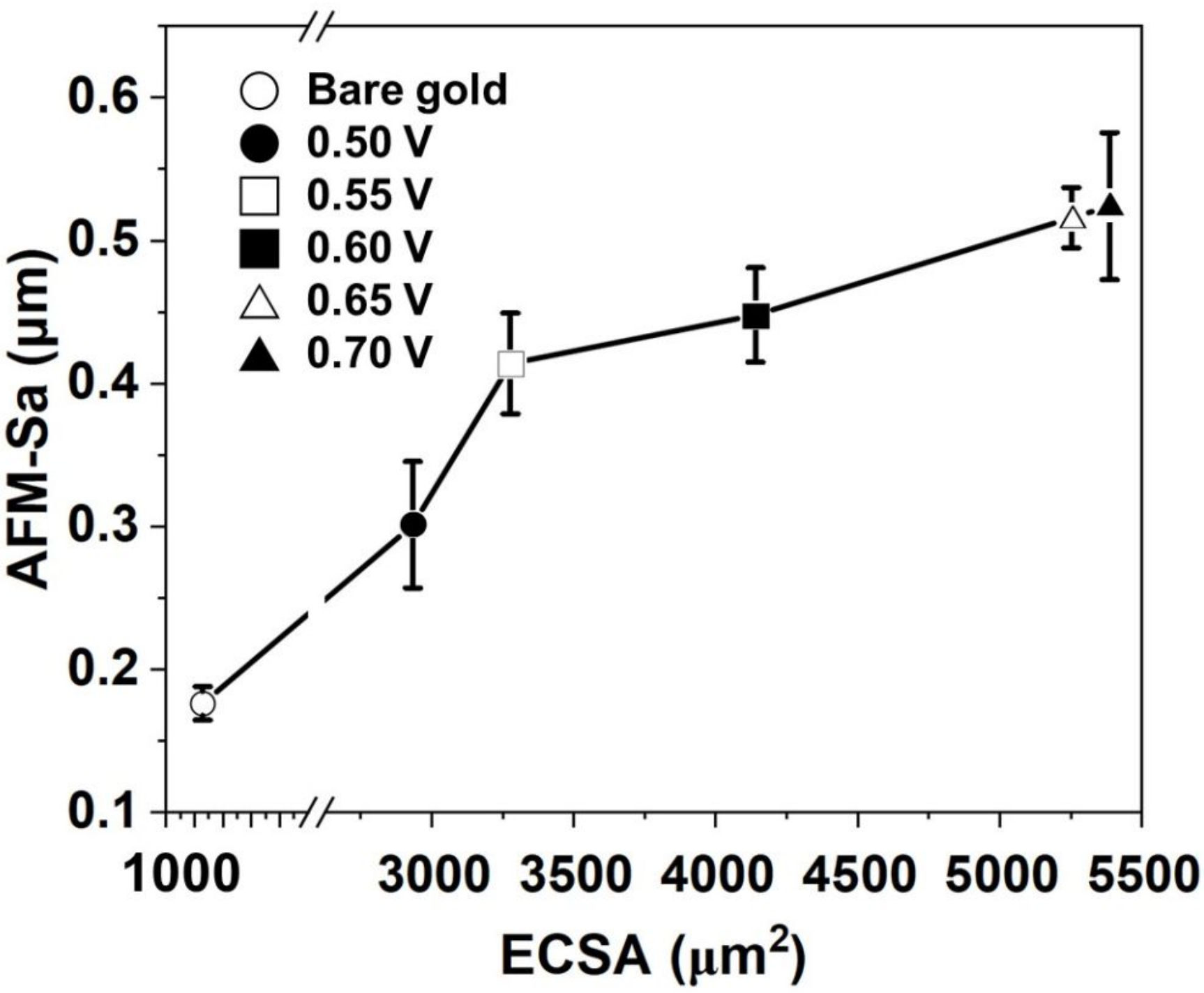

Figure S4. Relationship between the ECSA and AFM-derived S_a of WEs fabricated under different AuNS electrodeposition potentials. Data are presented as mean ± SD for the non-electrodeposited bare gold control and working electrodes after chronoamperometric AuNS electrodeposition at 0.50, 0.55, 0.60, 0.65, and 0.70 V *vs* Ag/AgCl. The results show that increases in ECSA are accompanied by corresponding increases in S_a, supporting that the enhanced electrochemical performance is associated with systematic changes in AuNS surface morphology across different electrodeposition conditions.

Beyond the electrochemical and structural characterization, we further evaluated the practical manufacturability of the electrodeposition process. As shown in **Figure S5**, optical microscopy revealed a clear difference in fabrication robustness between the two highest deposition potentials. Although electrodeposition at 0.70 V produced the largest average ECSA, it also frequently resulted in excessive lateral growth of the AuNS layer beyond the intended working electrode boundary. This AuNS overflow occasionally bridged adjacent metal traces or neighboring electrodes, thereby increasing the risk of electrical short-circuiting during device fabrication (**Figure S5**, left). In contrast, electrodeposition at 0.65 V produced well-confined AuNS growth that remained within the designed working electrode geometry without observable overflow (**Figure S5**, right), thereby preserving electrical isolation between adjacent electrodes while maintaining an ECSA comparable to that obtained at 0.70 V. Collectively, these results indicate that 0.65 V provides the most appropriate electrodeposition condition by balancing electrochemical enhancement, structural reproducibility, and fabrication reliability.

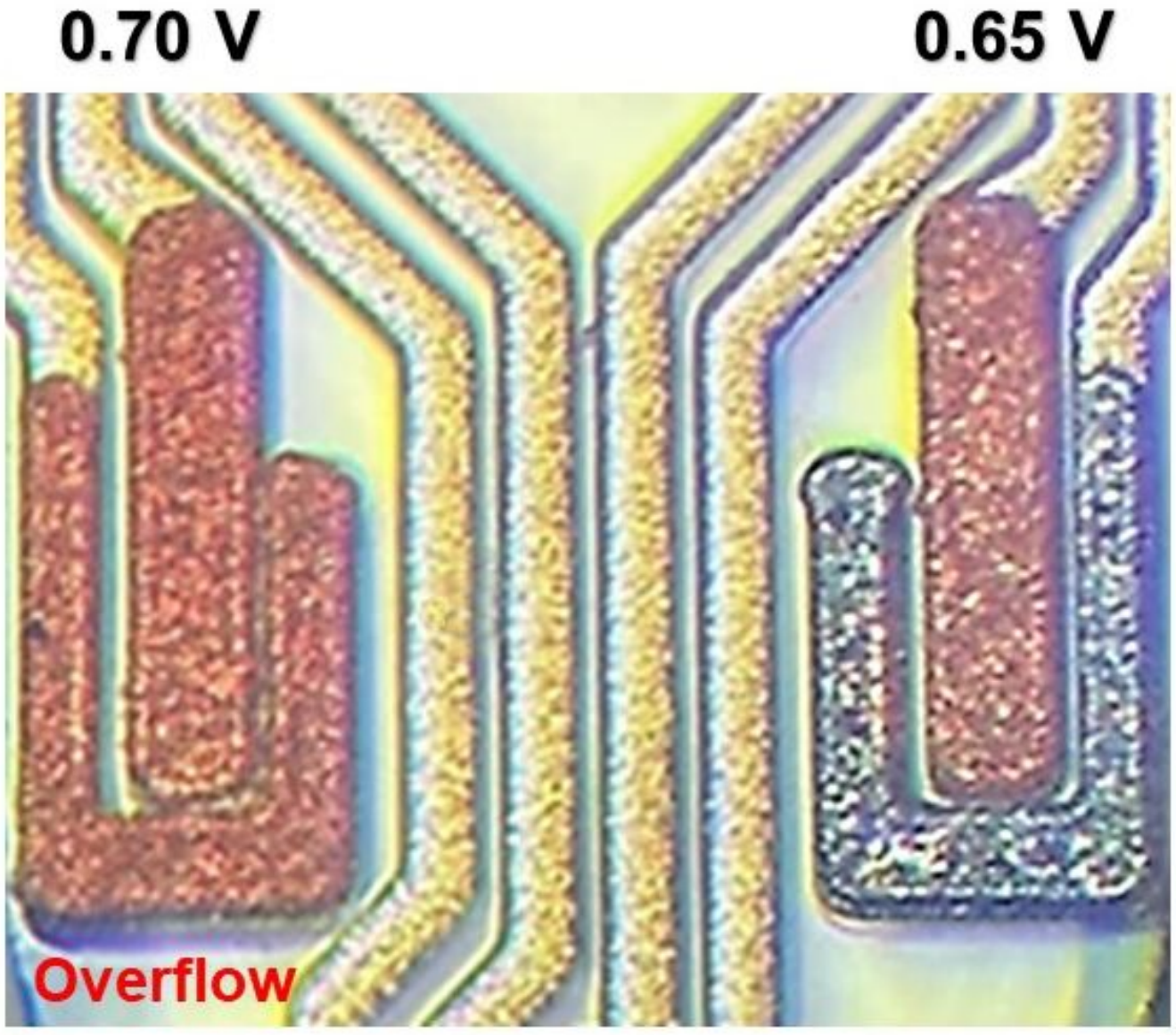

Figure S5. Optical microscopy comparison of WEs after AuNS electrodeposition at 0.65 and 0.70 V *vs* Ag/AgCl. Electrodeposition at 0.70 V (left) frequently produced excessive lateral growth of the AuNS layer beyond the designed WE boundary, resulting in overflow toward the adjacent CE. Consequently, the overflowing AuNS electrically bridged the WE and CE, preventing selective Pt electrodeposition on the CE and causing unintended Au deposition on the CE surface (gold-brown region), thereby increasing the risk of electrical short circuits within the integrated three-electrode architecture. In contrast, electrodeposition at 0.65 V (right) generated a well-confined AuNS layer that substantially increased the WE surface area while maintaining clear electrical isolation from the adjacent CE. This enabled subsequent selective Pt electrodeposition on the CE (gray-black region) without interference from AuNS overflow. These observations demonstrate that electrodeposition at 0.65 V provides the optimal balance between electrochemical enhancement, fabrication robustness, and reliable integration of the on-chip three-electrode system.

Note 3. SEM and EDS characterization of electrodeposited WE, RE, and CE of the microelectrode array probe

SEM was employed to examine the surface morphology of the electrodeposited WE, RE, and CE of the microelectrode array probe. Elemental composition and spatial distribution were analyzed using energy-dispersive X-ray spectroscopy (EDS) integrated within the SEM system. During EDS measurements, a focused electron beam was directed onto the electrode surfaces, and characteristic X-ray emissions from constituent elements were collected to generate elemental spectra and corresponding mapping images. Elemental maps of Au, Ag, Cl, and Pt were acquired to verify electrode-specific surface modification and to confirm the absence of unintended material deposition.

Figure S6 shows SEM and EDS characterization of the WE after electrodeposition of AuNS at a deposition potential of 0.65 V. Low-magnification SEM imaging (**Figure S6a**, 1500×) reveals uniform coverage of nanostructured deposits across the WE surface, while high-magnification imaging (**Figure S6b**, 10,000×) shows that the deposited AuNS exhibit fine, dendritic-like nanostructures with pronounced surface roughness. Such dendritic morphology is consistent with high nucleation density and is expected to provide a large specific surface area, supporting the substantial increase in electrochemically active surface area observed at this deposition condition. EDS analysis (**Figure S6c**) indicates a dominant Au signal with a volume percentage of approximately 86.8%, confirming that the nanostructures formed on the WE surface are composed primarily of gold.

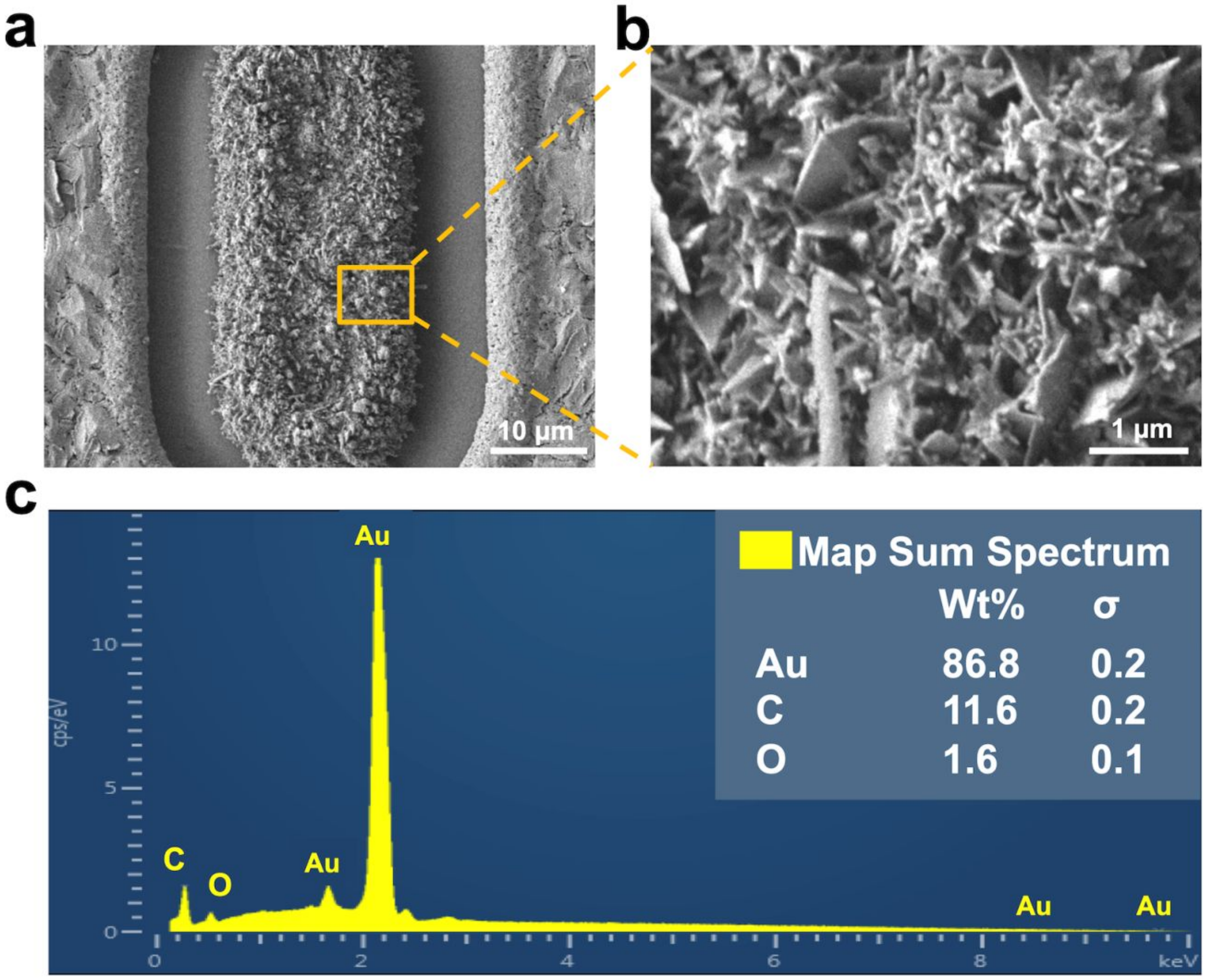

Figure S6. SEM and EDS characterization of AuNS-modified WE of the neural probe. (a) Low-magnification SEM image (1500×) of the WE after chronoamperometric electrodeposition of AuNS at 0.65 V (*vs* Ag/AgCl). (b) High-magnification SEM image (10,000×) showing fine dendritic-like AuNS with pronounced surface roughness. (c) Corresponding EDS spectrum confirming dominant Au composition (86.8 vol %), verifying successful gold electrodeposition on the WE.

Figure S7 presents SEM and EDS characterization of the RE after silver electrodeposition followed by chloridation to form an Ag/AgCl reference electrode. SEM images (**Figure S7a, b**) show the formation of relatively large, coral-like Ag/AgCl structures on the RE surface. EDS elemental analysis (**Figure S7c**) reveals prominent Ag and Cl signals with volume percentages of approximately 76.4% and 13.2%, respectively, verifying successful conversion of the silver layer into Ag/AgCl and confirming the intended chemical composition of the RE.

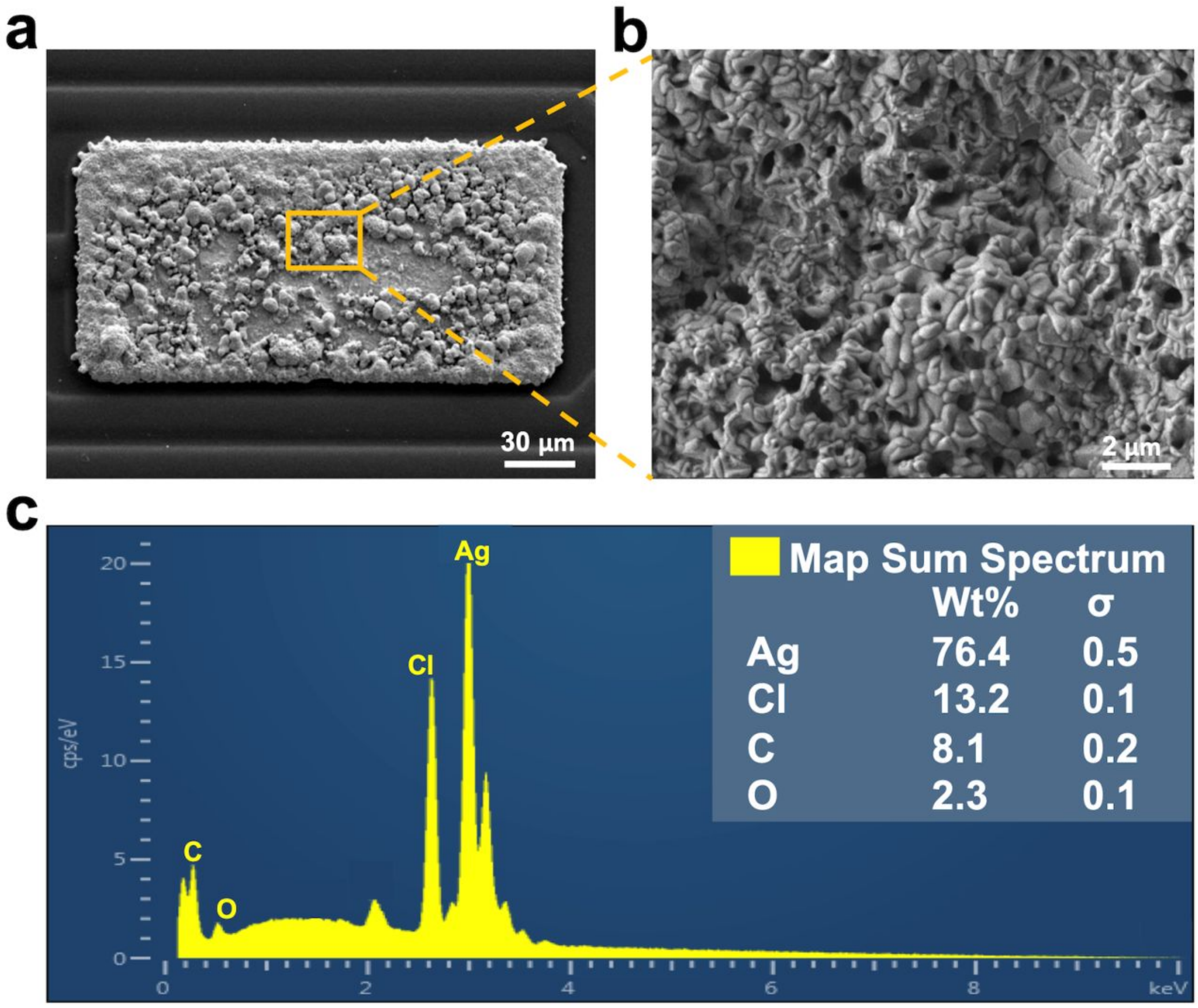

Figure S7. SEM and EDS characterization of Ag/AgCl-modified RE of the neural probe. (a) SEM image (500×) of the RE after silver electrodeposition followed by chloridation, showing formation of Ag/AgCl structures on the electrode surface. (b) High-magnification SEM image (8000×) revealing coral-like Ag/AgCl morphology. (c) Corresponding EDS spectrum displaying prominent Ag (76.4 vol %) and Cl (13.2 vol %) signals, confirming formation of Ag/AgCl on the RE.

Figure S8 shows SEM and EDS characterization of the CE after platinum electrodeposition. SEM images (**Figure S8a, b**) demonstrate that a relatively smooth and continuous platinum nanostructured layer is formed on the CE surface. Corresponding EDS analysis (**Figure S8c**) indicates a strong Pt signal with a volume percentage of approximately 90.1%, confirming that the CE surface is predominantly covered with platinum.

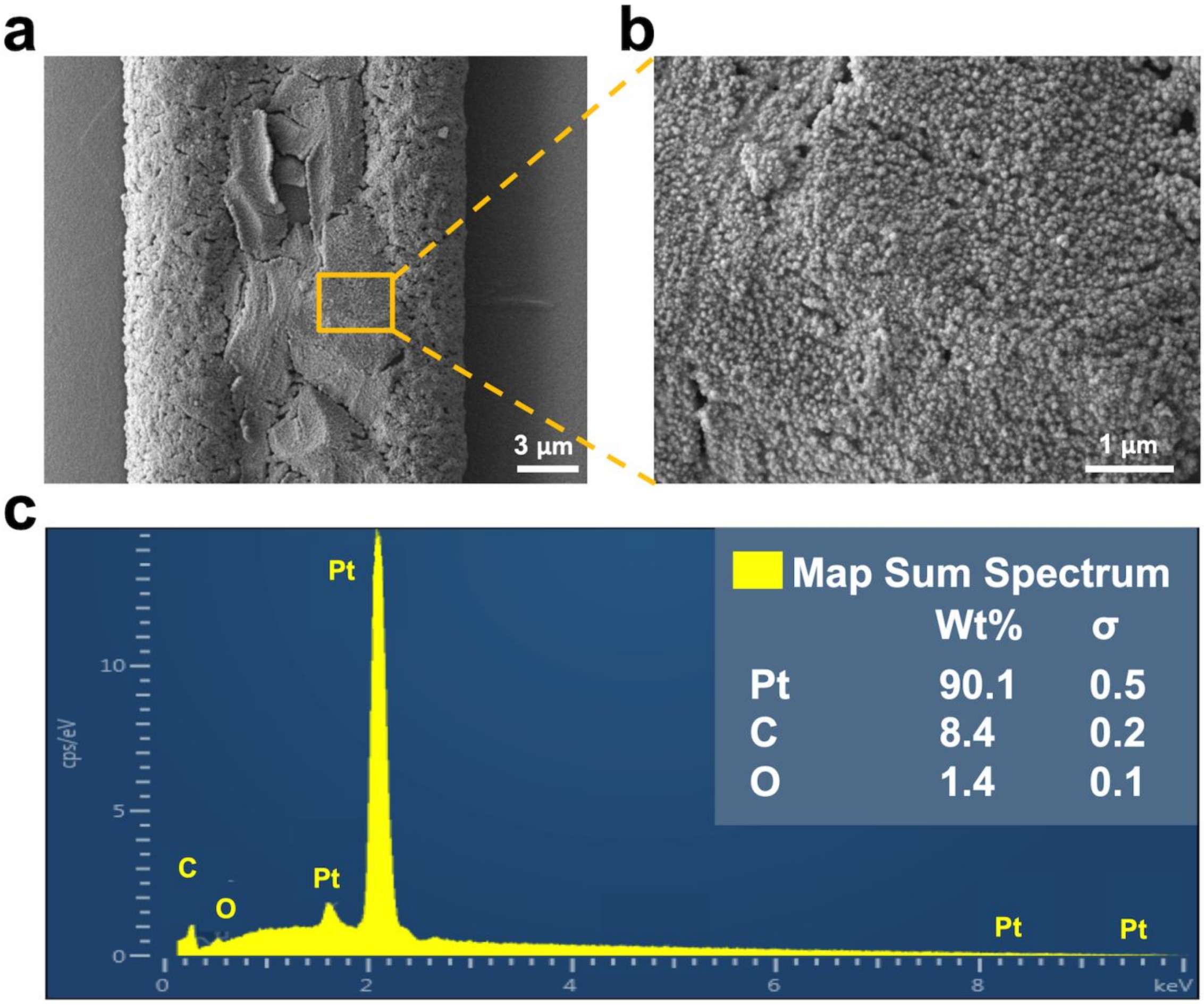

Figure S8. SEM and EDS characterization of Pt-modified CE of the neural probe. (a) SEM image (4500×) of the CE after platinum electrodeposition, showing uniform coverage of a nanostructured platinum layer. (b) High-magnification SEM image (20,000×) revealing a relatively smooth and continuous platinum nanostructured morphology. (c) Corresponding EDS spectrum indicating dominant Pt composition (90.1 vol %), confirming successful platinum deposition on the CE.

Note 4. Evaluate the intrinsic electrochemical stability of the integrated on-chip Ag/AgCl RE

We performed OCP measurements to evaluate the intrinsic electrochemical stability of the integrated on-chip Ag/AgCl RE under physiologically relevant conditions[3]. Because OCP directly reflects the equilibrium potential of a reference electrode under zero-current conditions, it provides a fundamental metric for assessing intrinsic electrode stability[3]. The experimental configuration is illustrated in **Figure S9**.

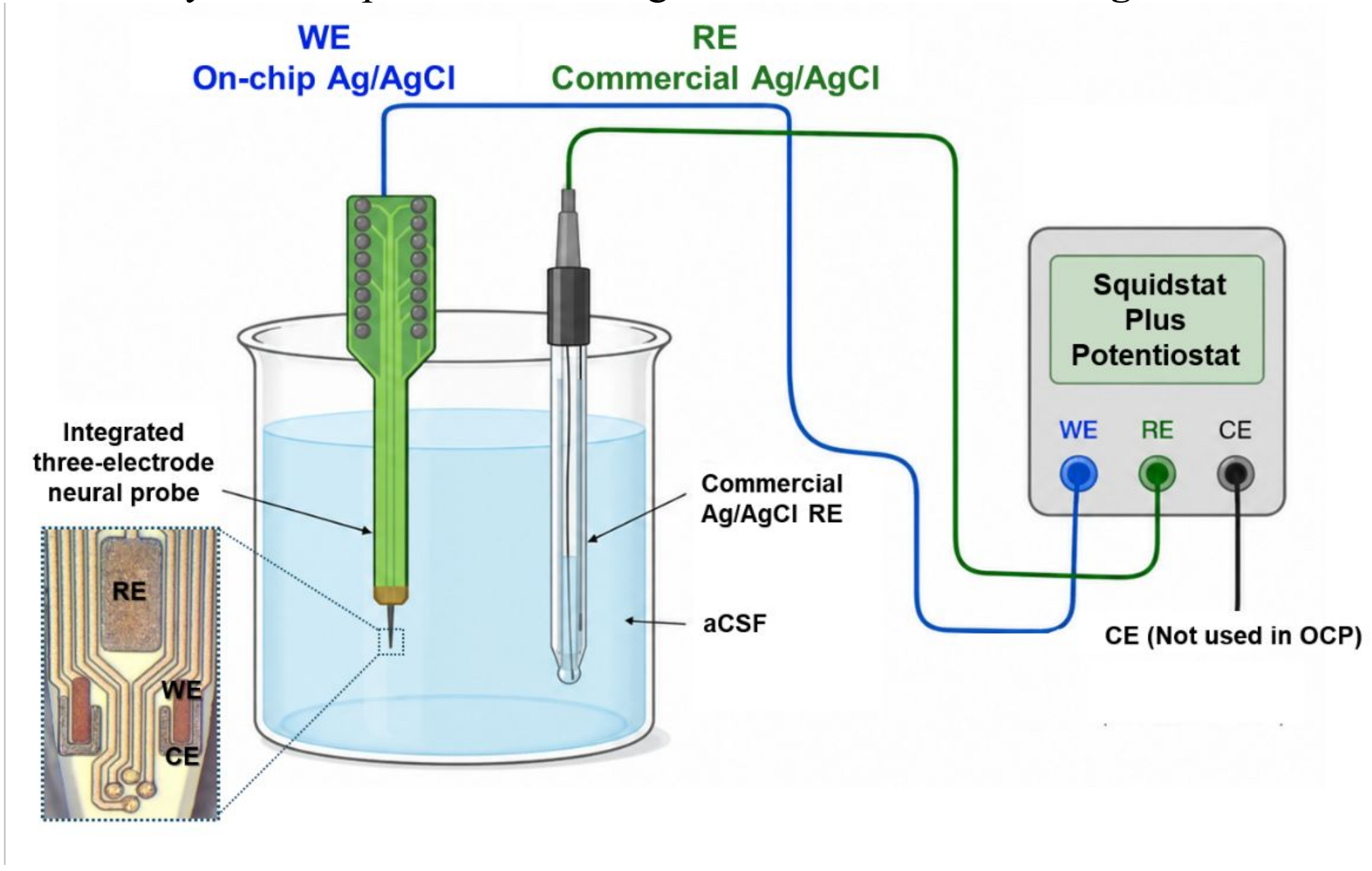

Figure S9. Schematic illustration of the OCP measurement setup. The on-chip Ag/AgCl electrode integrated within the neural probe was used as the WE, while a commercial SCE served as the RE. Both electrodes were immersed in aCSF. The potential difference between on-chip Ag/AgCl electrode and saturated calomel electrode was recorded using a potentiostat with no current flow during measurement.

The resulting OCP profile is presented in **Figure S10**. The potential evolution can be divided into two characteristic regions. During the initial equilibration period (0-600 sec), the measured potential gradually decreased by approximately 2.44 mV, corresponding to an average drift rate of approximately 0.24 mV min^{-1}. This transient behavior is consistent with interfacial equilibration processes, including stabilization of the Ag/AgCl layer and establishment of electrochemical equilibrium between the electrode surface and the surrounding electrolyte[4, 5]. Such initial potential relaxation is commonly observed in microfabricated or pseudo-reference electrodes and should not be interpreted as evidence of electrode instability. This behavior is also consistent with the transient equilibration previously reported for integrated Ag/AgCl reference electrodes[6].

Following the initial equilibration period (>600 sec), the potential reached a steady-state regime at approximately 46.7 mV and remained highly stable throughout the remainder of the measurement. Over the subsequent ~50 min, the potential fluctuation remained below 0.1 mV, demonstrating excellent short-term electrochemical stability. This level of stability is comparable to, or better than, that reported for similar microfabricated Ag/AgCl reference electrodes [5, 7].

In addition to temporal stability, a constant potential offset of approximately 48 mV relative to the commercial SCE was observed. This offset primarily originates from differences in chloride ion activity between the integrated Ag/AgCl RE and the commercial SCE, as described by the Nernst equation. Specifically, the commercial SCE maintains a fixed internal KCl concentration, whereas the integrated Ag/AgCl electrode directly interfaces with the external electrolyte (aCSF), resulting in a systematic shift in equilibrium potential. Minor contributions from liquid-junction potentials and fabrication-related variations may also contribute to the measured offset. Importantly, once equilibrium was established, this offset remained essentially constant throughout the measurement and therefore did not introduce additional temporal drift. Such stable offset behavior is generally considered acceptable for integrated on-chip Ag/AgCl reference electrodes[7].

With respect to electrochemical sensing performance, both the magnitude of the initial drift and the presence of a stable potential offset are unlikely to significantly influence measurements over the experimental timescale investigated in this work. The observed drift rate remained sufficiently low to provide stable electrochemical control during SWV. Furthermore, because the analytical signal throughout this study was

defined as the change in current relative to the baseline (I_{diff}) rather than the absolute current, slow common-mode baseline shifts arising from reference potential offset or minor temporal drift are inherently compensated during differential signal analysis[8]. Consequently, the integrated on-chip Ag/AgCl reference electrode provides stable and reliable electrochemical behavior over the timescale relevant to the acute *in vitro* and *in vivo* sensing experiments reported in this study.

It should be noted that the present OCP measurements were intended to evaluate the short-term stability of the integrated Ag/AgCl RE under acute operating conditions. Long-term potential drift and electrochemical stability during chronic implantation remain important considerations but fall beyond the scope of the present proof-of-concept study.

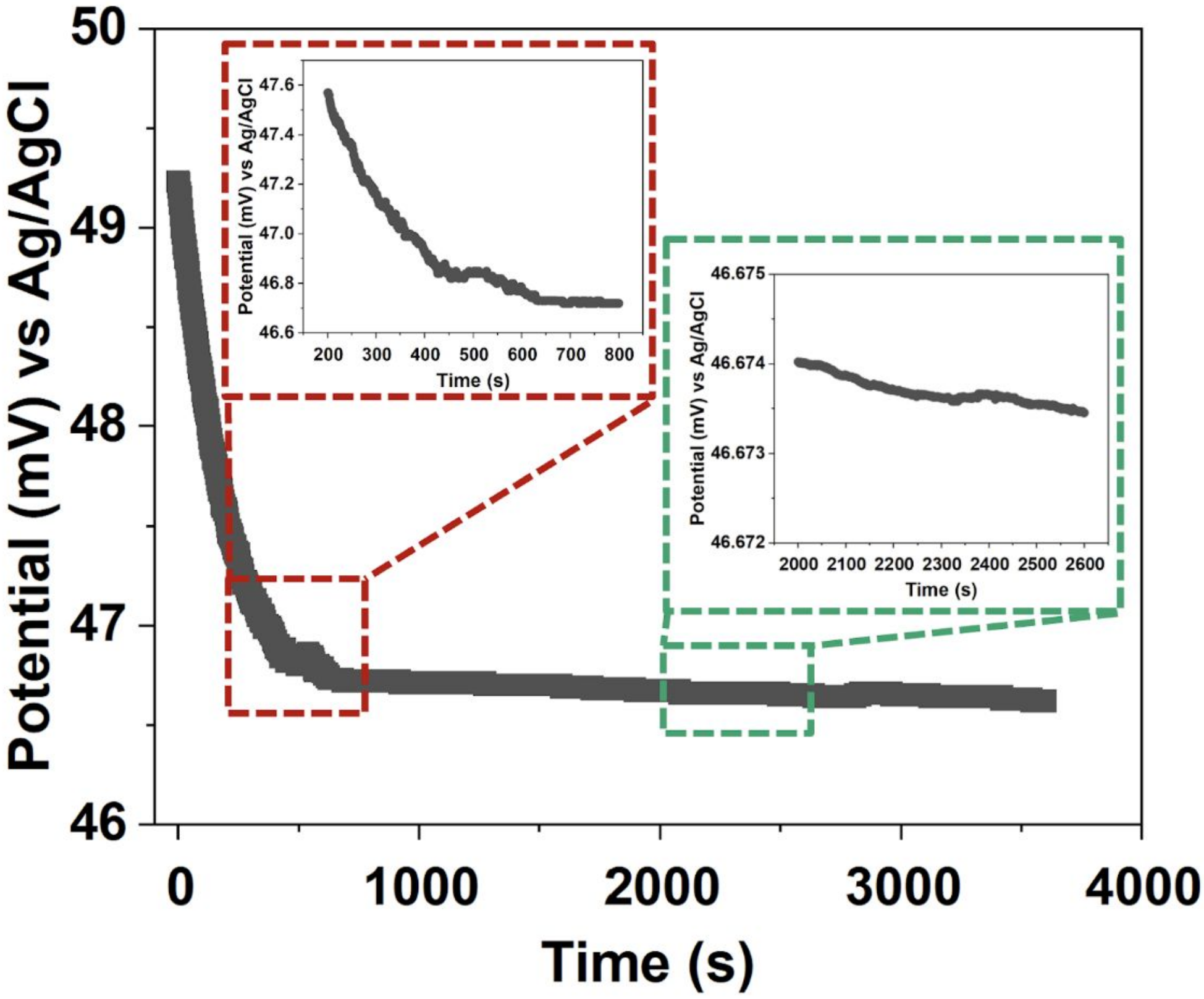

Figure S10. OCP stability of the on-chip Ag/AgCl RE in aCSF. The OCP exhibits a rapid initial decrease within the first 600 sec, followed by a stable plateau, indicating a transition from interfacial equilibration to steady-state behavior. The red dashed region (200-800 sec) highlights the initial equilibration phase, with the inset showing a magnified view of the transient potential decay and fluctuation. The green dashed region (2,000-2,600 sec) corresponds to the steady-state regime, where the inset demonstrates minimal potential variation (<0.1 mV) over an extended period, confirming excellent short-term stability of our integrated on-chip RE.

Note 5. System-specific validation of the SWV interrogation frequency for the AuNS-modified electrochemical aptamer sensing interface

To verify that the selected SWV interrogation frequency was appropriate for the present electrochemical aptamer sensing interface, a frequency-dependent validation experiment was performed during the development of the sensing platform. Unlike the SWV amplitude and step potential, which were adopted from operating conditions widely established for MB-based electrochemical aptamer sensors, the interrogation frequency was experimentally evaluated because it depends on the apparent electron-transfer kinetics of the specific sensing interface and therefore cannot be assumed to be universally applicable.

Throughout the optimization study, the SWV amplitude (25 mV) and step potential (5 mV) were maintained constant so that only the effect of interrogation frequency was evaluated. SWV measurements were performed at 10, 20, 50, 100, 150, and 200 Hz using 1 nM DA together with DA-free aCSF under identical experimental conditions. Each interrogation frequency was independently evaluated using three fabricated neural probes, resulting in a total of 18 neural probes (Neural Probe #S1-#S18) for the complete frequency-validation study.

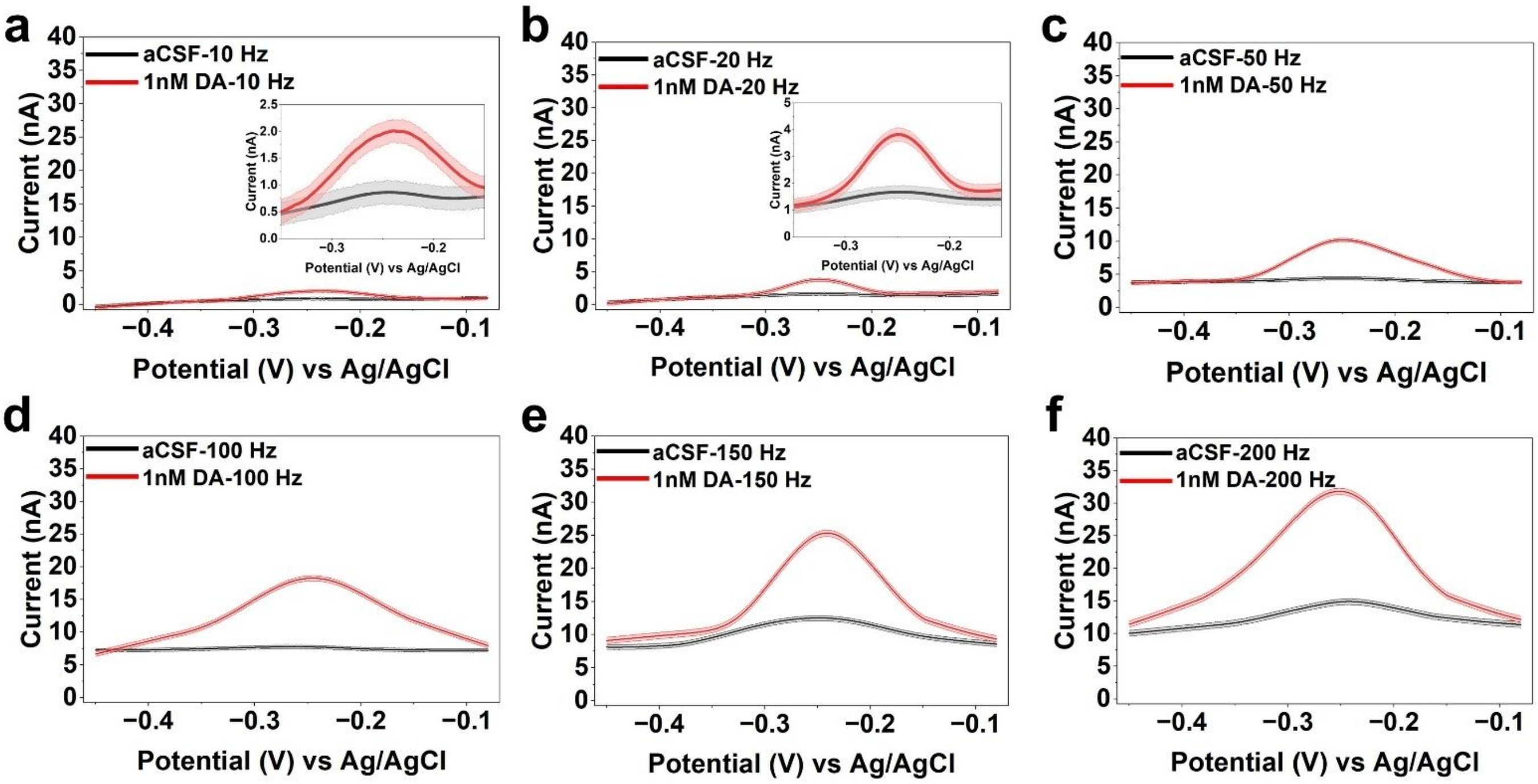

Figure S11. **Mean SWV voltammograms used for system-specific validation of the SWV interrogation frequency.** Mean SWV voltammograms recorded in DA-free aCSF (black) and 1 nM DA (red) at interrogation frequencies of (a) 10 Hz, (b) 20 Hz, (c) 50 Hz, (d) 100 Hz, (e) 150 Hz, and (f) 200 Hz. The SWV amplitude (25 mV), step potential (5 mV), and potential window (0 to -0.5 V) were maintained constant throughout the experiment so that only the influence of interrogation frequency was evaluated. Insets in panels (a) and (b) show enlarged views of the SWV oxidation peak to facilitate visualization of the relatively small faradaic responses obtained at low interrogation frequencies. Measurements were obtained from three independently fabricated neural probes, each containing two independent electrochemical sensing units (total $N = 6$ sensing units). For each sensing unit, SWV measurements at each interrogation frequency were repeated three times, and the data are presented as mean ± SD. As the interrogation frequency increased, both the faradaic peak current and the background peak current increased progressively.

The resulting mean SWV voltammograms are presented in **Figure S11**. The peak currents obtained from the DA-containing solution (I_f^{DA}) and the corresponding DA-free aCSF blank (I_f^{blank}) were extracted for each interrogation frequency. Two analytical parameters were subsequently calculated to evaluate the frequency-dependent sensing performance. The first parameter was the absolute faradaic current change (ΔI_f), calculated as:

$$\Delta I_f = I_f^{DA} - I_f^{blank}$$

which represents the absolute increase in electrochemical signal induced by DA binding. Because increasing the interrogation frequency also increased the background current, a second parameter, the normalized faradaic signal change ($\Delta I_f^{DA}(\%)$), was calculated according to:

$$\Delta I_f^{DA}(\%) = \frac{\Delta I_f}{I_f^{blank}} \times 100\%$$

This normalization compensates for the frequency-dependent increase in background current and enables direct comparison of the analytical signal enhancement across different interrogation frequencies.

The corresponding quantitative analyses are summarized in **Figure S12**. As the interrogation frequency increased, both the DA response current and the background current increased progressively, resulting in a continuous increase in the absolute current difference (ΔI_f) as shown in **Figure S12a**. However, the $\Delta I_f^{DA}(\%)$ increased from 10 Hz to 100 Hz, reached a maximum at 100 Hz, and subsequently decreased at 150 Hz and 200 Hz. These results indicate that although higher interrogation frequencies continued to increase the absolute faradaic response, the accompanying increase in background current became increasingly significant, thereby reducing the relative analytical signal enhancement at higher frequencies.

Our results demonstrate that 100 Hz provides the most favorable balance between absolute signal intensity, background current, normalized faradaic signal change, and measurement reproducibility for the present AuNS-modified electrochemical aptamer sensing interface of neural probe. Accordingly, 100 Hz was selected as the SWV interrogation frequency for all subsequent electrochemical characterization performed in this study.

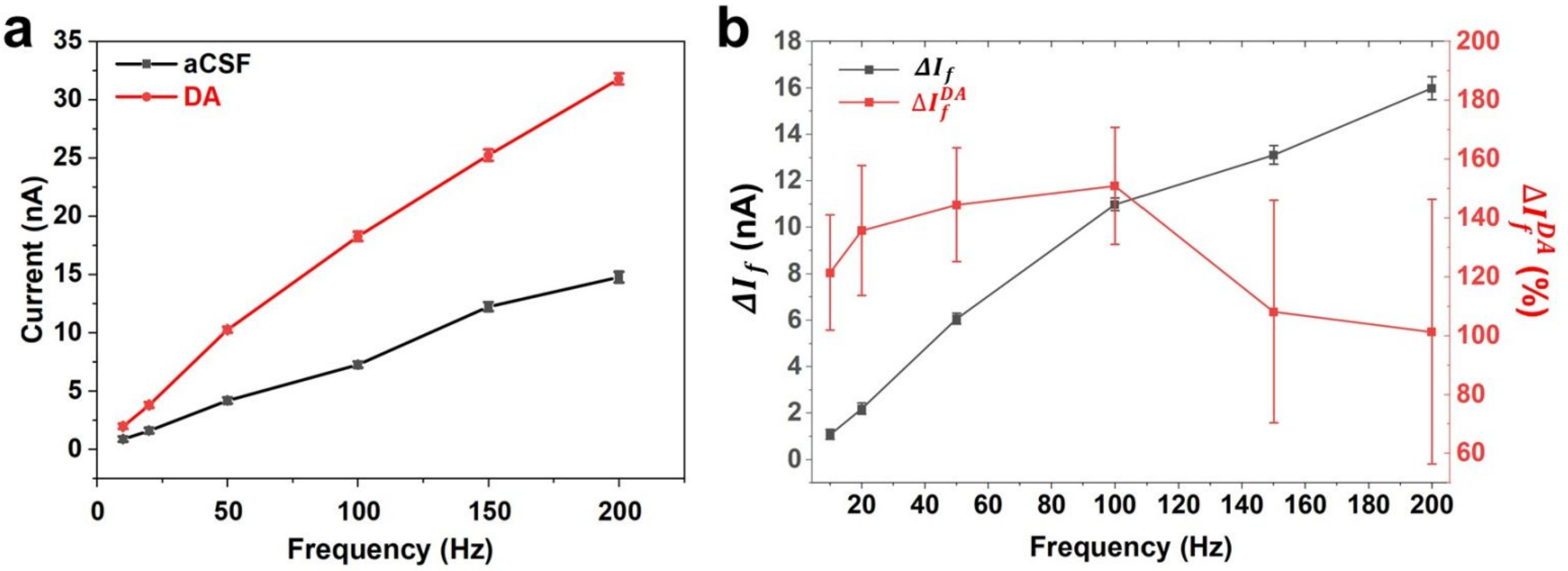

Figure S12. **System-specific validation of the SWV interrogation frequency for the AuNS-modified electrochemical aptamer sensing platform**. (a) Frequency-dependent SWV peak currents measured in DA-free aCSF (black) and 1 nM DA solution (red) at interrogation frequencies of 10, 20, 50, 100, 150, and 200 Hz while maintaining a constant SWV amplitude of 25 mV and step potential of 5 mV. (b) Corresponding absolute current change (ΔI_f, black) and normalized faradaic signal change ($\Delta I_f^{DA}(\%)$, red) as a function of interrogation frequency. The $\Delta I_f^{DA}(\%)$ reached a maximum at 100 Hz, indicating the optimal balance between signal enhancement and background current for the present AuNS-modified sensing interface of neural probe. Measurements were obtained from three independently fabricated neural probes, each containing two independent sensing units (total N = 6 sensing units). For each sensing unit, SWV measurements at each interrogation frequency were repeated three times. Data are presented as mean ± SD.

Note 6. Design rationale of the scrambled aptamer control

We performed the suggested scrambled aptamer control experiment under identical experimental conditions. The functional DA aptamer measurements were obtained using the same three independently fabricated neural probes employed in the SWV frequency-validation study described in ***Supplementary Note 5*** (Neural Probes #S10-#S12), whereas an additional three independently fabricated batch-matched neural probes (Neural Probes #S19-#S21) were functionalized with the scrambled DNA sequence to serve as the control group. Each neural probe contained two independent electrochemical sensing units, yielding a total of $N = 6$ sensing units for both the functional and scrambled groups.

All electrochemical measurements were performed using the experimentally validated SWV interrogation parameters (frequency = 100 Hz, amplitude = 25 mV, step potential = 5 mV, and potential window = 0 to -0.5 V). Notably, the interrogation frequency of 100 Hz corresponded to the optimal operating condition identified in the frequency-validation study presented in ***Supplementary Note 5***, where systematic evaluation over 10-200 Hz demonstrated that this frequency provided the most appropriate balance between signal intensity, background current, normalized analytical response, and measurement reproducibility for the present AuNS-modified sensing platform.

To ensure that the control experiment isolated the contribution of sequence-specific molecular recognition, the scrambled DNA probe was designed as summarized in **Table S4**. The scrambled probe retained the same thiol anchor, sequence length, nucleotide composition, GC content, internal MB labeling position, immobilization chemistry, and 6-MCH backfilling procedure as the functional DA aptamer. The DA-recognition sequence was the only intentionally modified feature, whereas all other structural, electrochemical, and experimental parameters were maintained unchanged. Consequently, any difference in electrochemical response between the functional DA aptamer sensor and the scrambled aptamer control can be attributed primarily to the presence or absence of an intact DA-recognition sequence rather than to differences in probe fabrication or measurement conditions.

Table S4. The scrambled aptamer was designed to retain the same sequence length, nucleotide composition, GC content, thiol anchor, MB labeling position, and surface immobilization chemistry as the functional DA-specific aptamer. The DA-recognition sequence was deliberately disrupted while all other structural and electrochemical parameters were preserved. This design ensures that the integrity of the DA-recognition sequence was the only intentionally varied experimental factor between the functional DA aptamer sensor and the scrambled aptamer control.

Component	Functional DA Aptamer Sequence (5’ - 3’)	Scrambled Control Sequence (5’ - 3’)	Experimental Control
**5′ Thiol Anchor**	HO-C6-S-S-C6	HO-C6-S-S-C6	Identical thiol self-assembled monolayer chemistry for Au immobilization
**Pre-MB Sequence (19 nt)**	CGA CGC CAG TTT GAA GGT T	GGT CAT GCT GTA CGT ACG A	Same sequence length (19 nt) and identical nucleotide composition
**Redox Reporter**	MB (between 19th and 20th nt)	MB (same position)	Identical methylene blue labeling position and electron-transfer reporter
**Post-MB Sequence (25 nt)**	CG TTC GCA GGT GTG GAG TGA CGT CG	GGC TTG TGC GGT AGC TGG TAC GGC A	Same sequence length (25 nt) and identical nucleotide composition
**Total Core Sequence**	44-mer (68.2% GC content)	44-mer (68.2% GC content)	Identical overall sequence length and GC content
**DA-Recognition Motif**	Intact	Deliberately disrupted by sequence scrambling	**Only intentionally modified feature between the two sensing interfaces**

Full scrambled sequence: 5’-HO-C6-S-S-C6-GGT CAT GCT GTA CGT ACG A-MB-GGC TTG TGC GGT AGC TGG TAC GGC A-3’

As shown in **Figure S13a**, the scrambled aptamer exhibited nearly identical SWV voltammograms in DA-free aCSF and after exposure to 1 nM DA. The enlarged inset further illustrates that the MB oxidation peak remained essentially unchanged following DA addition. Quantitative analysis summarized in **Table S5** showed that the mean peak current changed only from 7.7225 ± 0.2113 nA to 7.8449 ± 0.2256 nA, corresponding to a net current increase of 0.1224 nA, which remained well within the experimentally

determined baseline (aCSF) variation ($3\sigma_{blank}$ = 0.6339 nA). Therefore, no statistically distinguishable electrochemical response was observed in the absence of the intact DA-recognition sequence.

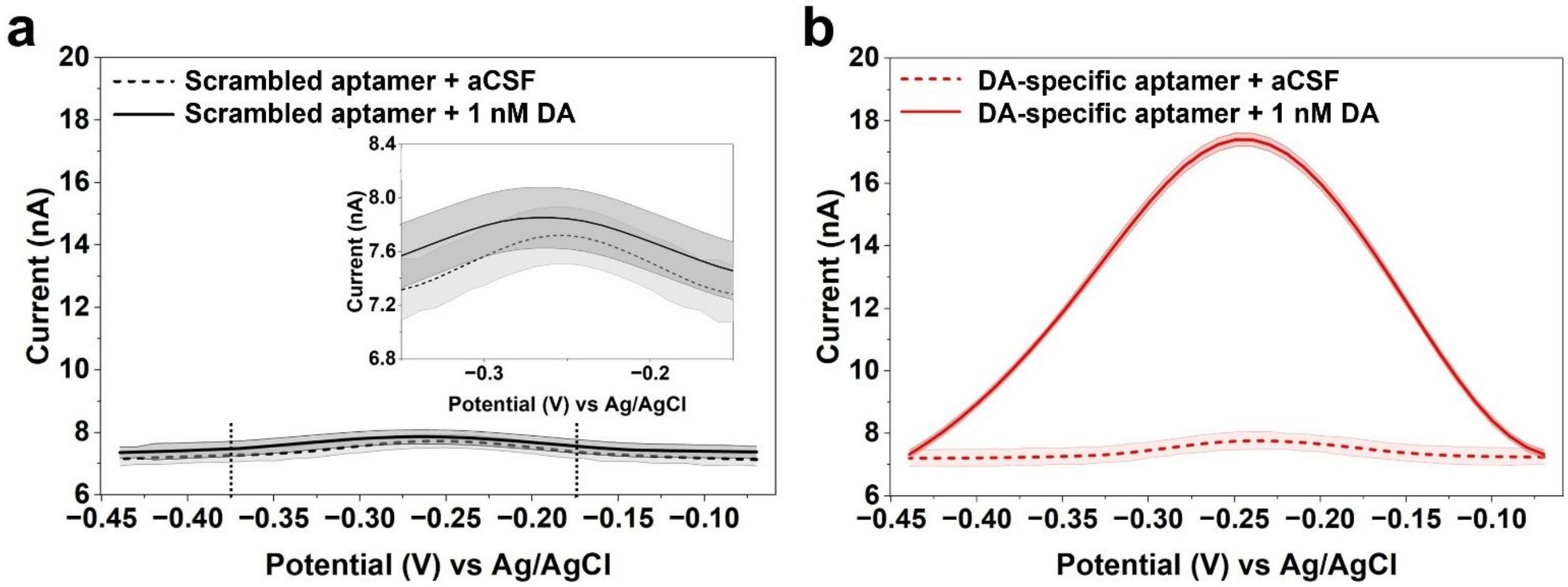

Figure S13. **Scrambled aptamer control validates sequence-specific DA recognition**. Mean SWV voltammograms obtained from the (a) scrambled aptamer control and (b) functional DA-specific aptamer in DA-free aCSF and after exposure to 1 nM DA under identical experimental conditions. The dashed region in panel (a) is enlarged in the inset to highlight the negligible change in the MB oxidation peak following DA addition. In contrast, the functional DA-specific aptamer (panel b) exhibited a pronounced increase in the MB oxidation peak after exposure to 1 nM DA. Measurements were obtained from three independently fabricated neural probes, each containing two independent electrochemical sensing units (total *N* = 6 sensing units per group). For each sensing unit, SWV measurements were repeated three times. Solid/long dash lines represent the mean SWV responses, and the shaded regions represent the SDs.

Table S5. Quantitative analysis of the SWV peak currents extracted from **Figure S13a** for the scrambled aptamer control. Within-probe reproducibility was evaluated using the two independent electrochemical sensing units integrated within each neural probe (Neural Probes #S19-#S21), whereas probe-to-probe reproducibility represents the pooled statistical analysis across three independently fabricated neural probes. Peak current (I_{peak}) values are presented as mean ± SD, and the corresponding CV% values quantify measurement variability at both the sensing-unit and neural-probe levels. Measurements were obtained from three independently fabricated neural probes, each containing two independent sensing units (total *N* = 6 sensing units), with three replicate SWV measurements performed for each sensing unit under each experimental condition.

Solution	**Scrambled aptamer**							
	**Within-probe**						**Inter-probe**	
	**Neural probe #S19**		**Neural probe #S20**		**Neural probe #S21**			
	**I_{peak} (nA)**	**CV%**	**I_{peak} (nA)**	**CV%**	**I_{peak} (nA)**	**CV%**	**I_{peak} (nA)**	***CV%***
**aCSF**	7.8599 ± 0.1559	1.98	7.7956 ± 0.1077	1.38	7.5052 ± 0.1147	1.53	7.7225 ± 0.2113	2.74
**1 nM DA**	7.9673 ± 0.1460	1.83	7.9585 ± 0.0477	0.60	7.6002 ± 0.1156	1.52	7.8449 ± 0.2256	2.88

Note: The scrambled aptamer sequence used as a negative control was 5'-HO-C6-S-S-C6-GGT CAT GCT GTA CGT ACG A-MB-GGC TTG TGC GGT AGC TGG TAC GGC A-3'. The sequence contained the same nucleotide composition, thiol linker, and MB redox reporter as the DA aptamer but with a randomized nucleotide arrangement to disrupt DA-specific binding.

In contrast, the functional DA aptamer generated a pronounced electrochemical response under the identical experimental conditions (**Figure S13b**). As summarized in **Table S6**, the average peak current increased from 7.7618 ± 0.2815 nA in DA-free aCSF to 17.4000 ± 0.2103 nA following exposure to 1 nM DA, corresponding to a mean current increase of 9.6382 nA, which substantially exceeded the baseline variation. Because the functional DA aptamer sensor and the scrambled aptamer control differed only in the integrity of the DA-recognition sequence while sharing the same electrode architecture, AuNS modification, surface immobilization chemistry, MB labeling strategy, and electrochemical interrogation conditions, the markedly different electrochemical responses can be attributed to sequence-specific aptamer-DA recognition rather than nonspecific DA adsorption.

Table S6. Quantitative analysis of the SWV peak currents extracted from **Figure S13b** for the functional DA-specific aptamer. Within-probe reproducibility was evaluated using the two independent electrochemical sensing units integrated within each neural probe (Neural Probes #S10-#S12), whereas probe-to-probe reproducibility represents the pooled statistical analysis across three independently fabricated neural probes. Peak current (I_{peak}) values are presented as mean ± SD, and the corresponding CV% values quantify measurement variability at both the sensing-unit and neural-probe levels. Measurements were obtained from three independently fabricated neural probes, each containing two independent sensing units (total N = 6 sensing units), with three replicate SWV measurements performed for each sensing unit.

Solution	**DA-specific aptamer**							
	**Within-probe**						**Inter-probe**	
	**Neural probe #S10**		**Neural probe #S11**		**Neural probe #S12**			
	**I_{peak} (nA)**	**CV%**	**I_{peak} (nA)**	**CV%**	**I_{peak} (nA)**	**CV%**	**I_{peak} (nA)**	***CV%***
**aCSF**	7.9150 ± 0.1646	2.08	7.4552 ± 0.1768	2.37	7.7851 ± 0.1061	1.36	7.7618 ± 0.2815	3.63
**1 nM DA**	17.1751 ± 0.0791	0.46	17.5125 ± 0.1451	0.83	17.4562 ± 0.1123	0.64	17.4000 ± 0.2103	1.21

These new experimental results also provide additional evidence supporting our previous interpretation regarding polydopamine formation. Under the present experimental conditions, including near-physiological pH, nanomolar DA concentration, and transient SWV interrogation, any progressive DA-derived surface fouling would be expected to affect both the functional and scrambled probes similarly because such processes are governed primarily by the electrochemical environment rather than by the DNA sequence. The absence of measurable signal enhancement in the scrambled aptamer therefore further supports the conclusion that progressive polydopamine deposition[9] is unlikely to be the dominant mechanism responsible for the observed electrochemical response.

Note 7. XPS characterization of aptamer immobilization on AuNS-Modified WEs of the neural probe

XPS was employed to analyze the surface elemental composition and chemical bonding states of AuNS-modified WEs before and after aptamer immobilization. XPS measurements were performed using an XPS system equipped with a monochromatic Al Kα X-ray source (photon energy = 1486.6 eV). The base pressure in the analysis chamber was maintained below 1×10^{-9} mbar during data acquisition.

Prior to analysis, samples were gently rinsed with deionized water to remove loosely bound species and dried under a nitrogen stream. For each condition (AuNS-modified WE and aptamer-functionalized WE), spectra were collected from multiple locations on the electrode surface to ensure reproducibility. Peak fitting and deconvolution were carried out using a Gaussian-Lorentzian mixed line shape after Shirley background subtraction.

Survey spectra (**Figure S14a, b**) were used to compare relative elemental compositions before and after aptamer immobilization. A decrease in the relative Au atomic percentage after aptamer modification was consistently observed, consistent with the formation of an organic molecular overlayer covering the gold surface.

High-resolution $Au\ 4f$ spectra acquired before (**Figure S14b**) and after (**Figure S14c**) aptamer immobilization were further analyzed to identify changes in gold chemical states. Deconvolution of the $Au\ 4f$ region (**Figure S14d**) revealed the appearance of an additional component centered at ~87.4 eV following aptamer functionalization, which is characteristic of Au-S bonding. This feature indicates covalent attachment of thiolated aptamers to the AuNS-modified WEs of the neural probe through sulfur-gold interactions.

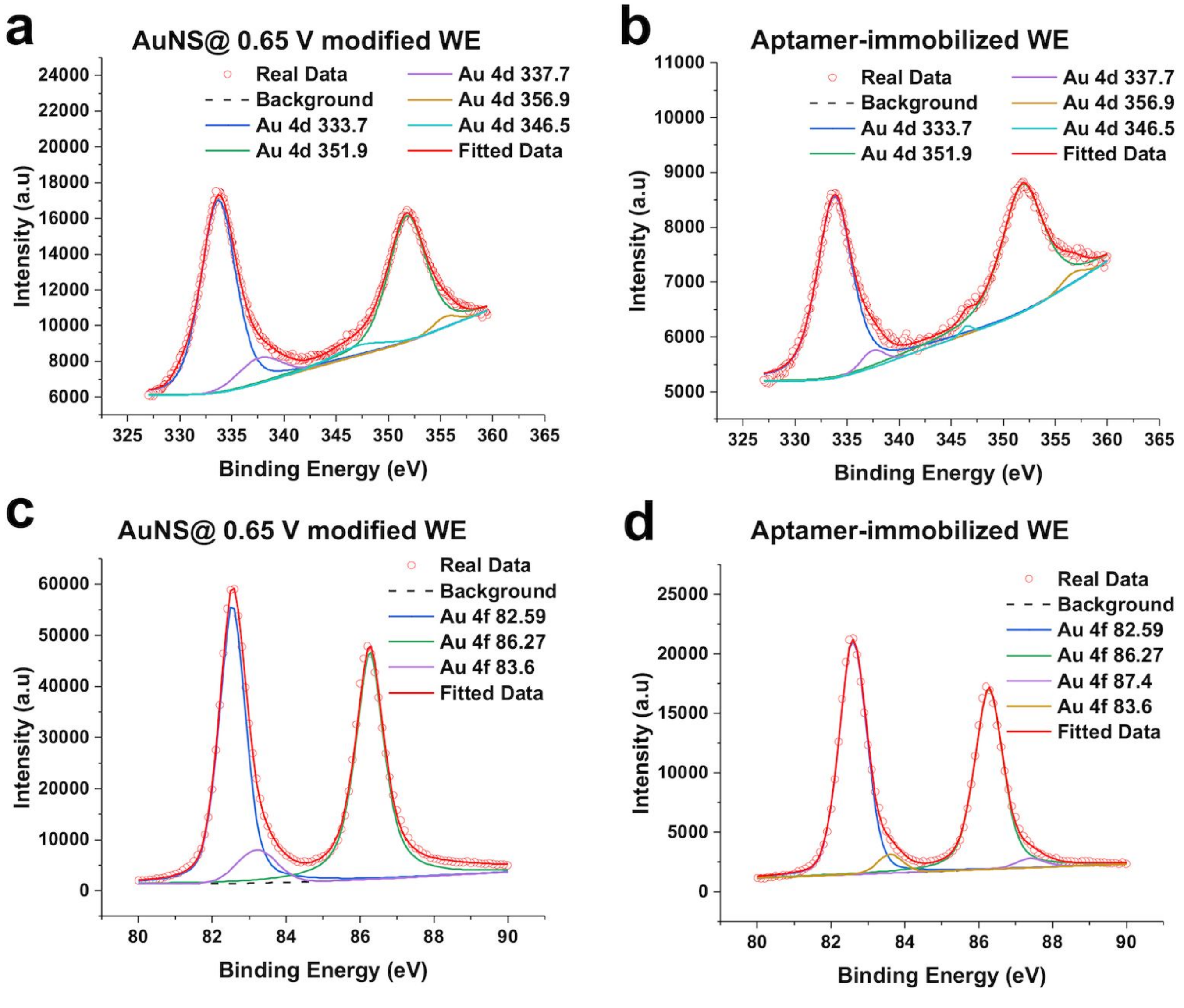

Figure S14. XPS characterization of AuNS-modified WEs of the neural probe before and after aptamer immobilization. (a) Survey spectra showing changes in elemental composition; a decrease in Au signal intensity after aptamer attachment is consistent with formation of an organic molecular overlayer. (b) $Au\ 4f$ core-level spectra of the AuNS-modified WE prior to functionalization. (c) $Au\ 4f$ core-level spectra after aptamer immobilization. (d) Deconvoluted $Au\ 4f$ spectra highlighting an additional component at ~87.4 eV

attributable to Au-S bonding, indicative of successful covalent attachment of thiolated aptamers to the gold surface *via* S-Au interactions. Spectra were normalized to facilitate comparison of peak features.

To further corroborate aptamer immobilization, high-resolution $C\ 1s$ spectra were examined. As shown in **Figure S15a**, the AuNS-modified WE prior to functionalization exhibited a relatively low carbon signal. After aptamer immobilization, an increased contribution from C-O bonding at ~285.34 eV was observed in the $C\ 1s$ spectrum (**Figure S15b**), consistent with the presence of nucleic-acid backbones and associated oxygen-containing functional groups on the electrode surface.

Therefore, the emergence of Au-S bonding signatures in the $Au\ 4f$ spectra (**Figure S14**) and the increased C-O contribution in the $C\ 1s$ spectra (**Figure S15**) provide complementary and direct evidence that thiolated DA-binding aptamers were successfully immobilized onto the AuNS-modified working electrodes via covalent sulfur-gold linkages.

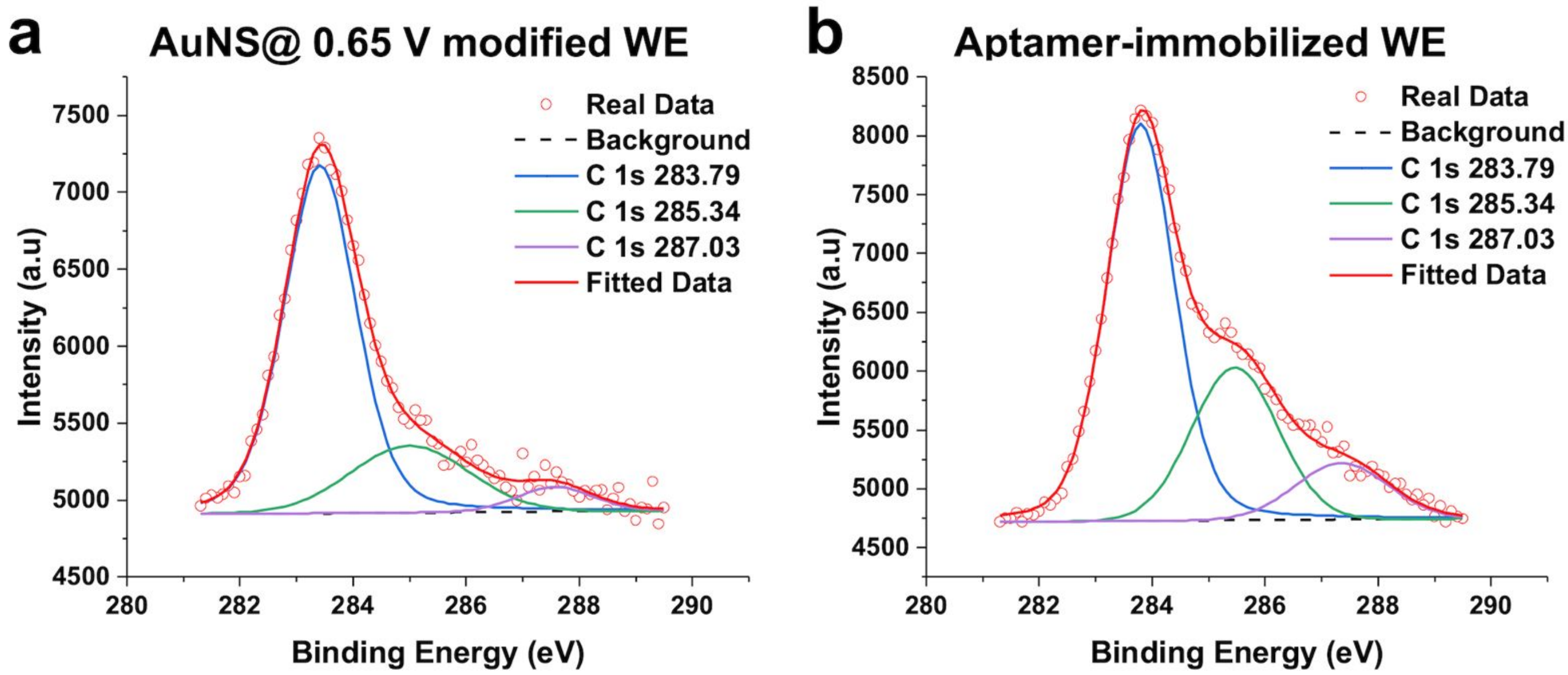

Figure S15. High-resolution $C\ 1s$ XPS spectra of AuNS-modified WEs of the neural probe before and after aptamer functionalization. (a) $C\ 1s$ spectrum of the AuNS-modified WE prior to aptamer immobilization, showing baseline carbon signal. (b) $C\ 1s$ spectrum after aptamer modification, exhibiting an increased contribution from C-O bonding near ~285.34 eV, consistent with nucleic acid backbone and associated functional groups introduced by the DA-binding aptamer. The enhanced C-O component, together with Au-S features in **Figure S14**, supports successful immobilization of thiolated aptamers on the electrode surface.

Note 8. Within-probe reproducibility of the equivalent-circuit fitting parameters

Table S7. Within-probe reproducibility of the extracted EIS fitting parameters (R_{ct} Q, and n) at different stages of surface modification based on the equivalent circuit model ($R_S - (CPE \parallel (R_{ct} + Z_w))$). The table summarizes the fitted EIS parameters for the bare AuNS surface, the aptamer-functionalized AuNS surface, and the AuNS surface after subsequent 6-MCH backfilling. Data are reported as mean ± SD together with the corresponding within-probe CV% for each independently fabricated neural probe (Probe #1-#3), each comprising two independent electrochemical sensing units. The within-probe CV% values demonstrate consistent electrochemical interface characteristics with low within-probe variability following aptamer immobilization and 6-MCH backfilling, further supporting the reproducibility of the interfacial modification process.

Surface modification	Probe #1		Probe #2		Probe #3	
	mean ± SD	CV %	mean ± SD	CV %	mean ± SD	CV %
Bare AuNS R_{ct} (Ω)	3055 ± 100	3.27	3355 ± 283	8.43	3405 ± 70.71	2.08
Aptamer R_{ct} (Ω)	8023 ± 370	4.61	8181 ± 146	1.78	8515 ± 117	1.37
Aptamer + 6-MCH R_{ct} (Ω)	4216 ± 148	3.52	4338 ± 37	0.86	4851 ± 134	2.77
Bare AuNS Q ($S \cdot s^n$)	(4.7 ± 0.09) ×10^{-7}	1.96	(4.8 ± 0.1) ×10^{-7}	2.05	(4.8 ± 0.03) ×10^{-7}	0.71
Aptamer Q ($S \cdot s^n$)	(3.0 ± 0.06) ×10^{-7}	1.86	(3.1 ± 0.05) ×10^{-7}	1.59	(3.1 ± 0.07) ×10^{-7}	2.30
Aptamer + 6-MCH Q ($S \cdot s^n$)	(2.2 ± 0.1) ×10^{-7}	5.07	(2.4 ± 0.09) ×10^{-7}	3.87	(2.5 ± 0.03) ×10^{-7}	1.09
Bare AuNS n	0.88 ± 0.01	1.29	0.86 ± 0.02	2.31	0.89 ± 0.04	4.69
Aptamer n	0.80 ± 0.01	1.59	0.82 ± 0.01	0.95	0.83 ±0.02	2.73
Aptamer + 6-MCH n	0.85 ± 0.01	0.92	0.85 ± 0.01	1.24	0.83 ± 0.01	1.36

Note 9. Calibration characteristics, detection performance, and absolute linear response analysis

We performed additional measurements at femtomolar DA concentrations (5 fM) and included replicate SWV analyses near the experimentally determined detection limit. It includes mean SWV voltammograms obtained from three independently fabricated neural probes, each containing two separate on-chip three-electrode electrochemical systems with independent WEs (total $N = 6$ sensing units [WEs]). For each sensing unit, measurements at each DA concentration were repeated 10 times. The results for DA-free aCSF (blank), 5 fM, 10 fM, and 100 fM DA solutions obtained using the integrated on-chip three-electrode system are presented in **Figure S16**. Corresponding measurements acquired using a conventional three-electrode system with standalone commercial Ag/AgCl RE and platinum CE are shown in **Figure S17**. In both figures, corresponding Panel (a) presents the averaged SWV voltammograms, while Panel (b) summarizes the corresponding maximum peak currents extracted from the SWV responses. These additional datasets were specifically included to directly evaluate whether meaningful signals can be resolved above the blank distribution near the proposed LOD region.

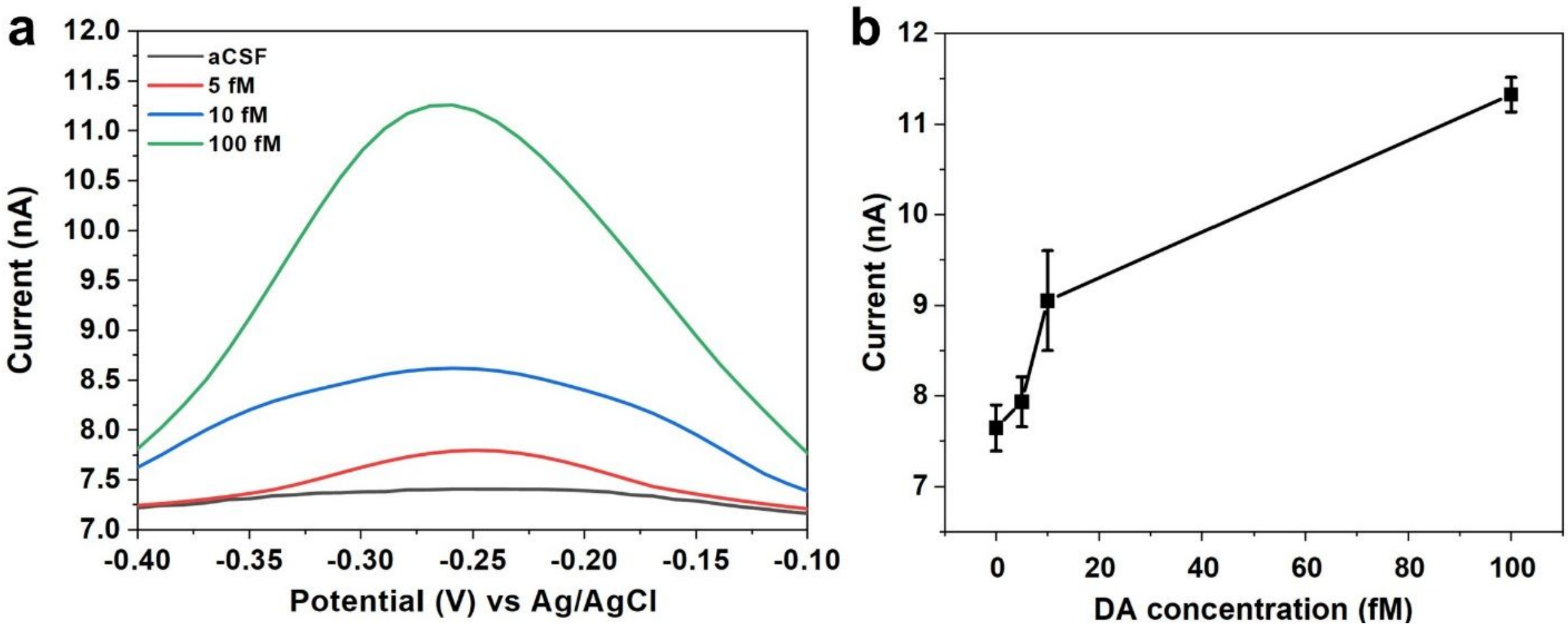

Figure S16. **Statistical validation of femtomolar DA detection using the integrated on-chip three-electrode electrochemical system**. (a) Mean SWV voltammograms obtained in DA-free aCSF (blank), 5 fM, 10 fM, and 100 fM DA solutions using the integrated on-chip three-electrode system. The 10 fM DA condition exhibits a clearly distinguishable increase in peak current relative to the blank signal, whereas the 5 fM response remains close to the background distribution. (b) Corresponding maximum SWV peak currents extracted from panel (a). Measurements were obtained from three independently fabricated neural probes, each containing two independent on-chip three-electrode electrochemical systems (total $N = 6$ sensing units). For each sensing unit, electrochemical measurements at each DA concentration were repeated 10 times. Error bars represent SD. The response approaches saturation at elevated DA concentrations, consistent with a finite binding-mediated sensing process. The response at 10 fM exceeds the $3\sigma_{blank}$ threshold, supporting 10 fM as the experimentally validated LOD of the integrated on-chip sensor.

The blank signal distribution was determined from repeated DA-free aCSF measurements ($N = 6$), yielding a SD of σ_{blank} = 0.2460 nA for the integrated on-chip three-electrode system (**Table S8**). Based on the conventional signal-to-noise criterion, the detection threshold was defined as $3\sigma_{blank}$, corresponding to 0.738 nA above the blank signal. Under these conditions, the measured response at 5 fM DA was $\Delta I = 0.2881 \pm 0.1795$ nA, which does not exceed the $3\sigma_{blank}$ threshold and therefore cannot be considered a reliably detectable signal. In contrast, the response obtained at 10 fM DA was $\Delta I = 1.4056 \pm 0.5521$ nA, which clearly exceeds the threshold and is distinguishable from the blank distribution. Accordingly, the experimentally validated LOD of the integrated on-chip sensor was 10 fM in this study.

As shown in **Figure S16a**, the 5 fM SWV response remains highly overlapped with the blank signal and exhibits only minimal signal separation, whereas the 10 fM DA condition produces a visibly distinguishable increase in peak current relative to the blank. The corresponding peak current analysis in **Figure S16b** further demonstrates that the 10 fM response becomes clearly separated from the blank variation, while the 5 fM response remains within the noise distribution range.

For comparison, the same measurements were performed using a conventional three-electrode configuration with standalone commercial Ag/AgCl RE and platinum CE (**Figure S17**). Under identical DA

concentrations, the conventional system exhibited substantially smaller signal changes and significantly larger variability at femtomolar DA concentrations. In particular, the 5 fM and 10 fM responses remained largely indistinguishable from the blank signal distribution, as reflected by the large error bars and high CV% values in summarized in **Table S8**. These results further highlight the improved signal-to-noise characteristics and lower background variability achieved by the integrated on-chip three-electrode configuration.

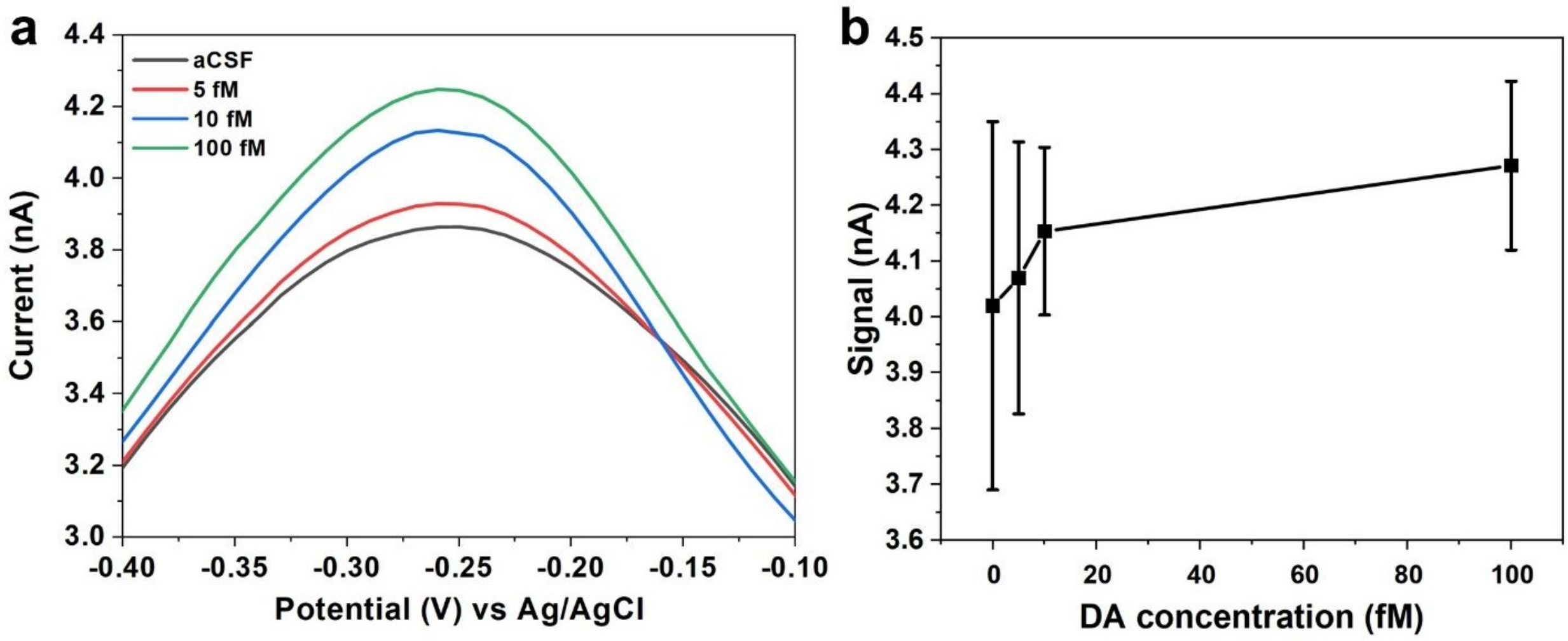

Figure S17. Femtomolar DA detection using a conventional three-electrode electrochemical system. (a) Mean SWV voltammograms measured in DA-free aCSF (blank), 5 fM, 10 fM, and 100 fM DA solutions using a conventional three-electrode configuration with standalone commercial Ag/AgCl RE and platinum CE. (b) Corresponding maximum SWV peak currents extracted from panel (a). Measurements were obtained from three independently fabricated neural probes, each containing two independent WEs that were measured using the external commercial Ag/AgCl RE and platinum CE configuration (total $N = 6$ sensing units). For each sensing unit, electrochemical measurements at each DA concentration were repeated 10 times. Error bars represent SD. The response approaches saturation at elevated DA concentrations, consistent with a finite binding-mediated sensing process. The 5 fM and 10 fM signals remain close to the blank distribution, indicating limited signal distinguishability near the experimentally determined detection limit.

Table S8. Probe-to-probe variability. Corresponding statistical analysis of calibration data, including mean, SD, and CV%, for the integrated on-chip three-electrode system (Neural Probe #4-#6) and conventional three-electrode systems (Neural Probe #7-#9) across the full DA concentration range.

Concentration (M)	*Integrated on-chip three-electrode system*			*Conventional three-electrode systems*		
	ΔI Mean (nA)	*SD (nA)*	*CV%*	*ΔI Mean (nA)*	*SD (nA)*	*CV%*
**Blank**	I = 7.6450	0.2460	-	I = 4.0220	0.3300	-
**5×10^{-15}**	0.2881	0.1795	62.31	0.0501	0.2438	486.63
**10^{-14}**	1.4056	0.5521	39.28	0.1336	0.1503	112.50
**10^{-13}**	3.6802	0.1907	5.18	0.2512	0.1512	60.19
**10^{-12}**	5.0937	0.3352	6.58	1.0408	0.2301	22.10
**10^{-11}**	7.1948	0.1162	1.62	1.4977	0.2249	15.01
**10^{-10}**	8.7989	0.2059	2.34	2.3001	0.2417	10.51
**10^{-9}**	10.1659	0.3913	3.85	2.5825	0.2566	9.94
**10^{-8}**	11.2700	0.0457	0.41	2.7081	0.2881	10.64
**10^{-7}**	11.9454	0.2775	2.32	2.8363	0.1966	6.93
**10^{-6}**	13.1579	0.1267	0.96	3.2500	0.1812	5.57
**2.5×10^{-6}**	13.7152	0.1649	1.20	3.2609	0.2042	6.26
**5×10^{-6}**	13.7423	0.1812	1.32	3.2711	0.2744	8.39
**10^{-5}**	13.7808	0.1775	1.29	3.2696	0.2287	7.00

Note: The blank value represents the mean absolute peak current (I), while the values at all other concentrations are expressed as the change in peak current (**Δ*I***) relative to the blank. Coefficient of variation (CV%) at each concentration was calculated as CV% = SD/mean × 100 %. Measurements were obtained from three independently fabricated neural probes, each containing

two independent on-chip three-electrode electrochemical systems (total N = 6 sensing units). For each sensing unit, electrochemical measurements at each concentration were repeated 10 times.

To determine the measurement saturation at higher DA concentrations, we performed additional measurements at elevated DA concentrations, including 2.5 μM, 5 μM, and 10 μM DA, and the corresponding results are now provided in **Figure S18** for the integrated on-chip three-electrode system and **Figure S19** for the conventional three-electrode system using standalone commercial Ag/AgCl RE and platinum CE.

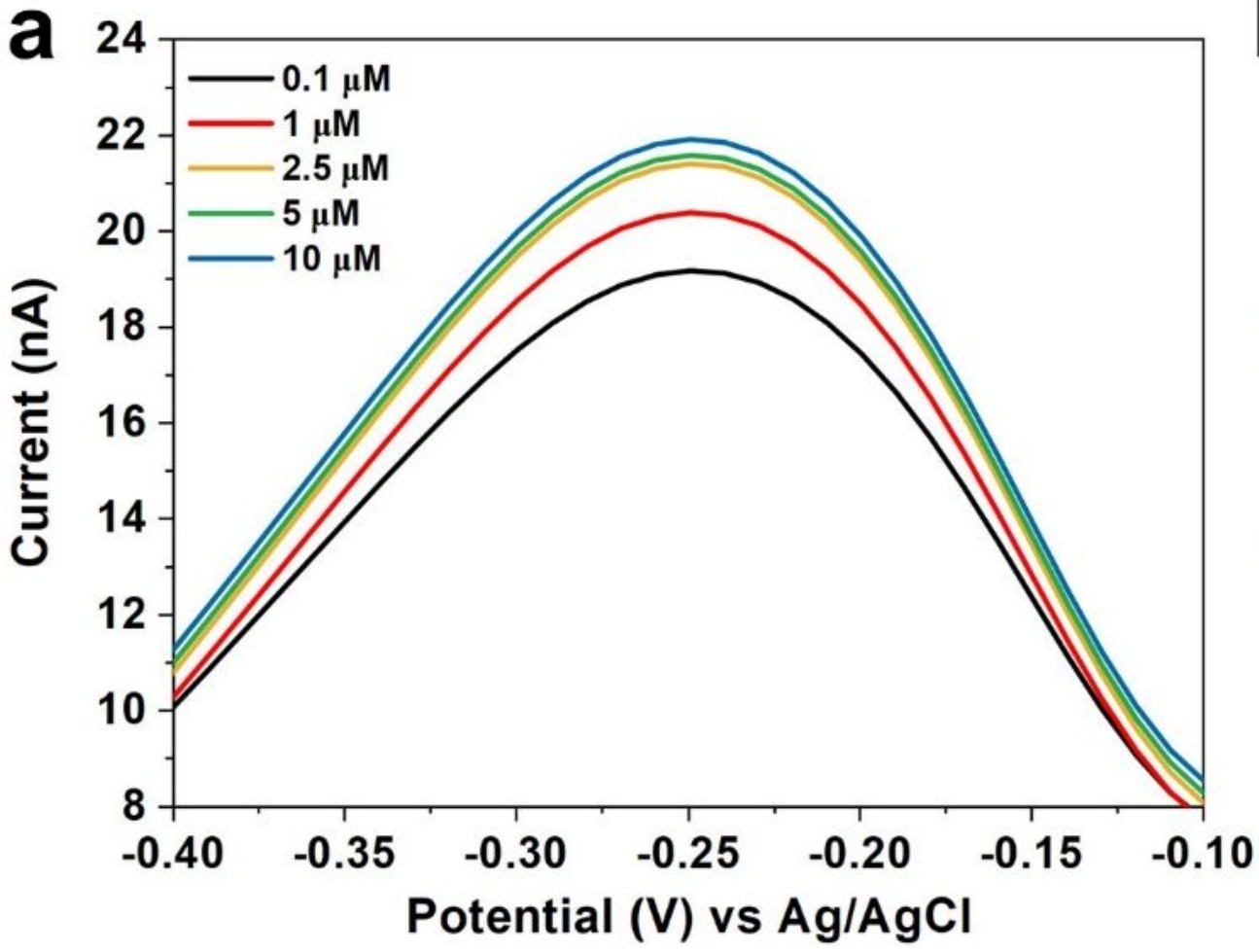

Figure S18. **SWV responses of the integrated on-chip three-electrode electrochemical system at high DA concentrations**. (a) Mean SWV voltammograms obtained in DA solutions of 0.1 μM, 1 μM, 2.5 μM, 5 μM, and 10 μM. (b) Corresponding maximum SWV peak currents extracted from panel (a). Measurements were obtained from three independently fabricated neural probes, each containing two independent on-chip three-electrode electrochemical systems (total N = 6 sensing units). For each sensing unit, electrochemical measurements at each DA concentration were repeated 10 times. Error bars represent SD. The response approaches saturation at elevated DA concentrations, consistent with a finite binding-mediated sensing process.

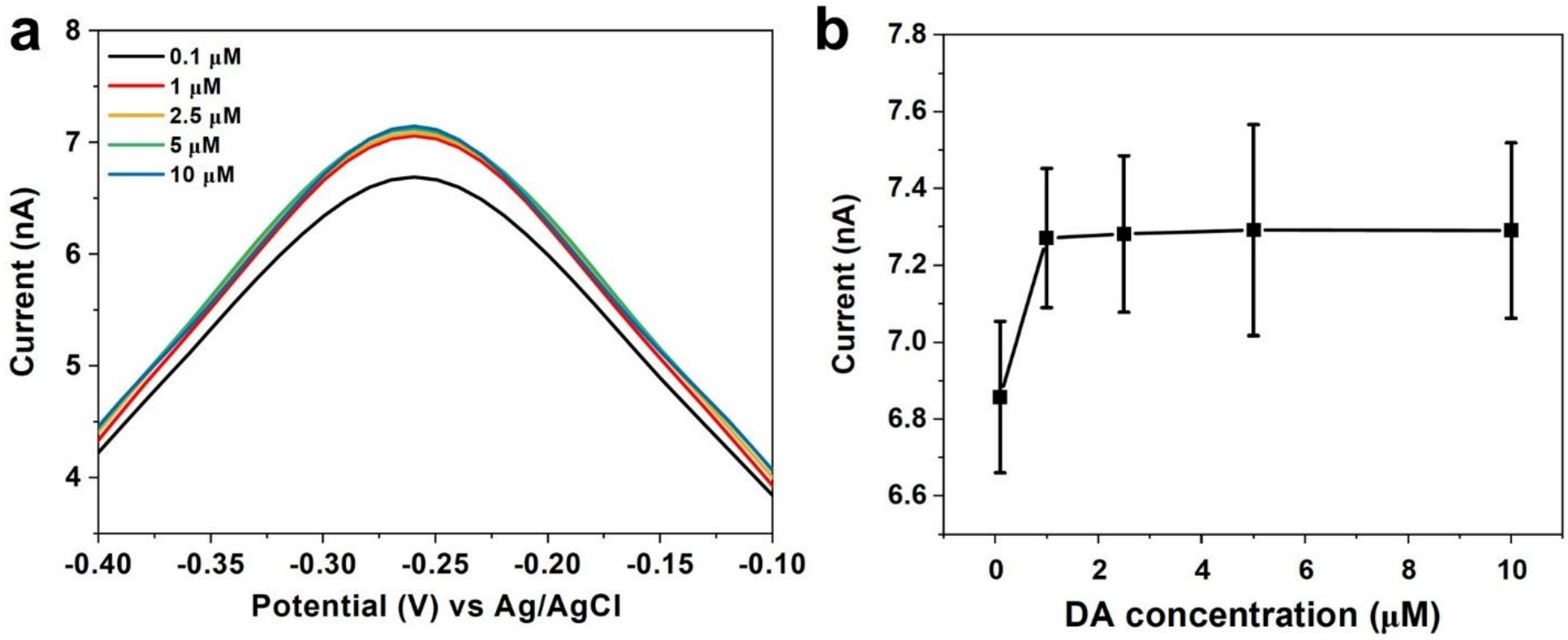

Figure S19. SWV responses of the conventional three-electrode electrochemical system at high DA concentrations. (a) Mean SWV voltammograms obtained in DA solutions of 0.1 μM, 1 μM, 2.5 μM, 5 μM, and 10 μM. (b) Corresponding maximum SWV peak currents extracted from panel (a). Measurements were obtained from three independently fabricated neural probes, each containing two independent WEs (total N = 6 sensing units). For each sensing unit, electrochemical measurements at each DA concentration were repeated 10 times. Error bars represent SD. The response shows saturation-like behavior at elevated DA concentrations.

The data demonstrate that our sensor response does not continue to increase linearly across the entire concentration range. As shown in **Figure S18a, b**, the peak SWV current increases substantially from 0.1 μM to 1 μM dopamine, but the incremental signal enhancement becomes progressively smaller between 2.5 μM, 5 μM, and 10 μM. In particular, the signal at 10 μM exhibits only a marginal increase relative to 5 μM, indicating the onset of response saturation. This saturation behavior is characteristic of a binding-limited

process and is consistent with specific aptamer-DA interactions. In contrast, nonspecific adsorption would typically result in a continuously increasing signal without a defined plateau. Therefore, the observed saturation at higher concentrations provides direct evidence that the sensing mechanism is governed by specific binding rather than nonspecific surface interactions[10].

The absence of saturation within the reported calibration range (10 fM-1 μM) is therefore not indicative of nonspecific binding, but rather reflects the selected working concentration window. This range was intentionally chosen to cover physiologically relevant DA concentrations in the extracellular brain environment, which are typically within the nanomolar (nM) to submicromolar (sub-μM) regime[11]. Within this biologically relevant range, the sensor operates in a dynamic pre-saturation region, enabling sensitive quantification of DA concentration changes without substantial signal compression. The inclusion of the newly added higher-concentration measurements (**Figures S18** and **S19**) further confirms that the sensing response transitions into a saturation regime outside this working range, consistent with expected finite binding behavior.

To further characterize the quantitative analytical performance of the integrated on-chip three-electrode sensing system, direct linear regression was performed using the non-transformed DA concentration data over the experimentally investigated concentration range (**Figure S20**). Unlike the semi-logarithmic calibration presented in the main manuscript (**Figure 3c**), which describes the broader detectable concentration range of the sensing platform, this analysis specifically aimed to identify concentration intervals exhibiting direct linearity with absolute DA concentration.

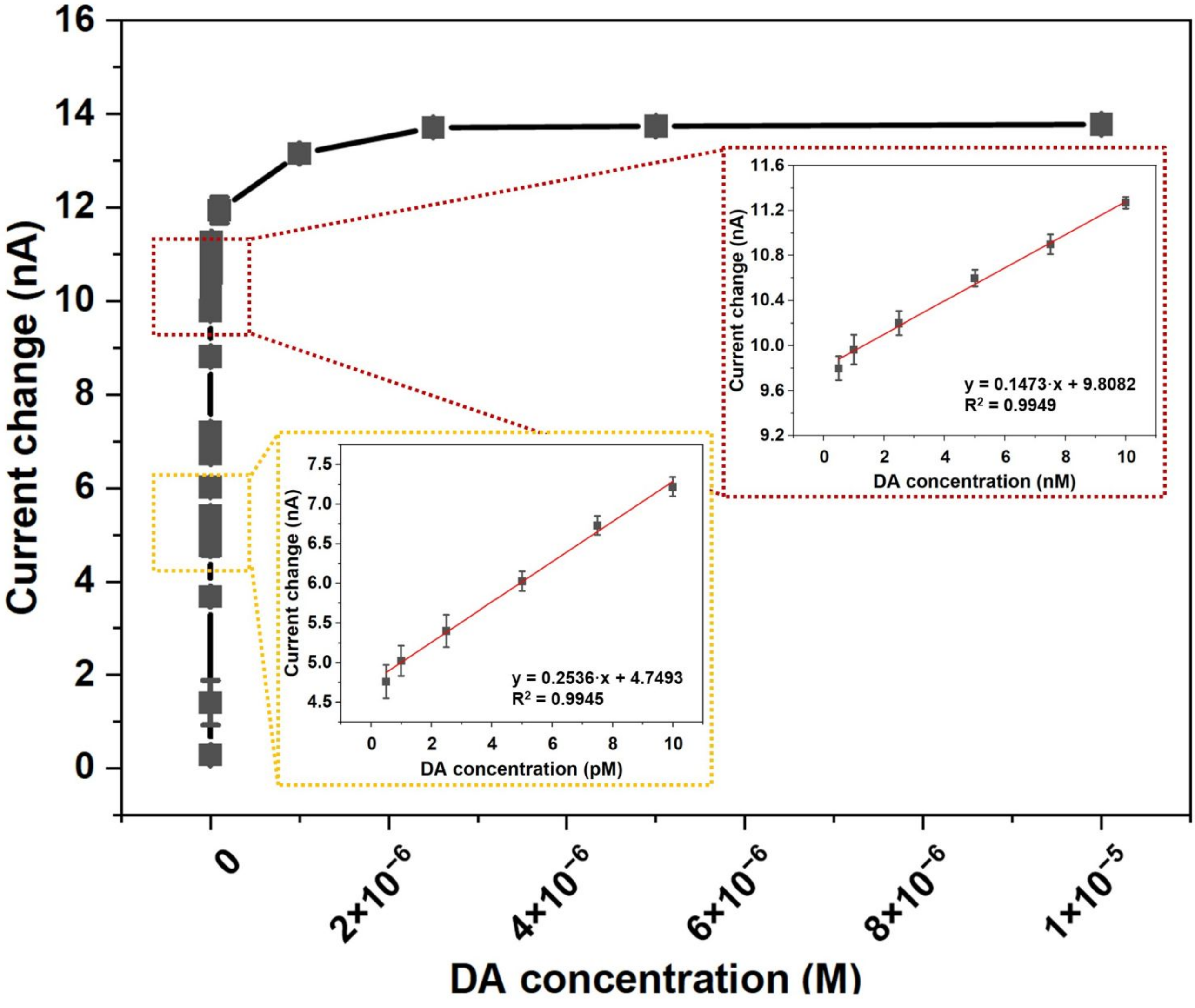

Figure S20. **Identification of the absolute linear response regions within the broader detectable concentration range of the integrated on-chip three-electrode sensing system.** The main plot illustrates the concentration-dependent electrochemical response of the integrated on-chip three-electrode system over the experimentally investigated DA concentration range. Direct linear regression of the non-transformed concentration data identified two absolute linear response regions at 0.5-10 pM (yellow inset; R^2 = 0.9945) and 0.5-10 nM (red inset; R^2 = 0.9949). The intermediate concentration interval between these two regions

remained well described by the overall semi-logarithmic calibration relationship but did not satisfy the statistical criteria required for direct linear regression. Accordingly, the broader detectable concentration range and the absolute linear response regions represent distinct analytical performance characteristics of the sensing platform.

The regression analysis identified two discrete absolute linear response regions. The first extended from 0.5 to 10 pM (R^2 = 0.9945), representing the trace-level quantitative analytical region. The second extended from 0.5 to 10 nM (R^2 = 0.9949), corresponding to the physiologically relevant nanomolar concentration regime. Both regions exhibited excellent linearity and therefore define the quantitative operating ranges of the integrated sensing platform.

Between 10 pM and 0.5 nM, the electrochemical response remained monotonic but no longer satisfied the statistical criteria required for direct linear regression using absolute concentration. Nevertheless, this intermediate concentration interval remained well described by the overall semi-logarithmic calibration relationship shown in **Figure 3c**. Such behavior is consistent with the equilibrium-controlled conformational switching mechanism characteristic of electrochemical aptamer-based sensors, in which target binding progressively shifts the equilibrium population of surface-confined aptamer conformations, producing a continuous nonlinear calibration response rather than a single absolute linear relationship across the entire detectable concentration range [12]. Consequently, this concentration interval should be interpreted as a nonlinear transition region connecting two quantitative linear response regions rather than as an additional linear analytical range.

Note 10. Probe-to-probe reproducibility and within-probe reproducibility

To evaluate the reproducibility across independently fabricated neural probes (probe-to-probe reproducibility), we have additionally provided the complete calibration statistics in ***Supplementary Note 9*** (**Table S8**), including the mean signal change (ΔI), SD, and CV% across the full DA concentration range for both the integrated on-chip three-electrode system and the conventional three-electrode system with an external saturated calomel electrode (SCE). The blank values represent the mean absolute SWV current measured in dopamine-free artificial cerebrospinal fluid (aCSF), whereas all other values are expressed as changes in current (ΔI) relative to the corresponding blank signal. Measurements were obtained from three independently fabricated neural probes, each integrating two independent electrochemical sensing units, yielding a total of six independent sensing units ($N = 6$). For each sensing unit, electrochemical measurements at every DA concentration were repeated ten times. The statistical values reported in **Table S8** therefore summarize the overall analytical reproducibility across independently fabricated neural probes (probe-to-probe reproducibility). To further evaluate the reproducibility between the two sensing units integrated within each neural probe (within-probe reproducibility), the individual calibration results of each sensing unit are additionally provided in **Tables S9** and **S10**.

As shown in **Table S9**, some variability between individual sensing units becomes more noticeable at the lowest DA concentrations, particularly near the experimentally determined LOD. Specifically, the integrated on-chip three-electrode configuration exhibited relatively high CV% at 5 fM (61.45%) and 10 fM (26.54%). However, these concentrations correspond to the LOD or are immediately adjacent to it, where the measured signal approaches the noise floor of the electrochemical system. Under such conditions, even small absolute peak current fluctuations can result in disproportionately large relative variations when expressed as CV%. Therefore, the elevated variability observed at these concentrations is expected for measurements performed near the analytical detection limit and should not be interpreted as evidence of instability in the sensing interface.

Importantly, as the DA concentration increased beyond the LOD and entered the quantitative working range (10^{-13}-10^{-12} M and above), the variability decreased substantially. For the integrated on-chip configuration, the CV% values rapidly declined to below approximately 5% across most of the calibration range, demonstrating good agreement between the two independently fabricated sensing units integrated within the same neural probe. Taken together, these results confirm that the observed variability is primarily associated with measurements performed near the detection limit, whereas the sensing platform exhibits good within-probe reproducibility throughout its practical quantitative operating range.

Table S9. Individual calibration results and within-probe variability of the integrated on-chip three-electrode system. Each independently fabricated neural probe (Neural Probe #4-#6) contains two independent electrochemical sensing units, yielding a total of six sensing units (3 probes × 2 sensing units). The table summarizes the peak current change (ΔI) and CV% for each sensing unit, enabling evaluation of within-probe reproducibility. The ΔI datasets are presented as mean ± SD.

Integrated on-chip three-electrode system						
Concn. (M)	**Neural probe #4**		**Neural probe #5**		**Neural probe #6**	
	**ΔI (nA)**	**CV%**	**ΔI (nA)**	**CV%**	**ΔI (nA)**	**CV%**
**Blank**	I =7.5304 ± 0.2687	-	I =7.5455 ± 0.0495	-	I =7.8611 ± 0.0424	-
**5×10^{-15}**	0.1087 ± 0.0668	61.45	0.3302 ± 0.1359	41.16	0.4159 ± 0.0502	12.07
**10^{-14}**	0.8650 ± 0.2296	26.54	1.5252 ± 0.3748	24.57	1.7009 ± 0.2478	14.57
**10^{-13}**	3.5031 ± 0.1131	3.23	3.7606 ± 0.0990	2.63	3.7828 ± 0.0588	1.55
**10^{-12}**	4.7615 ± 0.1768	3.71	5.1015 ± 0.2616	5.13	5.2352 ± 0.1697	3.24
**10^{-11}**	7.1224 ± 0.1202	1.69	7.1454 ± 0.0503	0.70	7.2277 ± 0.04649	0.64
**10^{-10}**	8.6458 ± 0.1909	2.21	8.8022 ± 0.1977	2.25	8.9048 ± 0.0856	0.96
**10^{-9}**	9.9657 ± 0.2101	2.11	10.1650 ± 0.0919	0.90	10.2731 ± 0.1482	1.44
**10^{-8}**	11.2561 ± 0.0728	0.65	11.2717 ± 0.0429	0.38	11.2750 ± 0.0071	0.06
**10^{-7}**	11.7343 ± 0.1584	1.35	11.9550 ± 0.0778	0.65	12.1752 ± 0.0354	0.29
**10^{-6}**	13.0800 ± 0.1418	1.08	13.1100 ± 0.0442	0.34	13.2199 ± 0.0318	0.24
**2.5×10^{-6}**	13.6040 ± 0.1819	1.34	13.6450 ± 0.0636	0.47	13.8148 ± 0.0482	0.35
**5×10^{-6}**	13.6256 ± 0.2192	1.61	13.7651 ± 0.1457	1.06	13.7894 ± 0.1340	0.97
**10^{-5}**	13.6742 ± 0.2051	1.50	13.7000 ± 0.0658	0.48	13.8610 ± 0.0707	0.51

A similar trend was observed for the conventional three-electrode configuration (**Table S10**). The within-probe variability increased markedly at DA concentrations below approximately 1 pM, which can be attributed to the considerably smaller absolute signal changes generated by this configuration at low concentrations. As

a result, the signal-to-noise ratio deteriorated more rapidly near the lower end of the calibration range, leading to larger relative variability. Nevertheless, despite the increased variation observed near the detection limit, both sensing units within each probe exhibited consistent concentration-dependent response behavior across the entire calibration range.

Table S10. Individual calibration results and within-probe variability of the conventional three-electrode system with an external SCE. Each independently fabricated neural probe (Neural Probe #7-#9) contains two independent electrochemical sensing units, yielding a total of six sensing units (3 probes × 2 sensing units). The table summarizes the peak current change (*ΔI*) and CV% for each sensing unit, enabling evaluation of within-probe reproducibility. The *ΔI* datasets are presented as mean ± SD.

Conventional three-electrode systems with SCE						
Concn. (M)	**Neural probe #7**		**Neural probe #8**		**Neural probe #9**	
	***ΔI* (nA)**	**CV%**	***ΔI* (nA)**	**CV%**	***ΔI* (nA)**	**CV%**
**Blank**	I =3.6809 ± 0.1447	-	I =4.0732 ± 0.2653	-	I =4.3119 ± 0.2171	-
**5×10^{-15}**	0.0589 ± 0.3485	591.62	0.0997 ± 0.3967	397.75	0.0595 ± 0.3493	587.08
**10^{-14}**	0.0894 ± 0.1643	183.89	0.1353 ± 0.1609	91.70	0.1790 ± 0.1744	97.41
**10^{-13}**	0.0949 ± 0.0663	69.86	0.3216 ± 0.1879	58.43	0.3372 ± 0.0332	9.85
**10^{-12}**	0.9338 ± 0.2859	30.62	0.9100 ± 0.0505	5.55	1.2787 ± 0.1009	7.89
**10^{-11}**	1.2653 ± 0.0986	7.79	1.6023 ± 0.2794	17.44	1.6256 ± 0.0493	3.03
**10^{-10}**	2.0503 ± 0.1060	5.17	2.3001 ± 0.1413	6.14	2.5500 ± 0.1060	4.16
**10^{-9}**	2.5832 ± 0.4876	18.88	2.5815 ± 0.2626	10.17	2.5827 ± 0.1500	5.81
**10^{-8}**	2.4103 ± 0.1268	5.26	2.7081 ± 0.1684	6.22	3.0059 ± 0.1253	4.17
**10^{-7}**	2.7449 ± 0.2443	8.90	2.7246 ± 0.0431	1.58	3.0395 ± 0.0863	2.84
**10^{-6}**	3.1657 ± 0.2251	7.11	3.1470 ± 0.0397	1.26	3.4373 ± 0.0795	2.31
**2.5×10^{-6}**	3.1659 ± 0.2537	8.01	3.3243 ± 0.2985	8.98	3.2926 ± 0.1641	4.98
**5×10^{-6}**	3.1435 ± 0.3410	10.85	3.1151 ± 0.0601	1.93	3.5547 ± 0.1203	3.38
**10^{-5}**	3.1332 ± 0.1003	3.20	3.3051 ± 0.1839	5.54	3.4706 ± 0.1504	4.33

Furthermore, because analytical reproducibility should also be maintained under realistic sensing conditions, we additionally evaluated the reproducibility of the selectivity experiments. The newly added **Table S11** summarizes both within-probe reproducibility (responses obtained from the two sensing units integrated within each neural probe) and inter-probe reproducibility (responses pooled across three independently fabricated neural probes) for DA and all tested interfering species. The results demonstrate excellent agreement between the two sensing units within each probe as well as highly consistent selectivity performance across independently fabricated neural probes, further confirming that the observed sensing characteristics are reproducible at both the sensing-unit level and the device level.

Table S11. **Within-probe and probe-to-probe reproducibility of the selectivity evaluation for the integrated on-chip three-electrode sensing platform**. Within-probe reproducibility was evaluated using the two independent sensing units integrated within each neural probe (Neural Probes #10-#12), whereas probe-to-probe reproducibility represents the pooled responses from three independently fabricated neural probes. Peak current change (*ΔI*) and CV% are reported for DA and all tested interfering species. The *ΔI* datasets are presented as mean ± SD.

Within-probe							Inter-probe	
Interferents	**Neural probe #10**		**Neural probe #11**		**Neural probe #12**		***ΔI* (nA)**	**CV%**
	***ΔI* (nA)**	**CV%**	***ΔI* (nA)**	**CV%**	***ΔI* (nA)**	**CV%**		
**DA**	12.8410 ± 0.0136	0.11	13.0612 ± 0.1003	0.77	12.8873 ± 0.2205	1.71	12.9298 ± 0.1502	1.16
**L-DOPA**	8.5093 ± 0.0480	0.56	8.4966 ± 0.0771	0.91	8.3979 ± 0.0757	0.90	8.5093 ± 0.0760	0.90
**L-Tyr**	8.1668 ± 0.0798	0.98	8.1521 ± 0.0408	0.50	8.0120 ± 0.0284	0.35	8.1097 ± 0.0880	1.09
**5-HT**	7.800 ± 0.0423	0.54	7.5906 ± 0.0290	0.38	7.8807 ± 0.0982	1.25	7.7571 ± 0.1428	1.84
**DOPAC**	7.9801 ± 0.0424	0.53	7.9352 ± 0.0779	0.98	7.7257 ± 0.2184	2.83	7.9087 ± 0.1751	2.21
**HVA**	7.7201 ± 0.0147	0.19	7.5701 ± 0.1697	2.24	7.8958 ± 0.1775	2.25	7.7287 ± 0.1826	2.36
**UA**	8.1806 ± 0.0417	0.51	8.3253 ± 0.0073	0.09	7.9604 ± 0.0706	0.89	8.1554 ± 0.1684	2.07
**3-MT**	7.6702 ± 0.0140	0.18	7.7662 ± 0.0352	0.45	7.5751 ± 0.0212	0.28	7.6705 ± 0.0876	1.14
**AA**	7.6455 ± 0.0219	0.29	7.7410 ± 0.0141	0.18	7.8606 ± 0.0701	0.89	7.7490 ± 0.1020	1.32
Note: Selectivity was evaluated using 1 pM DA. All interfering species were tested at 1 nM, except AA, which was tested at 1 µM.								

Note 11. Comparison of representative electrochemical aptamer-based DA sensors based on different electrode materials and surface modification strategies

Table S12. Performance comparison of reported electrochemical aptamer-based DA sensors.

Electrochemical Method	Electrode Surface Material	Sample Matrix	Absolute Linear Range	LOD	Ref.
**CV, SWV**	Graphene oxide/ Nile blue/ AuNPs	Serum	10 - 200 nM	1 nM	Hui Jin et *al*.[13]
**DPV, EIS**	Au nanocomposite-Prussian blue-CNT	Serum	0.5-50 nM	0.2 nM	Shabnam Beiranvand et *al*.[14]
**CV, DPV, EIS**	3D graphene-DNA composite	Serum	0.1-100 μM	30 nM	Liping Lu et *al*.[15]
**CV, DPV, EIS**	Au-Pt nanoparticles	Serum	1-30 nM	0.22 nM	Azadeh Azadbakht et *al*.[16]
**EIS**	Gold nanostar-modified electrode	Urine, Plasma	1-100 nM	1.9 pM	Rasoul Pourtaghavi Talemi et *al*.[17]
**EIS, DPV**	Graphene oxide-based aptasensor	Serum	1 pM-160 nM	78 fM	WentingWang et *al*.[18]
**DPV**	Spindle-shaped Au nanostructures	Serum	0.163-20 nM	10 pM	Ramezan Ali Taheri et *al*.[19]
**Amperometry**	Carbon fiber microelectrode	*in vivo* rat brain	0.05-20 μM	13 nM	Xin Li et *al*.[20]
**SWV**	AuNS-modified microelectrode array	*in vivo* rat brain	0.5 pM to 10 pM<br>0.5 nM to 10 nM	10 fM	This work

Notes: Linear range and LOD values are reported as stated in the corresponding references. All concentrations are expressed in molar units for consistency.

AuNPs = gold nanoparticles; CNT = carbon nanotube; CV = cyclic voltammetry; DPV = differential pulse voltammetry; SWV = square-wave voltammetry; EIS = electrochemical impedance spectroscopy; CSF = cerebrospinal fluid.

Note 12. Scan-to-scan reproducibility during dynamic DA sensing under in vitro and ex vivo conditions

To further distinguish the intrinsic electrochemical measurement reproducibility from the averaged SWV responses, scan-to-scan reproducibility was systematically evaluated under progressively more complex experimental conditions, including the FIA system and the *ex vivo* brain phantom model. For all dynamic experiments, one electrochemical sensing unit was continuously operated on each neural probe throughout the entire measurement period. Every electrochemical data point represents the arithmetic mean of three consecutive SWV scans, whereas the corresponding scan-to-scan CV% was calculated from these three individual SWV measurements to evaluate the intrinsic measurement reproducibility.

Dynamic FIA experiments were independently performed using three neural probes (Neural probes #13-#15), with one sensing unit operated on each neural probe throughout the experiment. For every electrochemical data point, the scan-to-scan CV% was calculated from the corresponding three consecutive SWV scans acquired at the same measurement time point.

As shown in **Figure S21**, transient increases in scan-to-scan CV% were consistently observed immediately following each DA injection into the FIA circulation system. Furthermore, the magnitude of this temporary increase became progressively larger as the injected DA concentration increased, reflecting the increasingly rapid concentration transition occurring during solution exchange. Importantly, however, the scan-to-scan CV% values remained below 10% throughout the entire experiment and rapidly returned to baseline once the DA concentration became stable. These observations indicate that the temporary increase in scan-to-scan variability originates primarily from transient concentration changes during solution exchange rather than intrinsic electrochemical measurement noise, demonstrating excellent scan-to-scan reproducibility of the proposed sensing platform during dynamic *in vitro* DA monitoring.

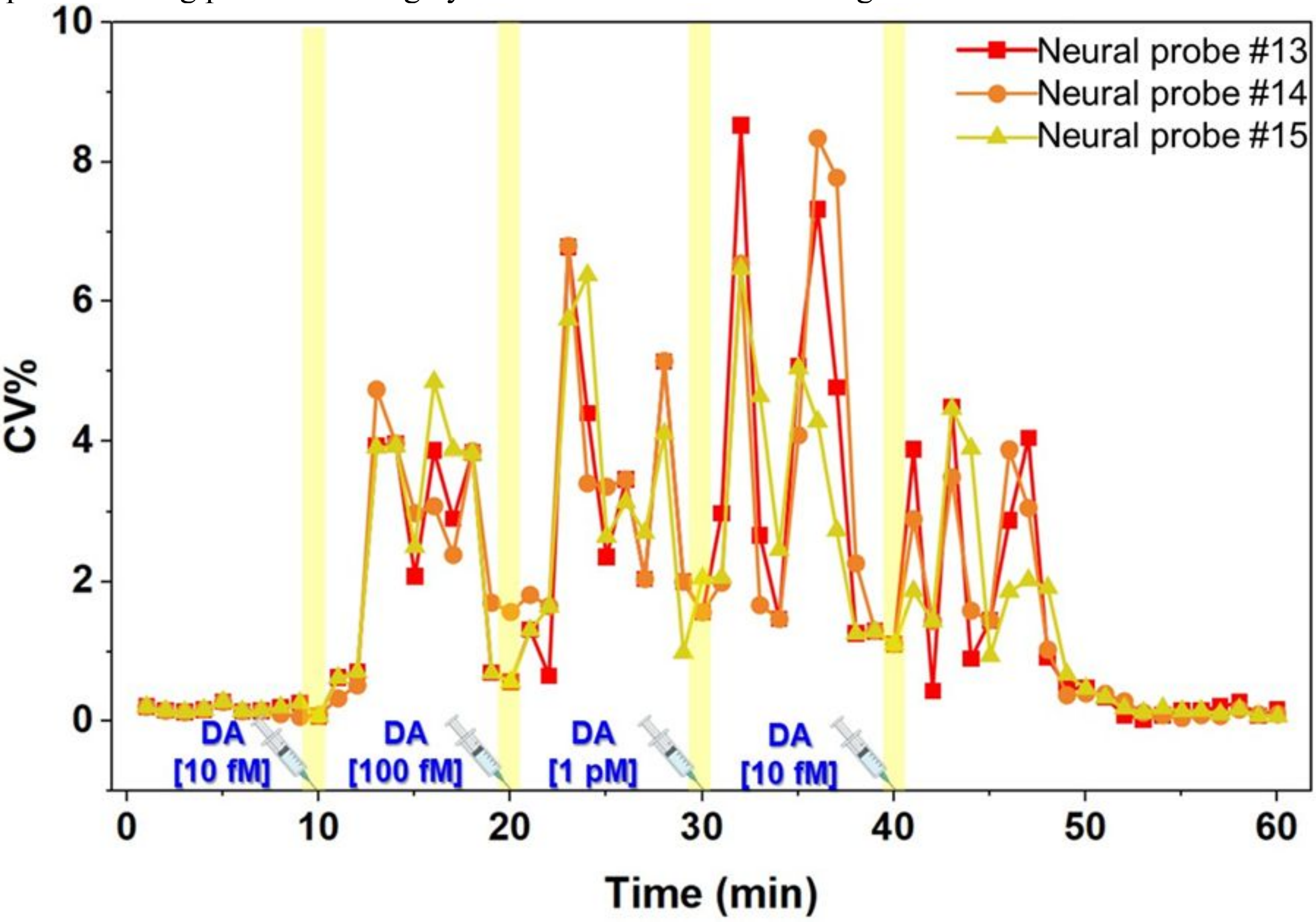

Figure S21. **Scan-to-scan reproducibility during dynamic DA monitoring in the FIA system**. The CV% for each electrochemical data point was calculated from three consecutive SWV scans acquired using a single sensing unit from three independently fabricated neural probes. Following sequential DA injections (10 fM, 100 fM, 1 pM, and 10 pM), the scan-to-scan CV% exhibited transient increases due to dynamic changes in DA concentration during solution exchange, and subsequently returned to baseline as the system approached equilibrium. Importantly, all sensing units maintained scan-to-scan CV% values below 10% throughout the experiment, demonstrating excellent measurement reproducibility during dynamic DA sensing.

To further verify that the excellent scan-to-scan reproducibility observed in the FIA system could be maintained under tissue-mimicking conditions, the same analysis was subsequently performed during dynamic DA monitoring in the *ex vivo* brain phantom model. Dynamic brain phantom experiments were

independently conducted using three additional neural probes (Neural probes #16-#18), again with one sensing unit operated on each neural probe throughout the experiment.

During repeated baseline aCSF injections, the scan-to-scan CV% remained consistently below 1%, demonstrating highly stable electrochemical measurements under steady-state conditions. Following sequential injections of 10 fM, 1 pM, and 100 pM dopamine, the scan-to-scan CV% exhibited a transient increase relative to the baseline because the local DA concentration surrounding the sensing interface continuously changed during the initial diffusion process. Consequently, small differences arose among the three consecutive SWV scans used to generate each averaged electrochemical data point. As DA gradually diffused throughout the brain phantom and the local concentration approached equilibrium, the scan-to-scan CV% progressively decreased and stabilized.

Importantly, all three neural probes maintained scan-to-scan CV% values below 10% throughout the entire experiment (**Figure S22**), confirming excellent scan-to-scan reproducibility under dynamic ex vivo neurochemical sensing conditions. These findings demonstrate that the temporary increase in scan-to-scan variability primarily reflects the transient diffusion kinetics of DA rather than instability of the electrochemical sensing platform.

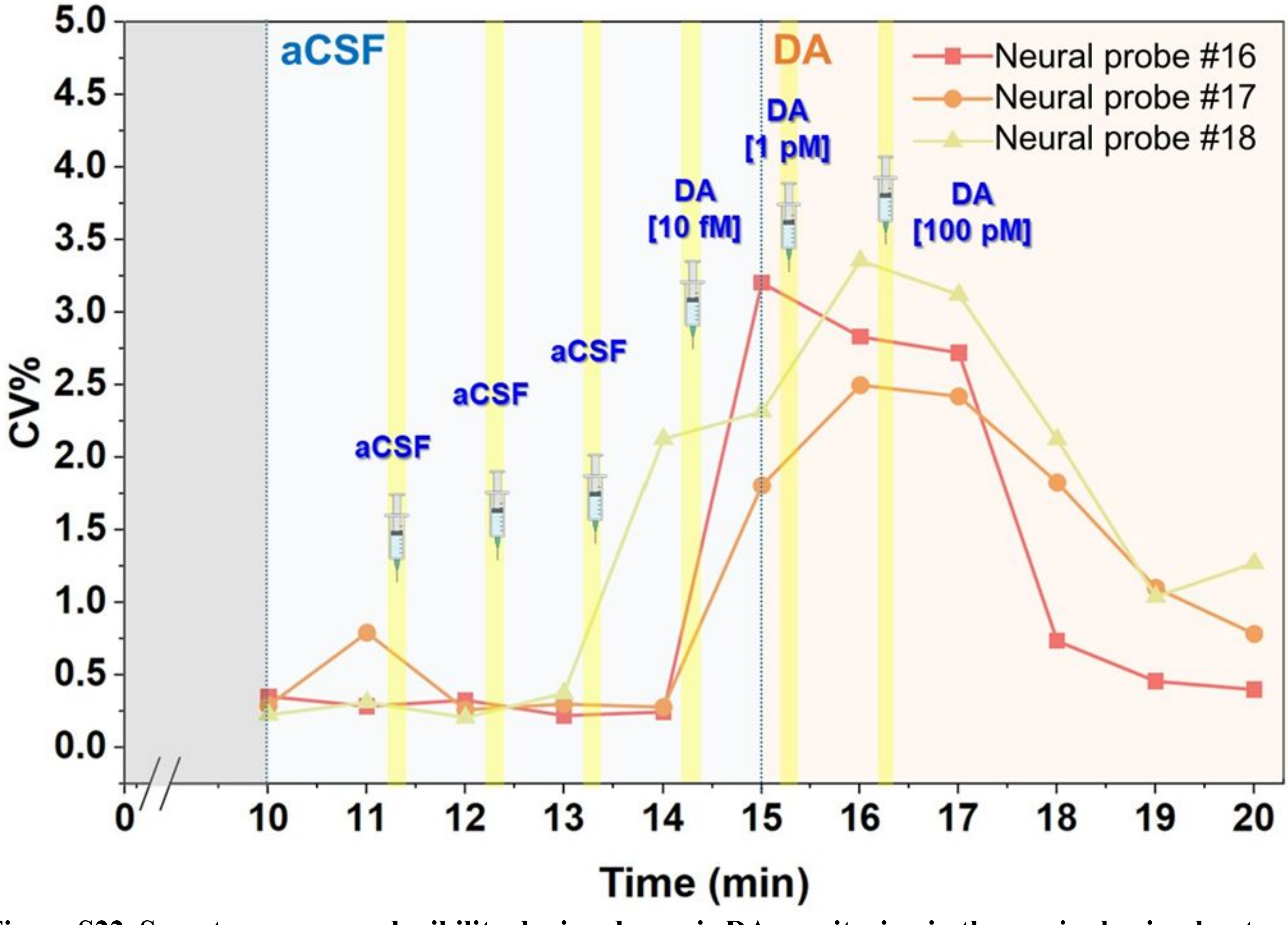

Figure S22. **Scan-to-scan reproducibility during dynamic DA monitoring in the *ex vivo* brain phantom**. The CV% for each electrochemical data point was calculated from three consecutive SWV scans acquired using a single sensing unit from three independently fabricated neural probes (#16~#18). During repeated aCSF injections, scan-to-scan variability remained consistently low (<1%). Following sequential injections of DA (10 fM, 1 pM, and 100 pM), the scan-to-scan CV% increased transiently due to dynamic changes in the local DA concentration during diffusion, and subsequently decreased as the concentration approached equilibrium. Throughout the experiment, all sensing units maintained scan-to-scan CV% values below 10%, confirming excellent measurement reproducibility under dynamic brain phantom conditions.

Note 13. Scan-to-scan reproducibility and inter-animal reproducibility of in vivo DA sensing

To further evaluate the reproducibility of the proposed multimodal neural probe under *in vivo* conditions, both intra-animal measurement stability and between-animal reproducibility were systematically assessed using three independently implanted neural probes (Neural probes #19-#21). Throughout each experiment, one electrochemical sensing unit was continuously operated to enable uninterrupted dynamic DA monitoring. Each electrochemical data point shown in **Figure S23a** represents the arithmetic mean of three consecutive SWV scans, whereas the corresponding scan-to-scan CV% was calculated from these three individual SWV measurements and is presented in **Figure S23b**.

The individual averaged DA response profiles in **Figure S23a** demonstrate that all animals exhibited the expected pharmacologically induced increase in extracellular DA following intravenous nomifensine administration, although noticeable differences in the temporal response profiles were observed. In particular, Rat #1 reached its maximum DA response earlier than Rats #2 and #3, indicating intrinsic inter-animal pharmacokinetic variability. To distinguish these biological differences from intrinsic measurement reproducibility, the corresponding scan-to-scan CV% values are presented in **Figure S23b**. Before nomifensine administration, the scan-to-scan CV% remained relatively low and stable, whereas modest transient increases were observed during the dynamic DA response following drug administration. Importantly, all implanted neural probes maintained scan-to-scan CV% values below 10% throughout the entire recording period, demonstrating excellent measurement reproducibility under dynamic *in vivo* neurochemical sensing conditions.

To quantitatively evaluate intra-animal measurement stability, the scan-to-scan CV% at the maximum DA response was further calculated for each animal and summarized in **Table S13**. DA currents were obtained from triplicate SWV scans during each measurement cycle (each scan consisting of a 3-sec SWV sweep followed by a 7-sec resting period), and the CV% was calculated from the three consecutive scans acquired at the peak response time point. As shown in **Table S13**, all animals exhibited peak-response CV% values below 10%, indicating excellent intra-measurement stability and confirming that the aptamer-based electrochemical sensing interface provides highly consistent electrochemical measurements within individual recording sessions[21].

The between-animal reproducibility of the *in vivo* neurochemical sensing performance was subsequently evaluated using the pharmacokinetic parameters summarized in **Table S14**. Across the three animals, the baseline SWV current exhibited an inter-animal CV% of 11.52%, while the peak SWV current showed an even lower inter-animal CV% of 7.68%, indicating good reproducibility of the electrochemical sensing performance across independently implanted neural probes[21]. In contrast, the time-to-peak current, defined as the duration between intravenous nomifensine administration and the appearance of the maximum SWV current response, exhibited a substantially larger inter-animal CV% of 33.25%. This greater temporal variability is biologically expected because the time-to-peak primarily reflects systemic pharmacokinetic processes, including drug absorption, DA transporter inhibition, extracellular DA accumulation, and individual physiological responses, rather than the intrinsic sensing capability of the electrochemical platform[22]. Minor differences in probe implantation location and local electrode-tissue interactions may also contribute to the observed temporal variability. Nevertheless, despite these differences in response timing, all animals exhibited a highly consistent pharmacologically induced DA response profile following nomifensine administration. Therefore, our proposed multimodal neural probe maintained excellent scan-to-scan reproducibility throughout dynamic *in vivo* neurochemical monitoring, while the relatively larger variability observed in the time-to-peak DA response primarily reflects normal pharmacokinetic heterogeneity among animals rather than instability of the electrochemical sensing interface.

Table S13. Intra-animal stability of *in vivo* DA sensing. Peak SWV current CV% was calculated from triplicate scans at the maximum response time point for each rat. All CV% values were below 10%, indicating excellent intra-measurement stability.

Animal ID	$Mean_{peak\ current}$ *(nA)*	$SD_{peak\ current}$ *(nA)*	*Intra-animal CV%*
**Rat #1**	21.8561	0.6592	3.02
**Rat #2**	21.0734	0.3325	1.58
**Rat #3**	18.2636	0.2265	1.24

Note: For each animal, DA current was obtained from triplicate SWV scans per measurement cycle (each scan consisting of a 3-sec sweep followed by a 7-sec rest period)

Table S14. Between-subject analysis of *in vivo* DA sensing reproducibility. Baseline SWV current, peak SWV current, and time to peak current obtained from individual animals (N = 3 rats). Inter-animal CV% values were calculated to evaluate reproducibility across animals.

Animal ID	$Mean_{baseline\ current}$ *(nA)*	$Mean_{peak\ current}$ *(nA)*	*Time to peak current (sec)*
**Rat #1**	14.6861	21.8561	420
**Rat #2**	15.2703	21.0734	840
**Rat #3**	12.2221	18.2636	770
**Mean ± SD**	14.06 ± 1.62	20.05 ± 1.54	676.67 ± 225.02
***Inter-animal* CV %**	11.52%	7.68%	33.25%

Note: Time to peak current was defined as the duration between the initiation of intravenous nomifensine administration and the appearance of the maximum SWV current response.

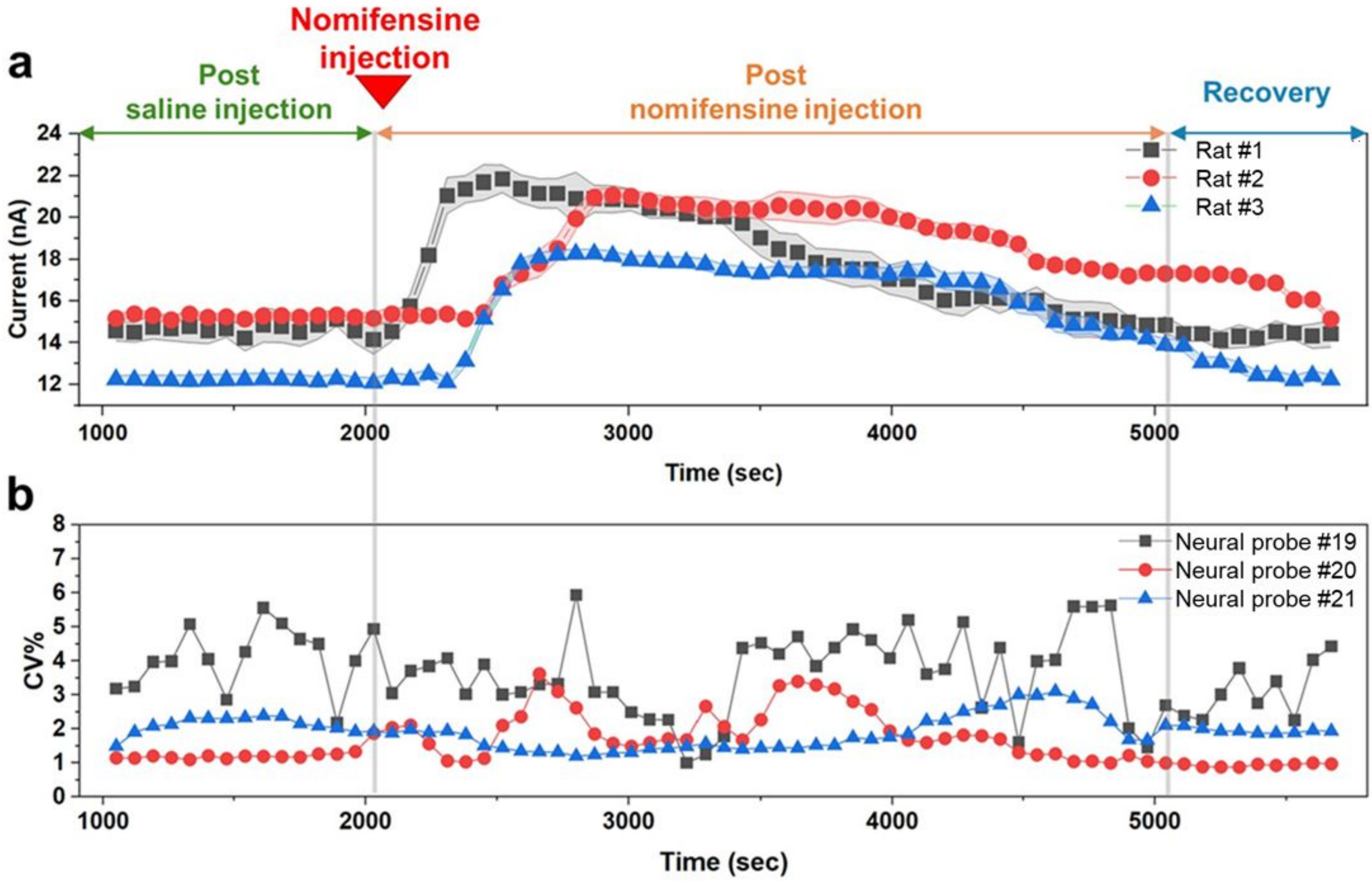

Figure S23. **Evaluation of scan-to-scan reproducibility during *in vivo* DA monitoring**. Individual averaged DA response profiles acquired from three individual rats following intravenous nomifensine administration using independently fabricated neural probes (one sensing unit per probe). Distinct temporal response profiles, particularly the earlier time-to-peak observed in Rat #1, indicate intrinsic inter-animal pharmacokinetic variability. The corresponding pharmacokinetic parameters are summarized in **Table S14**. (b) Individual scan-to-scan CV% values for neural probes #19-#21, calculated from three consecutive SWV scans at each electrochemical data point. Despite transient increases during dynamic DA responses, all sensing units maintained scan-to-scan CV% values below 10% throughout the recording period, indicating excellent *in vivo* measurement reproducibility.

Note 14. Specifications and schematics of the neural probe

Table S15. Geometric dimensions of the flexible multimodal microelectrode array probe, including overall probe geometry and electrode-level features.

Category	Parameter	Symbol	Value	Unit
**Probe geometry**	Number of sites	N/A	9	N/A
	Probe length	A	18.1	mm
	Probe width	B	2.66	mm
	Shaft length	C	14.87	mm
	Maximum shaft width	D	996	μm
	Shaft width	E	270	μm
	Tip width	F	3	μm
	Inter-site distance	G	354	μm
**Electrode geometry**	Site diameter	H	26	μm
	Metal wire width	I	10	μm
	Working electrode area	J	1,975	μm²
	Reference electrode area	K	17,320	μm²

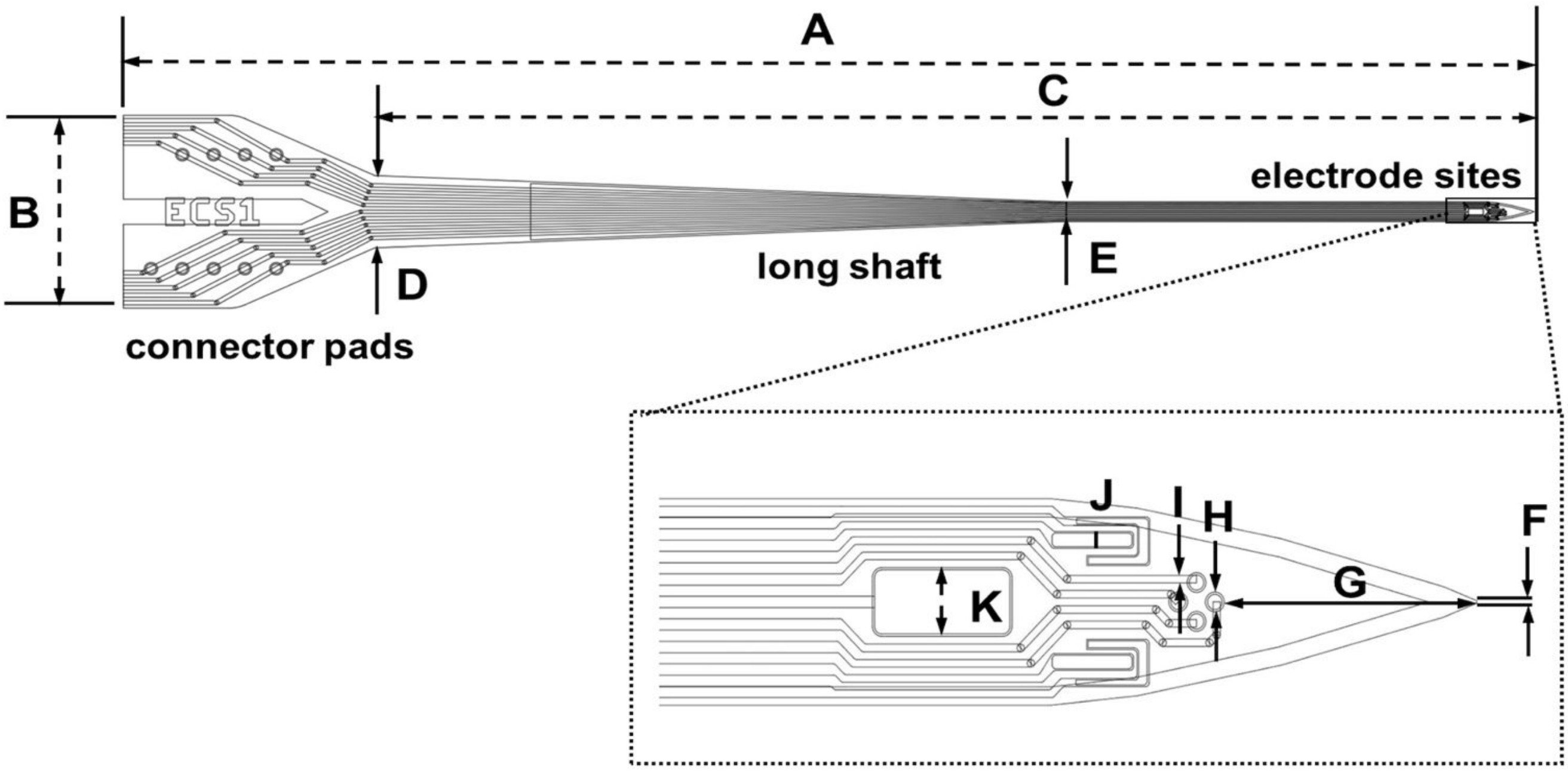

References:

(1) Trasatti, S.; Petrii, O. Real surface area measurements in electrochemistry. *Pure and applied chemistry* **1991**, *63* (5), 711-734. DOI: 10.1351/pac199163050711.

(2) Woods, R.; Bard, A. Chemisorption at Electrodes in Electroanalytical Chemistry. Marcel Dekker; New York: 1976; Vol. 9, pp 1-162.

(3) Johnston, H. J.; Hutchison, G.; Christensen, F. M.; Peters, S.; Hankin, S.; Stone, V. A review of the in vivo and in vitro toxicity of silver and gold particulates: particle attributes and biological mechanisms responsible for the observed toxicity. *Critical reviews in toxicology* **2010**, *40* (4), 328-346. DOI: 10.3109/10408440903453074. Hadrup, N.; Lam, H. R. Oral toxicity of silver ions, silver nanoparticles and colloidal silver–a review. *Regulatory Toxicology and Pharmacology* **2014**, *68* (1), 1-7. DOI: 10.1016/j.yrtph.2013.11.002.

(4) Wieckowski, A. *Interfacial electrochemistry: theory: experiment, and applications*; CRC press, 1999.

(5) Shinwari, M. W.; Deen, M. J.; Landheer, D. Study of the electrolyte-insulator-semiconductor field-effect transistor (EISFET) with applications in biosensor design. *Microelectronics Reliability* **2007**, *47* (12), 2025-2057. DOI: 10.1016/j.microrel.2006.10.003. Green, R. A.; Lovell, N. H.; Wallace, G. G.; Poole-Warren, L. A. Conducting polymers for neural interfaces: challenges in developing an effective long-term implant. *Biomaterials* **2008**, *29* (24-25), 3393-3399. DOI: 10.1016/j.biomaterials.2008.04.047.

(6) Abbas, Y.; Olthuis, W.; van den Berg, A. Activated carbon as a pseudo-reference electrode for electrochemical measurement inside concrete. *Construction and Building Materials* **2015**, *100*, 194-200. DOI: https://doi.org/10.1016/j.conbuildmat.2015.10.001. Ges, I. A.; Dzhura, I. A.; Baudenbacher, F. J. On-chip acidification rate measurements from single cardiac cells confined in sub-nanoliter volumes. *Biomed Microdevices* **2008**, *10* (3), 347-354. DOI: 10.1007/s10544-007-9142-7.

(7) Shinwari, M. W.; Zhitomirsky, D.; Deen, I. A.; Selvaganapathy, P. R.; Deen, M. J.; Landheer, D. Microfabricated reference electrodes and their biosensing applications. *Sensors (Basel)* **2010**, *10* (3), 1679-1715. DOI: 10.3390/s100301679 From NLM. Dawkins, R. C.; Wen, D.; Hart, J. N.; Vepsäläinen, M. A screen-printed Ag/AgCl reference electrode with long-term stability for electroanalytical applications. *Electrochimica Acta* **2021**, *393*, 139043. DOI: https://doi.org/10.1016/j.electacta.2021.139043.

(8) Watkins, Z.; Karajic, A.; Young, T.; White, R.; Heikenfeld, J. Week-long operation of electrochemical aptamer sensors: new insights into self-assembled monolayer degradation mechanisms and solutions for stability in serum at body temperature. *ACS sensors* **2023**, *8* (3), 1119-1131. DOI: 10.1021/acssensors.2c02403. Bard, A. J.; Faulkner, L. R.; White, H. S. *Electrochemical methods: fundamentals and applications*; John Wiley & Sons, 2022.

(9) Lee, H.; Dellatore, S. M.; Miller, W. M.; Messersmith, P. B. Mussel-inspired surface chemistry for multifunctional coatings. *science* **2007**, *318* (5849), 426-430. DOI: 10.1126/science.1147241. Liu, Y.; Ai, K.; Lu, L. Polydopamine and its derivative materials: synthesis and promising applications in energy, environmental, and biomedical fields. *Chem Rev* **2014**, *114* (9), 5057-5115. DOI: 10.1021/cr400407a.

(10) Lubin, A. A.; Plaxco, K. W. Folding-based electrochemical biosensors: the case for responsive nucleic acid architectures. *Accounts of chemical research* **2010**, *43* (4), 496-505. DOI: 10.1021/ar900165x. Xiao, Y.; Lubin, A. A.; Heeger, A. J.; Plaxco, K. W. Label-free electronic detection of thrombin in blood serum by using an aptamer-based sensor. *Angewandte Chemie* **2005**, *117* (34), 5592-5595. DOI: 10.1002/anie.200500989. Radi, A.-E.; Acero Sánchez, J. L.; Baldrich, E.; O'Sullivan, C. K. Reagentless, reusable, ultrasensitive electrochemical molecular beacon aptasensor. *Journal of the American Chemical Society* **2006**, *128* (1), 117-124. DOI: 10.1021/ja053121d.

(11) Robinson, D. L.; Hermans, A.; Seipel, A. T.; Wightman, R. M. Monitoring rapid chemical communication in the brain. *Chemical reviews* **2008**, *108* (7), 2554-2584. DOI: 10.1021/cr068081q.

(12) Arroyo-Currás, N.; Somerson, J.; Vieira, P. A.; Ploense, K. L.; Kippin, T. E.; Plaxco, K. W. Real-time measurement of small molecules directly in awake, ambulatory animals. *Proc Natl Acad Sci U S A* **2017**, *114* (4), 645-650. DOI: 10.1073/pnas.1613458114 From NLM. Lubin, A. A.; Plaxco, K. W. Folding-based electrochemical biosensors: the case for responsive nucleic acid architectures. *Acc Chem Res* **2010**, *43* (4), 496-505. DOI: 10.1021/ar900165x From NLM. Xiao, Y.; Lubin, A. A.; Heeger, A. J.; Plaxco, K. W. Label-free electronic detection of thrombin in blood serum by using an aptamer-based sensor. *Angew Chem Int Ed Engl* **2005**, *44* (34), 5456-5459. DOI: 10.1002/anie.200500989.

(13) Jin, H.; Zhao, C.; Gui, R.; Gao, X.; Wang, Z. Reduced graphene oxide/nile blue/gold nanoparticles complex-modified glassy carbon electrode used as a sensitive and label-free aptasensor for ratiometric electrochemical sensing of dopamine. *Anal Chim Acta* **2018**, *1025*, 154-162. DOI: 10.1016/j.aca.2018.03.036.

(14) Beiranvand, S.; Abbasi, A. R.; Roushani, M.; Derikvand, Z.; Azadbakht, A. A simple and label-free aptasensor based on amino group-functionalized gold nanocomposites-Prussian blue/carbon nanotubes as labels for signal amplification. *Journal of Electroanalytical Chemistry* **2016**, *776*, 170-179. DOI: 10.1016/j.jelechem.2016.07.006.

(15) Lu, L.; Guo, L.; Kang, T.; Cheng, S. A gold electrode modified with a three-dimensional graphene-DNA composite for sensitive voltammetric determination of dopamine. *Microchimica Acta* **2017**, *184* (8), 2949-2957. DOI: 10.1007/s00604-017-2267-3.

(16) Azadbakht, A.; Roushani, M.; Abbasi, A. R.; Derikvand, Z. Design and characterization of electrochemical dopamine-aptamer as convenient and integrated sensing platform. *Anal Biochem* **2016**, *507*, 47-57. DOI: 10.1016/j.ab.2016.04.022.

(17) Talemi, R. P.; Mousavi, S. M.; Afruzi, H. Using gold nanostars modified pencil graphite electrode as a novel substrate for design a sensitive and selective Dopamine aptasensor. *Mater Sci Eng C Mater Biol Appl* **2017**, *73*, 700-708. DOI: 10.1016/j.msec.2016.12.119.
(18) Wang, W.; Wang, W.; Davis, J. J.; Luo, X. Ultrasensitive and selective voltammetric aptasensor for dopamine based on a conducting polymer nanocomposite doped with graphene oxide. *Microchimica Acta* **2015**, *182* (5), 1123-1129. DOI: 10.1007/s00604-014-1418-z.
(19) Taheri, R. A.; Eskandari, K.; Negahdary, M. An electrochemical dopamine aptasensor using the modified Au electrode with spindle-shaped gold nanostructure. *Microchemical Journal* **2018**, *143*, 243-251. DOI: 10.1016/j.microc.2018.08.008.
(20) Li, X.; Jin, Y.; Zhu, F.; Liu, R.; Jiang, Y.; Jiang, Y.; Mao, L. Electrochemical Conjugation of Aptamers on a Carbon Fiber Microelectrode Enables Highly Stable and Selective In Vivo Neurosensing. *Angew Chem Int Ed Engl* **2022**, *61* (42), e202208121. DOI: 10.1002/anie.202208121.
(21) Aronhime, S.; Calcagno, C.; Jajamovich, G. H.; Dyvorne, H. A.; Robson, P.; Dieterich, D.; Fiel, M. I.; Martel-Laferriere, V.; Chatterji, M.; Rusinek, H.; et al. DCE-MRI of the liver: effect of linear and nonlinear conversions on hepatic perfusion quantification and reproducibility. *J Magn Reson Imaging* **2014**, *40* (1), 90-98. DOI: 10.1002/jmri.24341.
(22) Dauer, W.; Przedborski, S. Parkinson's disease: mechanisms and models. *Neuron* **2003**, *39* (6), 889-909. DOI: 10.1016/S0896-6273(03)00568-3 . Grace, A. A. Dysregulation of the dopamine system in the pathophysiology of schizophrenia and depression. *Nature Reviews Neuroscience* **2016**, *17* (8), 524-532. DOI: 10.1038/nrn.2016.57.
